# Drivers of health equity in the Americas: a scoping review

**DOI:** 10.1186/s12939-026-03031-6

**Published:** 2026-09-16

**Authors:** Evelina Chapman, Miguel Araujo, Rojas-Botero Maylen, Jordi Pardo Pardo, Oscar J. Mujica

**Affiliations:** 1 Evidence and Intelligence for Action in Health, Pan American Health Organization, Washington, DC USA; 2 Campbell and Cochrane Health Equity Thematic Group, University of Ottawa, Ottawa, ON Canada

**Keywords:** Health equity, Social determinants of health, Driver, PROGRESS-PLUS

## Abstract

**Background:**

The term “driver” is often used inconsistently in the literature, referring to diverse analytical levels, from structural determinants to individual risk factors, frequently without a clear distinction. This lack of precision challenges theoretical consistency and health equity operationalization. This scoping review aims to harmonize the conceptual meanings of “drivers,” their relationship with social determinants of health (SDoH), methodological strategies, and research gaps within the Americas.

**Methods:**

Following PRISMA-ScR guidelines, 286 documents (2008–2026) were analyzed. The review utilized the JBI PCC framework to address four research questions on the global and regional application of “drivers”. The general population was analyzed across PROGRESS-Plus axes, treated as relational dimensions of inequality that structure social positions. Data synthesis integrated descriptive mapping with conceptual analysis of drivers and their interplay with SDoH.

**Results:**

Findings reveal a conceptual evolution toward “Social Drivers of Equity in Health” (SDEH) as operational engines of power. While 36.4% of the literature uses “driver” and “determinant” interchangeably, 38.5% identifies drivers as independent forces putting SDoH in motion. Methodologically, 33.3% were primary quantitative studies; 46 utilized multivariate modeling to isolate structural effects. In the Americas, structural racism (80.0%) and socioeconomic status (79.3%) predominate as primary engines. PROGRESS-Plus analysis identified emerging axes, such as digital and commercial dimensions, mediating geographic inequities. A significant “research inequity” persists, with 79.3% of the regional evidence concentrated in the United States, leaving substantial knowledge gaps regarding the structural drivers specific to Latin America and the Caribbean.

**Conclusions:**

While the term “driver” presents tensions with established SDoH models, adopting a “hierarchy of drivers” (Upstream-Midstream-Downstream) could offer a pragmatic framework for identifying precise levers of power, such as legal reforms and social protection. The term should be used cautiously, ensuring its articulation with robust theoretical frameworks to act as an operational catalyst for structural intervention.

**Registration:**

The protocol was registered on the Open Science Framework platform under ID 48azj.

**Supplementary Information:**

The online version contains supplementary material available at https://doi.org/10.1186/s12939-026-03031-6.

## Introduction

The study of circumstances that determine health and well-being has evolved through various reference frameworks designed to dismantle systemic inequities. It is widely recognized that biological factors contribute minimally to population health inequities compared with the profound weight of social and economic determinants [1]. Seminal models, such as the WHO Commission on Social Determinants of Health, WHO-CSDH [2] and the Spanish Commission model to reduce Social Inequalities in Health [3], established the foundational architecture for understanding these inequities. While foundational socio-ecological paradigms like the Dahlgren-Whitehead “rainbow” model (1991) [4] established the concentric stratification of health influences, from the micro-level individual lifestyle factors to the macro-level political, socioeconomic, cultural, and environmental conditions [5], the Marmot and WHO-CSDH framework was explicitly prioritized in this scoping review [6–8]. Unlike the predominantly stratified, descriptive nature of concentric models, WHO-CSDH/Marmot’s framework explicitly models the social production of health inequalities, placing special emphasis on the political, macroeconomic, and structural mechanisms that govern the distribution of power, money, resources, and prestige; i.e., the “causes of the causes” [9, 10]. Because the core analytical objective of this review is to isolate *drivers of health equity* as actionable, kinetic engines capable of policy modification rather than merely mapping health influences across levels of organization, Marmot’s structural-mechanistic architecture provided the necessary theoretical grounding to conceptualize drivers as operational catalysts of inequity [11]. Since Whitehead’s [12] landmark definition of health equity as the absence of unfair and avoidable differences, health inequalities have gained increasing prominence in international and national policy agendas, prompting efforts to better conceptualize, measure, and address them [1, 13, 14].

Despite the 2015 global commitment to “leave no one behind” embedded in the 2030 Agenda for Sustainable Development [15], the reduction of health inequalities was not explicitly detailed across its 169 targets. However, the COVID-19 pandemic acted as a catalyst, forcing a global recognition of the urgency to advance pro-equity monitoring and policies [16–18]. The crisis made it evident that static, linear models are insufficient to capture the complexity of modern crises; there is an urgent need for dynamic frameworks that utilize a syndemic perspective to (re)conceptualize vulnerability in a rapidly changing world [19].

From a global perspective, the mechanisms of equity vary significantly across regions. While the European Region [20] has prioritized policy coherence and community empowerment as main “drivers”, the Americas –consistently ranked as one of the most inequitable regions in the world– identify ongoing colonialism, structural racism, incarceration systems, and commercial influences as the primary engines of disparity [13].

In this context, a critical conceptual evolution has emerged. While the framework of SDoH has historically oriented action toward social structures, recent discussions suggest the need for a more granular focus on the mechanisms of change. Rather than a departure from SDoH, the emerging nomenclature of “drivers of health equity” functions as a conceptual refinement that emphasizes the kinetic and actionable nature of these factors [21].

This perspective aligns with recent international frameworks (e.g., WHO/EURO 2019) that identify “drivers” as the institutional and structural engines necessary to operationalize SDoH theory into measurable progress.

The aim of this scoping review is to clarify the use of the term “driver,” its conceptual evolution, and the operational relationship between “drivers” and social determinants of health (SDoH). Focusing on literature relevant to the Americas, the study also aims to identify region-specific drivers of health equity, examine the methodological strategies and theoretical frameworks used to identify them, and detect critical research gaps.

### Research questions

1. What are the definitions, meanings, and conceptual relationships between drivers of health equity and social determinants of health (SDoH) on a global scale? (Q1)
2. What are the drivers of health equity identified in the literature of the Americas? (Q2)
3. What are the methodological strategies and theoretical frameworks used to identify the drivers of health equity in the Americas? (Q3)
4. What are the research gaps in the Americas as identified in the included documents? (Q4)

## Methods

### Protocol and registration

This scoping review was conducted in accordance with the recommendations of Preferred Reporting Items for Systematic Reviews and Meta-Analyses: Extension for Scoping Reviews (PRISMA-ScR) [22] and the Joanna Briggs Institute (JBI) guidelines [23]. The protocol was registered on the Open Science Framework (OSF) Website [24].

### Eligibility criteria

We utilized the JBI PCC (Population, Concept, Context) framework.

### Population

The review included the general population, analyzed across PROGRESS-Plus stratifiers [25] and other emerging equity-related characteristics (e.g., migration status, specific health conditions). These dimensions were treated not as labels for specific sub-populations, but as transversal and relational axes of social inequality that structure social positions and health opportunities. This approach allowed for the inclusion of both broad population-level data and evidence centered on groups whose health outcomes are compromised by intersecting structural drivers.

### Concept

Elements identified as “drivers” of health equity and their relationship with SDoH. Although no single, standardized definition of “drivers of health equity” was identified in the literature, the review identified a consistent set of paradigms linking social drivers to equity frameworks. Consequently, to avoid the imprecise use of “drivers” as a mere synonym for various determinants or mediators, this study adopts the acronym SDEH (social drivers of equity in health) as an operational construct. This nomenclature is used to distinguish macro-structural engines of power from traditional SDoH, which represent the resulting social circumstances and intermediary processes.

Context: Question 1 was global (including the Americas); Questions 2–4 focused on the Americas.

### Inclusion and exclusion criteria

#### Inclusion criteria

Records published between 2008 (the year the WHO [1] defined the social determinants of health) and January 2026 were included. The search encompassed documents in English, Spanish, and Portuguese. All study designs were considered, including primary research (qualitative, quantitative, and mixed methods), systematic and scoping reviews, narrative reviews, expert reports, book chapters, and theoretical essays, to capture the conceptual evolution of “drivers” across both academic and grey literature.

To ensure conceptual relevance, the inclusion of the term “driver” was strictly conditional upon its role as a central object of study. Specifically, the term had to be explicitly linked to health equity or social determinants of health, functioning as a primary analytical variable rather than a generic descriptor. This required the term to appear consistently within the study’s core analysis (e.g., methods, discussion, or results sections), rather than as an isolated mention within the titles, abstracts, or introductory paragraphs.

#### Exclusion criteria

Studies focused exclusively on biological, clinical, or individual-level factors without a broader social, structural, or population health perspective were excluded. For the Americas context (Q2-Q4), publications that did not provide specific information on drivers within the region were excluded. Editorials, abstracts, letters to the editor, study protocols, trial registries, publications without full-text access, and opinion pieces from non-institutional sources (e.g., personal blogs) were also excluded. Finally, documents that used the word “driver” as a non-specific placeholder or as a synonymous “filler” for general health influences were excluded.

### Information sources and search strategy

The search strategy was designed to maintain high sensitivity. An initial base strategy was developed for Medline (PubMed) and subsequently adapted for other databases using the Polyglot Search tool. The syntax focused on the intersection of the term “driver” with “equity” and “social determinants” across six databases: Medline, EMBASE, CINAHL, LILACS, Epistemonikos, and Campbell Collaboration. Grey literature was identified through specific institutional websites (PAHO, NIH, CIHR) and Google Scholar (the first 200 records, sorted by relevance). Reference lists of included reviews were manually searched. The searches covered the period 2008–January 2026.

### Selection of sources of evidence

Selection was performed by two independent reviewers (EC, MA). An initial inter-observer analysis on a random sample of 200 records yielded a high level of agreement (Kappa = 0.875 for Q1; 0.723 for Q2-Q4). Consequently, the remaining screening was distributed among the reviewers, multiple cross-checks were carried out to ensure the greatest possible consistency, with doubtful cases resolved by consensus.

### Data charting process

Data extraction was performed using a single-reviewer approach, where the database was distributed among the reviewers (EC, MA, MR-B) using a standardized form consisting of pre-defined categorical variables and free-text fields to capture the heterogeneity of the source documents. The quality and consistency of the extraction were ensured through a multi-stage verification process led by two reviewers (EC, MA).

During the data cleaning phase and before the analysis, a selective double-check was performed on the most complex variables or where inconsistencies were detected.

### Critical appraisal of individual sources of evidence

In accordance with scoping review standards, a formal risk of bias assessment was not applied across all included documents. However, for quantitative and empirical studies, the presence of covariate adjustment or confounding control procedures was recorded to differentiate between descriptive and analytical approaches to driver identification (Q2&Q3). For conceptual publications and essays, the appraisal focused on the institutional relevance of the source and the internal coherence of the proposed theoretical framework.

### Data synthesis

For the general findings and each specific research question, results are presented through tables and a narrative synthesis, complemented by a bibliometric analysis.

To ensure a systematic organization of the evidence, a database was developed to capture a wide array of variables, beginning with a global mapping of definitions and conceptual meanings for Q1. For the regional sample of the Americas (covering Q2–Q4), a more granular data extraction process was performed to identify and analyze specific drivers as the primary focus. This involved examining their directionality, the theoretical frameworks employed (both established and emerging), and the methodological strategies used. To further characterize these drivers, the PROGRESS-Plus framework was applied to account for axes of inequality such as Place of residence, Race/ethnicity, Occupation, Gender, Religion, Education, Social capital, Socioeconomic status, Age, Disability, and Sexual orientation. Finally, the regional analysis included the systematic identification of research gaps.

Data synthesis was conducted entirely by the research team using a qualitative codebook thematic analysis approach, combining deductive mapping with inductive thematic development. Extracted text, conceptual definitions, and empirical data were manually coded by two independent reviewers (EC, MA) using the standardized charting form, with coding consistency established through initial inter-observer screening (Kappa = 0.875 for Q1; 0.723 for Q2–Q4). First-order codes captured mechanisms, contexts, and populations across PROGRESS-Plus axes. Thematic categories were developed deductively from established health equity domains (e.g., structural racism, commercial determinants) and inductively from emerging concepts (e.g., algorithmic bias, necropolitics).

Following complete human data extraction and primary manual coding, Gemini (Google) was utilized exclusively as an auxiliary analytical tool to assist in organizing and standardizing categorical groupings (e.g., verifying the classification of driver impact directionality as positive, negative, or bidirectional, and supporting the clustering of theoretical frameworks) (see prompt documentation in Supplementary File 5). All AI-supported groupings were systematically inspected, cross-checked against original extraction sheets, and verified by the first author, who maintained full analytical authority and finalized all classifications.

## Results

### Selection of sources of evidence

**Fig. 1:**
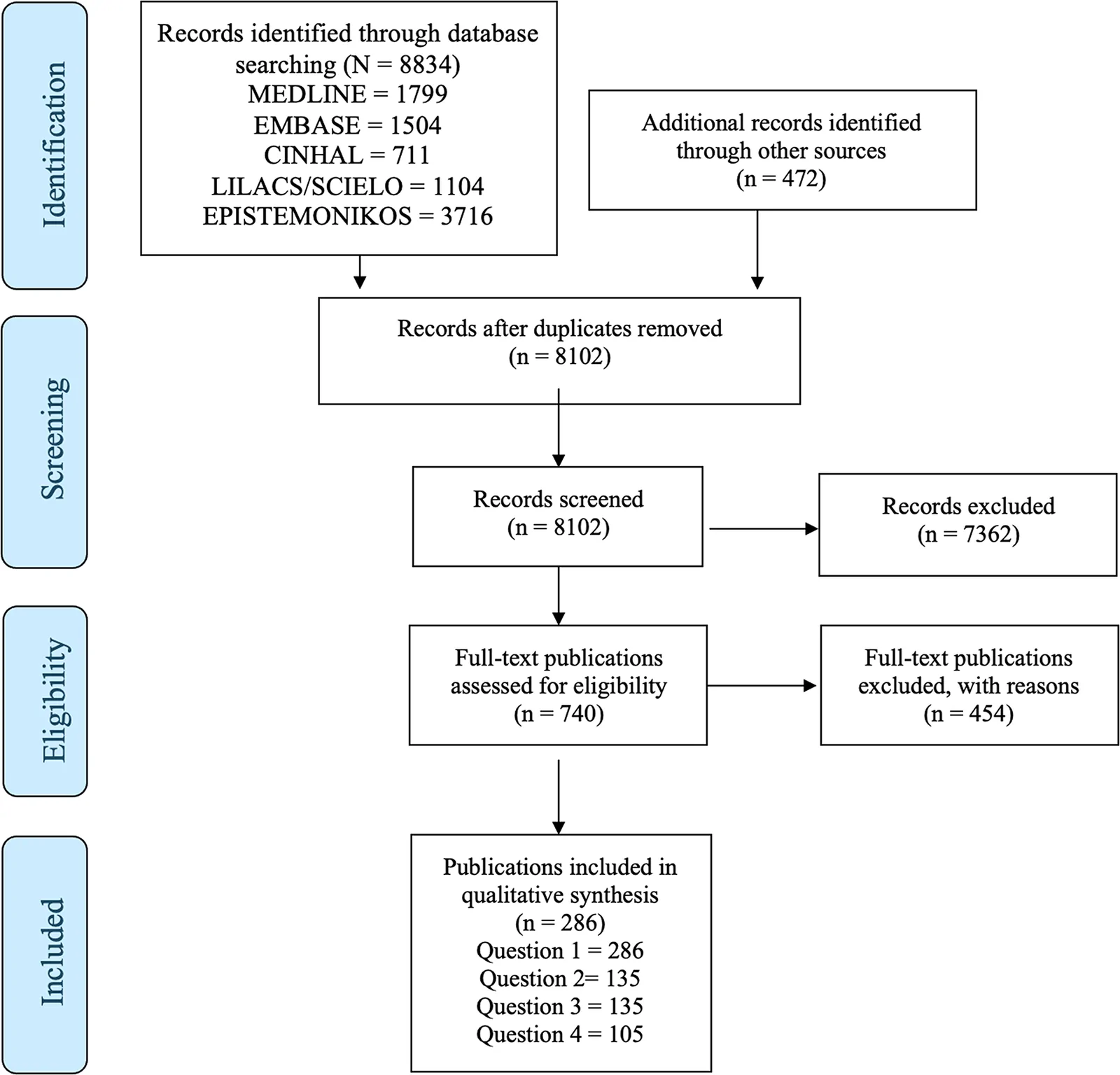
PRISMA flowchart of the selection of publications

Figure 1 summarizes the search and selection process. The initial identification phase across all sources yielded the total volume of records described in the methodology (See details of searches in Supplementary File 1). After removing duplicates and screening titles and abstracts, 843 publications were identified as potentially eligible. Following the full-text review phase, 557 records were excluded based on the predefined criteria (detailed in Supplementary File 3). The final sample consists of 286 unique publications (hereafter referred to as “documents” or “publications” to reflect the diversity of evidence types). The full list of included studies is provided in Supplementary File 2, while the list of excluded studies is available in Supplementary File 3.

### Characteristics of sources of evidence

Of the 286 publications identified, 135 (47.2%) were from the Americas. The evidence base primarily comprised 278 (98.2%) scientific journal articles (including preprints), and 8 as grey literature (2.8%). English was the predominant language in 281 (98.2%), Portuguese in 2 (0.7%), and 3 in Spanish (1.1%).

All 286 included documents contributed to the analysis of definitions, meanings, and the conceptual interplay between drivers and SDoH (Q1), while the regional assessment (Q2–Q4) was restricted to the Americas stratum (*n* = 135). Within this subset, specific drivers and their directionality were identified across all 135 publications (Q2). Methodological strategies and theoretical frameworks, theories, or models (FTM) (Q3) were also analyzed for the complete regional stratum (*n* = 135). Finally, explicit research gaps (Q4) were identified and synthesized from 105 of these documents (78%), representing the core evidence for the thematic lacunae analysis. Detailed analyses supporting these points are provided in Supplementary File 5 and Supplementary File 6 (PROGRESS-PLUS Analysis).

### Synthesis of results

We present the narrative synthesis by research question.

#### Question 1: Definitions and the conceptual relationship with SDoH (Global Focus)

The analysis of the 286 documents reveals that the conceptualization of “drivers” is heterogeneous, encompassing uses that range from a synonym for SDoH to more structured interpretations related to structural power. We identified that formal definitions of the term “drivers” are not uniform, appearing explicitly in only 16 of the analyzed documents. While these represent “textual” definitions, it is important to note that other publications may utilize the term implicitly without providing a verbatim formalization.

Of these 16 definitions, nine approach the concept from a general or systemic perspective. This group includes macro-structural frameworks (e.g., Kruize, 2014; Mercado, 2024), institutional governance (WHO-EURO, 2019), and commercial or political power dynamics (Baker, 2025). The remaining seven definitions apply the term to specific domains or health conditions. These include clinical or biological focuses such as HIV vulnerability (Auerbach, 2011), the social exposome of biological aging (Nielsen, 2024), or maternal health inequities (De Ornelas, 2025). Additionally, this group encompasses more specialized systemic views, such as biographical drivers (Badal, 2023) and political determinants as the root causes of specific disparities (Savage, 2024). As shown in Table 1, the conceptualization of “drivers” varies significantly by regional focus.

A comprehensive list of all 16 identified definitions is provided in Supplementary File 4.

**Table 1:** Representative global and regional definitions of health equity drivers

Study	Location	Focus	Definition / Conceptual approach
WHO-EURO (2019)[20]	Global	General	**Institutional drivers of health equity**: “The four key drivers of health equity are: accountability, policy coherence, social participation and, underlying them, empowerment.”
Kruize (2014)[26]	Global	General	**Structural drivers**: “…comprise the social, economic, and political mechanisms that create socioeconomic stratification, including the labor market, the educational system, political institutions and other cultural and societal values.”
Baker (2025)[27]	Global	Specific (Commercial)	**Commercial and political power drivers**: “…the growing economic and political power of the industry, and its restructuring of food systems for profitability above all else…. in an increasingly financially-driven, capitalist world economy.”
Mercado (2024)[28]	Americas	General	**Structural drivers**: “Structural drivers are the macro-level systems (law, policy, economy) that shape the distribution of SDOH.”
Serchen (2021)[29]	Americas	General	**Social drivers of health**: “Social drivers of health, such as education, environment, employment, housing, and access to healthful foods… all directly and indirectly contribute to a person’s health status.”
De Ornelas (2025)[30]	Americas	Specific (Maternal)	**Drivers of racial inequity in health**: Focuses on whether “state laws and regulations… sought to directly intervene on healthcare-based drivers of racial inequities in maternal health.”

##### Conceptual relationship and interplay between SDHE and SDoH

The conceptual interplay between SDHE and SDoH revealed heterogeneous and frequently overlapping patterns of interpretation; four broad patterns were identified: equivalence, operational linkage, systemic “causality”, and structural frameworks. Data showed that 36.4% of the literature utilized the terms as functional synonyms (Equivalence), reflecting a trend of terminological alignment between both concepts. Conversely, a significant portion of the evidence (38.5%) framed drivers as active forces that operated upon social determinants (Operational linkage); in this approach, drivers were identified as independent, actionable concepts that were distinct from SDoH. Furthermore, the data indicated a strong relation between drivers and the production of inequities (Systemic “Causality”). Across all regions, 81.5% of the analyzed documents identified drivers as factors that “caused disparities,” with 30.1% explicitly categorizing them as “structural” in nature. While the evidence focused primarily on identifying these systemic catalysts, only 7.0% of the total sample described them as “modifiable,” a finding that reinforced the structural weight of the identified drivers. Table 2 summarizes these conceptual relationships categorized by regional and global contexts.

**Table 2:** Interpreted conceptual relationship and interplay between drivers (SDHE) and SDoH (*n* = 286)

Conceptual relationship	Focus	Prevalence (%)	Description and representative references
Equivalence or Synonymy	Global / Americas	36.4%	Drivers are used interchangeably with SDoH to describe social conditions. (e.g., Serchen 2021, Auerbach 2011, Dalsania 2020).
Operational Linkage	Americas	38.5%	Drivers are independent concepts acting as forces or “levers” that shape SDoH. (e.g., Evans 2023, López 2022, Barnes 2022).
Systemic “Causality"*	Global / Americas	81.5%	Drivers are root factors and political forces responsible for “causing” health disparities. (e.g., Savage 2024, Abubakar 2022, Bailey 2022).
Structural Framework	Americas	30.1%	Focuses on macro-level systems (law, policy, economy) and historical mechanisms. (e.g., Mercado 2024, WHO-PAHO 2019, De Ornelas 2025).

* “Causality” denotes the kinetic power of macro-level engines in producing disparities, reflecting structural accountability rather than a traditional epidemiological association

##### The Americas focus (Q2-Q4)

###### General considerations

Within this regional sample (*n* = 135), the evidence was highly concentrated in the United States, which accounted for 107 publications (79.3%), followed by Canada with 8 (5.9%). The Latin America and the Caribbean (LAC) sub-region was represented by 16 publications (11.9%); within LAC, Brazil was the leading contributor with 3 documents (2.2%), followed by Peru with 2 (1.5%), and Mexico, Haiti, and the Dominican Republic with one publication each (0.7% each). Additionally, 6 publications (4.4%) addressed the LAC region at a sub-regional level, and 5 publications (3.7%) adopted a Pan-American scope without focusing on a single country.

Thematic analysis reveals that a significant portion of the literature addresses general health equity and structural drivers (52.6%). Furthermore, the remaining drivers are described within more specific scenarios, represented by studies focusing on COVID-19 (8.1%), chronic non-communicable diseases (4.4%), or maternal and child health (4.4%). Regarding the population focus, 20.0% of the studies specifically target ethnic and racial minorities; this trend is particularly pronounced in the United States, where the examination of racial disparities dominates the research agenda.

#### Question 2: Drivers of health equity in the Americas

To analyze the nature of the identified drivers, they were classified according to their impact on health equity into three categories: negative, positive, and bidirectional (context-dependent). Further, the drivers were organized based on their primary focus: (a) General Focus drivers encompassed cross-cutting structural or institutional processes, such as systemic racism, public policies, or environmental justice; while (b) Specific Focus drivers were those directly linked to particular diseases (e.g., renal disease), life cycles (e.g., aging, teen pregnancy), or social contexts (e.g., gentrification, commercial dynamics), among others.

The analysis showed that Specific Focus drivers represented 51.9% (*n* = 70) of the evidence, while General Focus drivers (including structural, institutional, and other cross-cutting processes) accounted for 48.1% (*n* = 65). As detailed in Table 3, bidirectional forces were frequent across all categories; however, Specific Focus drivers exhibited a higher prevalence of negative impacts (48.6%) compared to the general domains. Within the General Focus (Structural/Institutional) category, negative engines were also significant (35.8%), primarily reflecting how institutional frameworks and systemic biases act as persistent barriers to equity in the region.

**Table 3:** Categorization of health equity drivers by thematic domain and nature of impact (*n* = 138)

Analytical focus*	Total (*n*)	Negative *n* (%)	Positive *n* (%)	Bidirectional *n* (%)
**General Focus**	65	23 (35.4%)	3 (4.6%)	39 (60.0%)
Structural & Institutional	53	19 (35.8%)	3 (5.7%)	31 (58.5%)
Other Cross-cutting Processes	12	4 (33.3%)	0 (0.0%)	8 (66.7%)
**Specific Focus**	70	34 (48.6%)	1 (1.4%)	35 (50.0%)
Topic, Condition, or Social Context	70	34 (48.6%)	1 (1.4%)	35 (50.0%)
**TOTAL**	**135**	**57 (42.2%)**	**4 (3.0%)**	**74 (54.8%)**

* Representative authors for each category include: General Focus (Structural & Institutional), Adamkiewicz (2011) (environmental justice and housing policy) and Alkureishi (2021) (digital divide and literacy); Other Cross-cutting Processes, Bailey (2022) (structural mechanisms of inequity) and Chandra (2016) (community-level drivers); Specific Focus (Topic, Condition, or Social Context), Agarwal (2020) (diabetes and technology access) and Barber (2018) (cardiovascular health and neighborhood context)

While structural racism emerged as a pervasive negative upstream driver within the General Focus category, it also manifested as a specific engine of inequity when intersected with specific health domains. In these Specific Focus contexts, racism was identified, for example, through clinical biases in neonatology and perinatal care [26], as well as through systemic barriers in the management of infectious diseases such as HIV and COVID-19 [27, 28]. In the Structural and Institutional sub-domain (*n* = 53), environmental justice and public policy were identified as critical forces; for instance, institutional strategies in housing and neighborhood-level factors were documented as structural forces that shaped regional inequities [29, 30]. Furthermore, Technology and AI revealed high bidirectional effects, where technological engines could either exacerbate “digital redlining” or offer “data democratization” [31, 32]. Regarding the Specific Focus category (*n* = 70), the evidence was primarily concentrated within topic-related domains where structural drivers interacted with specific health conditions or social contexts. Within Chronic Disease and Infectious Disease contexts, “market dynamics,” commercial interests, and residential segregation acted as primary negative engines [33, 34]. Conversely, in Maternal and Reproductive contexts, drivers were frequently identified as “institutional mistreatment” and systemic racism [26, 35]. Finally, the analysis provided evidence of midstream drivers –such as biological “weathering” or psychological stress– acting as the pathways through which these structural engines produced clinical disparities [36, 37]. The full list of drivers per study is provided in Supplementary File 5.

##### Operationalizing dimensions of social stratification through the PROGRESS-plus framework

To move from conceptual definitions to actionable data, we operationalized dimensions of social stratification using an adapted PROGRESS-Plus framework. Given the high degree of intersectionality among variables, Table 4 presents a selection of the most prominent axes that act as primary engines of inequity in the region. While Socioeconomic Status (SES) and Ethno-racial identity (80.0%) represent the most prevalent strata, this concentration is heavily influenced by research volume and geographic distribution, as most included studies originate from the United States (78.5%). Notably, other dimensions such as Education (45.2%) and Age (40.0%) also showed significant frequencies, highlighting the multi-layered nature of stratification in the sample.

A methodological adaptation for this study was the integration of Gender and Sex (37.8%) into a unified transversal axis. Additionally, the results highlight the emergence of a Digital and Commercial dimension (9.6%), which conceptualizes how geographic Place of Residence (68.9%) is now inextricably mediated by digital infrastructure. The complete distribution of all 18 PROGRESS-Plus variables identified across the 135 studies is provided in Supplementary File 6.

**Table 4:** Selected PROGRESS-plus axes and associated structural drivers (*n* = 135)

Adapted PROGRESS-Plus Category	Frequency *n* (%)	Primary Structural Driver	Nature of Effect	Representative References
Race / Ethnicity	108 (80.0%)	Systemic Racism & Institutional Bias	Bidirectional / Negative	Quinn (2024); De Ornelas (2025)
Socioeconomic Status	107 (79.3%)	Market Dynamics & Labor Precarity	Bidirectional / Negative	Hamad (2025); Parra Moreno (2025)
Place of Residence	93 (68.9%)	Digital & Physical Infrastructure	Bidirectional / Negative	Adamkiewicz (2011); Noaeen (2025)
Gender & Sex	51 (37.8%)	Patriarchy & Institutional Sexism	Negative / Bidirectional	Bailey (2022); Dove-Medows (2025)
Sexual Orientation¹	16 (11.9%)	Structural Stigma & Social Norms	Bidirectional / Negative	Turan (2017); Balaji (2012)
Digital & Commercial²	13 (9.6%)	Data Sovereignty & Algorithmic Bias	Bidirectional / Negative	Egede (2022); Morey (2024)

¹ Included as a representative axis of structural discrimination and stigma-driven inequity

² Emerging dimension does not present in the original PROGRESS-Plus framework

Note: For the full distribution of all stratification variables (including Age, Education, and Disability), see Supplementary File 6

#### Question 3: Methodological strategies and theoretical FTM

The methodological landscape was characterized by a predominance of observational designs and a shift toward complex modeling aimed at isolating structural effects from intermediate variables.

##### Methodological strategies

There was a wide range of approaches for identifying health equity drivers. Primary quantitative designs represented the largest proportion of the evidence analyzed (*n* = 45; 33.3%), followed closely by secondary studies (*n* = 41; 30.4%). To isolate the effects of structural engines from confounding variables, multivariate analysis and other adjusted models were identified as the primary technical tools, being applied in a total of 46 studies (45 primary quantitative studies and 1 meta-analysis).

Within the primary quantitative studies (*n* = 45), cross-sectional designs predominated (*n* = 39), followed by retrospective cohorts and longitudinal analyses. Secondary studies (*n* = 41) consisted of systematic reviews, scoping reviews, and narrative syntheses. Primary qualitative designs accounted for 14.8% (*n* = 20), while mixed methods and case studies totaled 7.4% (*n* = 10). Finally, 14.1% of the evidence (*n* = 19) corresponded to other types of documents, including evidence-informed perspectives, institutional statements, and technical reports (Table 5).

This methodological diversity reflects the dual nature of drivers: while qualitative and conceptual analyses define their “meaning” and “social mechanism,” quantitative modeling provides the “magnitude” and “directionality” of their effect on health equity. More details in Supplementary File 5.

**Table 5:** Methodological strategies for identifying health equity drivers (*n* = 135)

Design category (*n*; %)	Methodological approach & analysis	Drivers identified (Examples)	Key references
Primary Quantitative (*n* = 45; 33.3%)	Multivariate Analysis* (*n* = 45)	Structural racism, housing quality, and chemical exposures.	Adamkiewicz (2011); Agarwal (2020); Barber (2018); Lewis (2021)
Secondary Studies (*n* = 41; 30.4%)	Meta-analysis* (*n* = 1); Narrative/Scoping synthesis (*n* = 40)	Structural racism, immigrant-focused policies, and cancer survival.	Klugman (2019); Di Petrillo (2024); Loehrer (2023)
Primary Qualitative (*n* = 20; 14.8%)	Interpretive, Content, & Thematic Analysis.	Institutional mistrust and lived experiences of exclusion.	Alkureishi (2021); Balaji (2012); Martinez-Leal (2023)
Mixed Methods / Case Studies (*n* = 10; 7.4%)	Policy Analysis & Comparative Case Studies.	Political leadership, access to legal representation.	Huicho (2020); Socal (2020); Kegler (2023)
Other types (*n* = 19; 14.1%)	Conceptual Analysis & Expert Consensus.	Political and digital drivers; ethical frameworks for action.	Coates (2020); PAHO (2019); Saigui (2021)

*Multivariate analysis and other adjusted models (Total *n* = 46 across categories)

Primary Quantitative breakdown: Cross-sectional (*n* = 39), Cohorts/Experimental (*n* = 6)

Secondary Studies: Includes Systematic, Scoping, and Narrative Reviews

Other Types: Includes Perspectives, Statements, and Institutional Reports

##### Theoretical and conceptual FTM

The theoretical landscape in the Americas demonstrates a significant transition in how health inequities are conceptualized. This scoping review identified that 91.1% (*n* = 123) of the analyzed explicitly applied conceptual frameworks, theories, or models (FTM). This marked increase reflects a contemporary shift from documenting social conditions to analyzing the systemic engines of disparity. This high level of theoretical density could be attributed to two primary factors:

###### Temporal Window (2020–2026)

Over 80% of the regional identified sample was published after 2020, a period characterized by a qualitative leap toward critical theories –such as Structural Racism and Digital Justice– as researchers moved beyond descriptive epidemiology following the COVID-19 pandemic.

###### Evidence Format

A significant proportion of the sample comprises reviews and position papers (Scoping, Systematic, and Critical Reviews), which inherently require operationalizing conceptual frameworks to synthesize evidence.

Notably, 25 authors (18.5% of the total sample) developed new frameworks (adapted or not) or strategic roadmaps to address contemporary gaps, including Structural Colonialism, Digital Determinants, and Algorithmic Justice. Table 6 categorizes these theoretical approaches into four dominant clusters, highlighting the shift toward models that prioritize the mechanisms of structural impact over individual-level variables.

**Table 6:** Theoretical and conceptual FTM applied to drivers (*n* = 124)

Framework category	Core theoretical bases	Key characteristics	Key references
Foundational & Socio-Ecological	Socio-Ecological Model; Fundamental Cause Theory; Life Course Perspective.	Analyzes multi-layered interactions between individual and environmental factors to explain the persistence of health disparities.	Agarwal (2020); Chandra (2016); Brown TH (2022); Campbell (2021)
Structural & Systemic Power	Structural Racism; Structural Violence; Settler Colonialism.	Examines macro-level systems and historical segregation. Investigates how institutional “engines” distribute health risks.	Bailey (2022); Gompers (2025); Correll-King (2025); Jernigan (2025)
Intersectionality & Embodiment	Intersectionality Theory; Weathering Hypothesis; Minority Stress; Biopsychosocial Models.	Explores overlapping social identities and the biological “weathering” caused by chronic exposure to systemic discrimination.	Thorpe (2025); Turan (2017); Menza (2025); Quinn (2024)
Emerging & Critical Frontiers	Digital Equity (SEED); Algorithmic Justice; Political Economy; Advocacy-Informed Research.	Focuses on data democratization, digital/commercial power dynamics, and the role of advocacy in bridging outcomes.	Bhavsar (2024); Credit (2020); Foryciarz (2025); Serchen (2021); Young (2025); Wainstein (2025)

#### Question 4: Research gaps and thematic distribution in the Americas

Research gaps and thematic distribution in the Americas evidence were concentrated in the 2021–2025 period, yet the distribution of topics reveals significant lacunae. Of the 135 documents analyzed in the Americas, 77.8% (*n* = 105) allowed for the identification of research gaps or the need for future investigation. These gaps represent areas where the structural engines of equity remain underexplored (Table 7). Data indicates a transition from documenting the existence of disparities toward a primary void in implementation strategies and the accountability of macro-level political and digital drivers.

**Table 7:** Synthesized map of identified and interpreted research gaps (*n* = 105)

Domain cluster	Primary gaps identified	Key references
I. Life Course & Aging	Absence of data on biological aging (Geroscience) linked to structural stress; lack of longitudinal trajectories for older adults.	Nielsen (2024); Thorpe (2025); Brown TH (2022)
II. Digital & Algorithmic Governance	Void in understanding algorithmic fairness; need for “Data Democratization” for Pacific Islanders and Indigenous groups.	Bhavsar (2024); Mourali (2025); Morey (2024)
III. Migration & Displacement	Lack of evidence on “Ontological Insecurity” and the health impact of restrictive immigration policies in LAC.	Parra Moreno (2025); Philbin (2018)
IV. Violence & Carceral Systems	Significant “silence” on the health impact of incarceration and systemic police violence as drivers of infection (MRSA, HIV).	Steinberg (2025); Menza (2025); Friedman (2021)
V. Political Economy & Advocacy	Under-reporting of political determinants and the role of professional advocacy training in bridging the equity gap.	Savage (2024); Hamad (2025); Serchen (2021)

## Discussion

This scoping review did not aim to answer classic health research questions focused on specific risk factors or clinical interventions. Instead, it sought to account for the evolving meanings and operational uses of the term “driver”, a word increasingly prevalent in global health forums yet often used with conceptual ambiguity. By examining 286 documents, of which 135 correspond to the regional sample of the Americas, we identified a discursive refinement where the traditional framework of “determinants” (SDoH) is being complemented by a more explicitly operational and “interventionist” vocabulary of “drivers” (SDEH: Social Drivers of Equity in Health). This shift does not imply that SDoH are conceptually static; rather, it suggests that the “driver” terminology is increasingly favored to isolate and emphasize the specific levers and policy engines required to disrupt established patterns of inequity.

### The conceptual refinement: From status to actionable forces

A primary finding was the lack of a universal definition for “drivers of health equity.” However, the evidence revealed a clear geographical and functional specialization. Global definitions emphasized macro-structural mechanisms and commercial determinants [38, 39], while Americas-based literature prioritized transformative capacity and stakeholder engagement [21, 40].

The use of “Synonymy” (34.1%) suggested that for many authors, the term “driver” acts as an operational catalyst that accentuates the possibility of change. Rather than replacing the structural and dynamic nature of SDoH, the “driver” terminology appears to isolate the specific, active forces that produce and sustain social environments. As suggested by Serchen and Mercado [40, 41], the term provides a more proactive vocabulary. By framing issues like structural racism or digital redlining as “drivers,” the literature concentrates the focus on the underlying mechanisms –such as exclusionary urban policy– that govern the distribution of social conditions. This distinction is vital: it identifies specific mechanisms for systemic recalibration, providing a functional link between monitoring and action.

While SDoH often describes static environments, we interpreted “drivers” as the active, systemic forces that create and sustain those environments. This shifts the focus from the consequences of inequity (e.g., poor housing) to the mechanisms of inequity (e.g., exclusionary urban policy), identified as specific “drivers” of change.

### A taxonomy of operational forces: Upstream, midstream, and downstream

To address conceptual ambiguity and avoid synonymy with traditional SDoH, we define a “social driver of health equity” (SDEH) as an active, structural, or systemic force (political, commercial, legal, or institutional) that exerts pressure to distribute power, resources, and social positions, thereby actively generating, sustaining, or modifying health inequities [40, 41].

While a “determinant” (SDoH) characterizes the resulting social circumstance or environmental *status quo* (e.g., housing condition, food insecurity), a “driver” isolates the dynamic mechanism of power that puts those determinants in motion [42].

Through this kinetic lens, we distinguish three operational dimensions:

- Kinetic power: The macro-structural capacity of political and economic institutions to dictate resource allocation and enforce systemic policies (e.g., exclusionary zoning laws, tax structures, or the carceral state) [43–45].
- Kinetic force: The active momentum, pressure, or directional vector exerted by these institutions on populations, pushing individuals into stratified social positions (e.g., predatory subprime lending, algorithmic redlining, or commercial lobbying) [46–48].
- Kinetic mechanism: The biological, psychological, or institutional pathway through which this force is operationalized and embodied (e.g., physiological weathering, chronic allostatic load, provider implicit bias, or administrative burdens) [36, 37].

In real-world policy contexts, understanding drivers through this kinetic lens shifts public health practice from mitigating passive downstream deficits (such as delivering emergency food aid) to disrupting the active upstream engines of extraction and power that mandate those déficits [42]. Based on these action-oriented interpretations, we propose an operational taxonomy across three levels of influence:

1. Upstream Drivers (Structural Engines): Focus on macro-level systems-law, policy, and economy –that function as Marmot’s “causes of the causes” [45, 49, 50]. In this context, “driver” is used to emphasize the kinetic power of these systems to dictate resource distribution. They are the primary power engines identified in the region, including historical legacies like the carceral state.
2. Midstream Drivers (Systemic Mechanisms): These act as the “connective tissue” between structure and outcomes, including institutional betrayal and the “weathering” effect [51, 52]. Rather than mere intermediary states, these are viewed as active processes of embodiment, such as the biological internalization of chronic stress, as well as systemic filters of implicit bias, commercial power dynamics, and barriers to institutional navigation. In this sense, they function as the kinetic mechanisms through which structural inequities are translated into individual and collective health trajectories.
3. Downstream Drivers (Operational Targets): This category reflects studies where traditional social determinants are labeled as “drivers” to emphasize their modifiability. Unlike Upstream Drivers, which function as the macro-architectural “engines” of inequality, Downstream Drivers are treated as proximal entry points. By applying the “driver” nomenclature to factors such as housing or labor stability, the literature shifts these from static conditions to specific foci for policy realignment [40, 53, 54].

This hierarchy aligns with the SDoH and Social Needs Model [42], which distinguishes between community-level conditions and individual resource needs. While frameworks such as the NIMHD Research Framework [55] often categorize these as “midstream” factors, this review adopts the term “driver” to emphasize their dynamic and contingent nature. As noted by Castrucci [42], addressing individual social needs is essential but insufficient without concurrent action on the structural drivers that necessitate those needs.

### Refining the legacy: From Marmot’s roots to structural engines

A core contribution of this review is to disentangle the conceptual overlap between “determinants” (SDoH) and “drivers” (SDEH), a distinction that is critical for the future of health equity research. While 34.1% of the literature still uses these terms synonymously, our analysis reveals an emerging conceptual transition. Traditionally, as established in the foundational work of Marmot, SDoH have been treated as “landscape” or “status” variables that describe the conditions in which people live, such as housing quality or employment status. In contrast, the “driver” terminology, as operationalized in the contemporary Americas-based literature, functions as an operational descriptor that identifies the specific forces putting those conditions in motion.

This evolution does not replace Marmot’s foundational framework; rather, it operationalizes his “causes of the causes” for the current policy landscape by transforming classic SDoH into functional, strategic priorities. Under this lens, macro-structural determinants like economic policy are no longer viewed merely as contexts but as the primary engines of resource distribution, while traditional conditions such as housing and labor stability are re-labeled as drivers to emphasize their mandate for structural remediation. In this sense, authors like Serchen [40] and Hamad [53] shift the focus from the determinant as a static description of a setting to the driver as a proximal entry point for change. The difference we mark here is fundamental: while a “determinant” describes a situation, a “driver” identifies a force that must be dismantled or mobilized. Therefore, the shift toward “SDEH” in the Americas represents a definitive move toward structural competency, where identifying a health inequity implies a formal decision and the agency to intervene in it, reinforcing the ethical judgment originally proposed by Mujica and Moreno [56].

### The role of the COVID-19 syndemic and shifting taxonomies

The crisis functioned as a catalyst for driver-based research, demonstrating that the pandemic was not an isolated event but a “syndemic” where social and biological factors interacted to multiply vulnerability [57]. Racism emerged as the most mentioned driver, especially in the US [18, 27, 58].

This period marked a critical shift in taxonomy; as proposed by Brakefield et al. [59], the pandemic necessitated redefining the SDoH framework to characterize barriers not as linear categories, but as dynamic causal pathways. The high frequency of racism as a primary driver illustrates how structural forces function as kinetic mechanisms of inequity, capturing the intersecting pressures that traditional, static models struggle to isolate. This shift toward SDEH during the crisis underscores the need for a vocabulary that reflects the non-linear and modifiable nature of these systemic forces.

### Methodological and theoretical maturity: PROGRESS-plus and beyond

The methodological landscape reveals a shift toward analytical maturity, evidenced by the predominance of multivariate analysis (*n* = 47) and hierarchical modeling, allowing researchers to disentangle macro-structural engines from individual determinants. We identified a theoretical maturation where 18.5% of America-based records (*n* = 25) moved beyond established theories to propose original frameworks.

This theoretical evolution is operationalized through the systematic use of the PROGRESS-PLUS framework, which functions as more than a classification tool. As shown in our mapping, PROGRESS-PLUS allows for the identification of how macro-structural engines intersect with specific social identities, revealing that drivers, such as algorithmic bias or structural racism [47, 48], are not static traits but kinetic forces. By cross-referencing these variables, we move from a flat description toward a multidimensional understanding of how power and structural neglect are distributed. Specialized frameworks like Algorithmic Justice [31] and Advocacy-Informed Research [60] reflect a need to capture the “missing links” of power.

### The paradox of regional knowledge and silent drivers

Despite this maturity, an “inequity in research” persists, approximately 80% of the evidence is concentrated in the US. In this context, while some US studies have begun to highlight underappreciated drivers such as primary language [61], other critical concepts like “digital discrimination” [32, 47] or the “prison state” [45] remain virtually invisible in the literature on LAC. This “silence” on carceral and digital drivers underscores a regional knowledge gap that persists even when traditional social determinants are analyzed.

While the theoretical framework of the political determinants of health [43] highlights power, the literature has yet to fully integrate climate justice and the intersection of political financing/lobbying as core drivers [10, 62]. However, the identification of positive drivers- social protection, labor stability, and data democratization- emerges as modifiable “levers” capable of neutralizing the engines of structural neglect [32, 53]. More than 15 years after the WHO Commission, a profound gap remains in “how to” intervene, necessitating a transition to implementation science to examine the political context of policy formulation [7].

### Limitations

Several limitations must be acknowledged to contextualize the reach of this study. First, the inherent complexity of addressing four distinct research questions within a single scoping review necessitated an expansive and inclusive search strategy. While this breadth was essential to capture the full spectrum of terminology and evolving concepts surrounding “drivers,” the massive volume of studies required a high-level synthesis that may have overlooked specific niche theoretical nuances.

Second, the categorization process could have introduced subjectivity. The interpretation of “drivers,” the application of the PROGRESS-Plus framework, and the assessment of intersectionality and directionality are non-linear tasks. Although all data were rigorously checked for precision and completeness, the inconsistent use of terms like “driver,” “determinant,” and “factor” in the sources often blurred the lines of directionality, potentially introducing minor classification errors.

Third, although the predominance of English-language publications allowed for a robust analysis of global trends, it also highlights a significant language and regional bias. Publications in Spanish and Portuguese were notably scarce in the final sample, and many were excluded during the screening process for not meeting the specific inclusion criteria or lacking sufficient conceptual depth regarding “drivers”. This scarcity may also reflect a lower frequency of the term “drivers”, or its counterparts “*motores*” in Spanish and “*forças motoras*” in Portuguese, within the LAC academic discourse, where traditional frameworks may prioritize alternative nomenclature.

Finally, while every effort was made to be exhaustive, the rapid evolution of this field means that some very recent or non-indexed institutional reports might have escaped our search.

## Conclusions

This review indicates that while a single, universal definition for “health equity drivers” has yet to emerge, the field reflects a set of complexes, evolving paradigms. Rather than replacing established frameworks, the conceptual transition toward Social Drivers of Equity in Health (SDEH) in the Americas represents a move toward structural competency, complementing the static description of social environments with the identification of systemic forces that require active modification. The proposed “hierarchy of drivers” (Upstream-Midstream-Downstream) serves as a pragmatic lens to identify precise levers of power, such as legal reforms, market dynamics, and data democratization, that put social determinants in motion toward justice. This distinction is functional: while a determinant characterizes the setting, the driver isolates the kinetic force to be dismantled or mobilized. Ultimately, addressing health inequity in contemporary literature transcends a measurement exercise; it implies the agency and formal decision to intervene in the mechanisms that produce it.

## Supplementary Information

Below is the link to the electronic supplementary material.

Supplementary Material 1 S1: Search strategies and results.

Supplementary Material 2 S2: Included records.

Supplementary Material 3 S3: Excluded records, with reasons.

Supplementary Material 4 S4: Drivers’ definitions.

Supplementary Material 5 S5: Details of drivers, effect direction, methodology, frameworks, and research gaps.

Supplementary Material 6 S6: PROGRESS-PLUS and Thematic Matrix.

## Data Availability

No datasets were generated or analysed during the current study.

## Declarations

### AI and AI-assisted technologies in the writing process

During the data synthesis phase, Gemini (Google) was utilized to assist in the refinement of drivercategorizations and the clustering of theoretical and conceptual frameworks. The authors also usedthe tool to improve the wording of the final manuscript. Following the use of this technology, theauthors critically reviewed, verified, and edited all outputs to ensure accuracy and scientific rigor,assuming full responsibility for the integrity of the final publication. Representative examples ofthe prompts used are documented in Supplementary File 5.

## Abbreviations

CIHR: Canadian Institutes of Health Research
COVID-19: Coronavirus Disease of 2019
EURO: European Region (WHO)
FTM: frameworks, theories, or models
HESR: Health Equity Status Report
HIV: Human Immunodeficiency Virus
JBI-PPC: Joanna Briggs Institute’s Population, Concept, Context framework
LAC: Latin America and the Caribbean
MRSA: Methicillin-resistant Staphylococcus aureus
NIH: National Institutes of Health (US)
NIMHD: National Institute on Minority Health and Health Disparities (US)
OSF: Open Science Framework
PAHO: Pan American Health Organization
PRISMA-ScR: Preferred Reporting Items for Systematic Reviews and Meta-Analyses: Extension for Scoping Reviews
PROGRESS-Plus: Place of residence, Race/ethnicity, Occupation, Gender, Religion, Education, Social capital, Socioeconomic status, Age, Disability, and Sexual orientation
RWJF: Robert Wood Johnson Foundation
SDEH: Social Drivers of Equity in Health
SDoH: Social Determinants of Health
SEED: Shared equity in economic development
SES: Socio-economic status
US: United States of America
WHO: World Health Organization
