# Supplementary material for: Drivers of health equity in the Americas: a scoping review
Source: Int J Equity Health. 2026 Sep 16;25:255. doi: 10.1186/s12939-026-03031-6 (PMC13695892; doi:10.1186/s12939-026-03031-6)
Supplement: Supplementary file 1 [file 12939_2026_3031_MOESM1_ESM.docx]

**Drivers of Health Equity in the Americas**

**Supplementary File 1. Search syntax and terms used for specific database searches**

**Medline (PubMed)**

#1 driver* [tw]

#2 ("Health Equity"[Mesh] OR "Health Inequities"[Mesh] OR equit* OR inequit* OR disparit* OR inequalit*)

#3 ("Social Determinants of Health"[Mesh] OR "Epidemiologic Factors"[Mesh] OR PROGRESS* OR determinant*[Tw])

#1 #1 AND #2 AND #3

**Embase**

#1 driver$

#2 'health inequities'

#3 'health equity'

#4 equit$

#5 inequit$

#6 disparit$

#7 inequalit$

#8 'social determinants of health'

#9 'epidemiologic factors'

#10 progress$

#11 determinant$

#12 #1 AND (#2 OR #3 OR #4 OR #5 OR #6 OR #7) AND (#8 OR #9 OR #10 OR #11)

**Lilacs**

(mh:"Equidad en Salud" OR mh:"Inequidades en Salud" OR equit$ OR equid$ OR inequit$ OR inequid$ OR disparidad$ OR desigual$ OR PROGRESS$)  AND (impuls$ OR driver$)

**Epistemonikos**

(title:((equit* OR inequit* OR disparit* OR inequalit* OR PROGRESS*)) OR abstract:((equit* OR inequit* OR disparit* OR inequalit* OR PROGRESS*))) AND (title:(driver*) OR abstract:(driver*)

**Others**

Combinations of 'driver', 'health AND equity', and 'determinants' as free-text terms

Literature searches were performed up to January 30, 2026

| **Database and coverage** | **Results** |
| --- | --- |
| Embase Classic+Embase | 1504 |
| Ovid MEDLINE | 1799 |
| LILACS | 1104 |
| Epistemonikos | 3716 |
| CINAHL | 711 |
| **Total** | **8834** |
