# Supplementary material for: Drivers of health equity in the Americas: a scoping review
Source: Int J Equity Health. 2026 Sep 16;25:255. doi: 10.1186/s12939-026-03031-6 (PMC13695892; doi:10.1186/s12939-026-03031-6)
Supplement: Supplementary file 2 [file 12939_2026_3031_MOESM2_ESM.docx]

**Drivers of Health Equity in the Americas**

**Supplementary file 2. Included records**

1. Abba-Aji M, Stuckler D, Galea S, McKee M. Ethnic/racial minorities’ and migrants’ access to COVID-19 vaccines: A systematic review of barriers and facilitators. Journal of migration and health. 2022;5:100086.
2. Abubakar I, Gram L, Lasoye S, Achiume ET, Becares L, Bola GK, et al. Confronting the consequences of racism, xenophobia, and discrimination on health and health-care systems. The Lancet. 2022;400(10368):2137–46.
3. Adamkiewicz G, Zota AR, Fabian MP, Chahine T, Julien R, Spengler JD, et al. Moving environmental justice indoors: understanding structural influences on residential exposure patterns in low-income communities. American journal of public health. 2011;101 Suppl 1(Suppl 1):S238–45.
4. Adams AM, Rabbani A, Ahmed S, Mahmood SS, Al-Sabir A, Rashid SF, et al. Explaining equity gains in child survival in Bangladesh: scale, speed, and selectivity in health and development. Lancet (London, England). 2013;382(9909):2027–37.
5. Adepoju VA, Odueniyi C, Abdulrahim A, Adnani QES. Respectful maternity care and intersectional identities in low- and middle-income countries: a systematic review. BMC pregnancy and childbirth. 2025.
6. Agarwal S, Kanapka LG, Raymond JK, Walker A, Gerard-Gonzalez A, Kruger D, et al. Racial-Ethnic Inequity in Young Adults with Type 1 Diabetes. The Journal of Clinical Endocrinology and Metabolism. 2020;105(8):e2960–e9.
7. Agha S, Carton TW. Determinants of institutional delivery in rural Jhang, Pakistan. Int J Equity Health. 2011;10.
8. Aguiar R, Keil R, Wiktorowicz M. The urban political ecology of antimicrobial resistance: A critical lens on integrative governance. Social Science & Medicine. 2024;348:N.PAG–N.PAG.
9. Aguiar R, Ruckert A, Harris F, Boudreau LeBlanc A, Carmo LP, Davies-Venn M, et al. Equity in the governance of antimicrobial resistance surveillance: Global experts' perspectives. Social Science & Medicine. 2025;387:N.PAG–N.PAG.
10. Akseer N, Phillips DE. Drivers of success in global health outcomes: A content analysis of Exemplar studies. PLOS global public health. 2024;4(5):e0003000.
11. Akseer N, Vaivada T, Rothschild O, Ho K, Bhutta ZA. Understanding multifactorial drivers of child stunting reduction in Exemplar countries: a mixed-methods approach. The American journal of clinical nutrition. 2020;112(Suppl 2):792S–805S.
12. Alaba OA, Hongoro C, Thulare A, Lukwa AT. Drivers of socioeconomic inequalities of child hunger during COVID-19 in South Africa: evidence from NIDS-CRAM Waves 1-5. BMC Public Health. 2022;22(1):2092.
13. Alderton A, Henry N, Foster S, Badland H. Examining the relationship between urban liveability and gender-based violence: A systematic review. Health & place. 2020;64:102365.
14. Alkureishi MA, Choo Z-Y, Rahman A, Ho K, Benning-Shorb J, Lenti G, et al. Digitally Disconnected: Qualitative Study of Patient Perspectives on the Digital Divide and Potential Solutions. JMIR human factors. 2021;8(4):e33364.
15. Andermann A, Mott S, Mathew CM, Kendall C, Mendonca O, Harriott D, et al. Evidence-informed interventions and best practices for supporting women experiencing or at risk of homelessness: a scoping review with gender and equity analysis. Health promotion and chronic disease prevention in Canada : research, policy and practice. 2021;41(1):1–13.
16. Anderson M, Ljungqvist G, van Kessel R, Saint V, Mossialos E. The socioeconomic drivers and impacts of Antimicrobial Resistance: Implications for policy and research. 2024.
17. Auerbach JD, Parkhurst JO, Cáceres CF. Addressing social drivers of HIV/AIDS for the long-term response: conceptual and methodological considerations. Global public health. 2011;6(sup3):S293–S309.
18. Ayanore MA, Pavlova M, Groot W. Unmet reproductive health needs among women in some West African countries: a systematic review of outcome measures and determinants. Reproductive Health. 2016;13(1):5.
19. Badal K, Lee CM, Esserman LJ. Guiding principles for the responsible development of artificial intelligence tools for healthcare. Communications Medicine. 2023;3(1):47.
20. Bærøe K, Árnason V, Jansen M, Yamin AE, Ruano AL, Davis AP. Pandemic and Crisis Preparedness and Response: Conceptualizing Cultural, Social and Political Drivers of Trustworthiness and Collective Action. Public health ethics. 2025;18(2):phaf004.
21. Bailey HH, Janssen MF, Alladin FM, La Foucade A, Varela R, Moreno JA, et al. Evaluating Health Inequality in Five Caribbean Basin Countries Using EQ-5D-5L. Appl Health Econ Health Policy. 2022;20(6):857–66.
22. Bailey K, Avolio J, Lo L, Gajaria A, Mooney S, Greer K, et al. Social and Structural Drivers of Health and Transition to Adult Care. Pediatrics. 2023.
23. Baker P, Slater S, White M, Wood B, Contreras A, Corvalan C, et al. Towards unified global action on ultra-processed foods: understanding commercial determinants, countering corporate power, and mobilising a public health response. The Lancet. 2025.
24. Balaji AB, Oster AM, Viall AH, Heffelfinger JD, Mena LA, Toledo CA. Role flexing: how community, religion, and family shape the experiences of young black men who have sex with men. AIDS patient care and STDs. 2012;26(12):730–7.
25. Barber S, Diez Roux AV, Cardoso L, Santos S, Toste V, James S, et al. At the intersection of place, race, and health in Brazil: Residential segregation and cardio-metabolic risk factors in the Brazilian Longitudinal Study of Adult Health (ELSA-Brasil). Soc Sci Med. 2018;199:67–76.
26. Barber S, Hickson DA, Kawachi I, Subramanian SV, Earls F. Neighborhood Disadvantage and Cumulative Biological Risk Among a Socioeconomically Diverse Sample of African American Adults: An Examination in the Jackson Heart Study. Journal of racial and ethnic health disparities. 2016;3(3):444–56.
27. Barnes LL. Alzheimer disease in African American individuals: increased incidence or not enough data? Nat Rev Neurol. 2022;18(1):56–62.
28. Barros AJD, Wehrmeister FC, Ferreira LZ, Vidaletti LP, Hosseinpoor AR, Victora CG. Are the poorest poor being left behind? Estimating global inequalities in reproductive, maternal, newborn and child health. BMJ Global Health. 2020;5(1):e002229.
29. Bartel D, Coile A, Zou A, Martinez Valle A, Nyasulu HM, Brenzel L, et al. Exploring system drivers of gender inequity in development assistance for health and opportunities for action. Gates Open Research. 2023;6:114.
30. Bécares L, Cormack D, Harris R. Ethnic density and area deprivation: neighbourhood effects on Māori health and racial discrimination in Aotearoa/New Zealand. Social science & medicine (1982). 2013;88:76–82.
31. Bedingfield N, Lashewicz B, Fisher D, King-Shier K. Systems of support for foreign-born TB patients and their family members. Public health action. 2022;12(2):79–84.
32. Ben J, Cormack D, Harris R, Paradies Y. Racism and health service utilisation: A systematic review and meta-analysis. PloS one. 2017;12(12):e0189900.
33. Benfer EA, Vlahov D, Long MY, Walker-Wells E, Pottenger JL, Jr., Gonsalves G, et al. Eviction, Health Inequity, and the Spread of COVID-19: Housing Policy as a Primary Pandemic Mitigation Strategy. Journal of urban health : bulletin of the New York Academy of Medicine. 2021;98(1):1–12.
34. Bergen N, Cata-Preta BO, Schlotheuber A, Santos TM, Danovaro-Holliday MC, Mengistu T, et al. Economic-Related Inequalities in Zero-Dose Children: A Study of Non-Receipt of Diphtheria–Tetanus–Pertussis Immunization Using Household Health Survey Data from 89 Low- and Middle-Income Countries. Vaccines. 2022;10(4):633.
35. Bhavsar NA, Sperling J, Ruiz R, Mohottige D, Muhigaba P, Silberberg M, et al. SEED: A novel web-based data visualization platform to visualize, communicate, and explore social, environmental, and equity drivers of health. Journal of Clinical and Translational Science. 2024;8(1):e121.
36. Bisgaier J, Rhodes KV, Polsky D. Factors Associated with Increased Specialty Care Access in an Urban Area: The Roles of Local Workforce Capacity and Practice Location. Journal of health politics, policy and law. 2014;39(6):1173–83.
37. Black D, Bates G, Callway R, Pain K, Kirton-Darling E. Short-termism in urban development: The commercial determinants of planetary health. Earth system governance. 2024;22:None.
38. Boerma. Countdown to 2030: tracking progress towards universal coverage for reproductive, maternal, newborn, and child health. Lancet (London, England). 2018;391(10129):1538–48.
39. Bourey C, Bass J, Stephenson R. Women's Employment and Intimate Partner Violence: Understanding the Role of Individual and Community Structural Drivers in Low- and Middle-Income Countries. Journal of interpersonal violence. 2023;38(9-10):6480–99.
40. Bradley JE, Bhattacharjee P, Ramesh BM, Girish M, Das AK. Evaluation of stepping stones as a tool for changing knowledge, attitudes and behaviours associated with gender, relationships and HIV risk in Karnataka, India. BMC Public Health. 2011;11:496.
41. Brakefield WS, Ammar N, Olusanya OA, Shaban-Nejad A. An Urban Population Health Observatory System to Support COVID-19 Pandemic Preparedness, Response, and Management: Design and Development Study. JMIR public health and surveillance. 2021;7(6):e28269.
42. Brawner BM, Reason JL, Goodman BA, Schensul JJ, Guthrie B. Multilevel drivers of human immunodeficiency virus/acquired immune deficiency syndrome among Black Philadelphians: exploration using community ethnography and geographic information systems. Nursing research. 2015;64(2):100–10.
43. Brown LJ, Lowe H, Gibbs A, Smith C, Mannell J. High-Risk Contexts for Violence Against Women: Using Latent Class Analysis to Understand Structural and Contextual Drivers of Intimate Partner Violence at the National Level. Journal of interpersonal violence. 2023;38(1-2):NP1007–NP39.
44. Brown TH, Kamis C, Homan P. Empirical evidence on structural racism as a driver of racial inequities in COVID-19 mortality. Frontiers in public health. 2022;10:1007053.
45. Brubacher LJ, Peach L, Chen TT, Longboat S, Dodd W, Elliott SJ, et al. Climate change, biodiversity loss, and Indigenous Peoples' health and wellbeing: A systematic umbrella review. PLOS global public health. 2024;4(3):e0002995.
46. Burns JK. Poverty, inequality and a political economy of mental health. Epidemiol Psychiatr Sci. 2015;24(2):107–13.
47. Buse K, Tanaka S, Hawkes S. Healthy people and healthy profits? Elaborating a conceptual framework for governing the commercial determinants of non-communicable diseases and identifying options for reducing risk exposure. Globalization and health. 2017;13(1):34.
48. Butts SA, Parmley LE, Alcaide ML, Rodriguez VJ, Kayukwa A, Chitalu N, et al. Let us fight and support one another: adolescent girls and young women on contributors and solutions to HIV risk in Zambia. International journal of women's health. 2017;9:727–37.
49. Bwirire D, Crutzen R, Ntabe Namegabe E, Letschert R, de Vries N. Health inequalities in post-conflict settings: A systematic review. PloS one. 2022;17(3):e0265038.
50. Caffe S, Plesons M, Camacho AV, Brumana L, Abdool SN, Huaynoca S, et al. Looking back and moving forward: can we accelerate progress on adolescent pregnancy in the Americas? Reproductive health. 2017;14(1):83.
51. Campbell JA, Yan A, Walker RE, Weinhardt L, Wang Y, Walker RJ, et al. Relative Contribution of Individual, Community, and Health System Factors on Glycemic Control Among Inner-City African Americans with Type 2 Diabetes. Journal of racial and ethnic health disparities. 2021;8(2):402–14.
52. Cené CW, Viswanathan M, Fichtenberg CM, Sathe NA, Kennedy SM, Gottlieb LM, et al. Racial Health Equity and Social Needs Interventions: A Review of a Scoping Review. JAMA network open. 2023;6(1):e2250654.
53. Cerny C, Cheeseman H, Jenner K, Severi K. Tobacco as a model: Developing a commercial determinants of health approach to the three big killers. Tobacco Induced Diseases. 2025;23(Supplement 1):286–7.
54. Chalasani R, Krishnamurthy S, Suda KJ, Newman TV, Delaney SW, Essien UR. Pursuing Pharmacoequity: Determinants, Drivers, and Pathways to Progress. Journal of health politics, policy and law. 2022;47(6):709–29.
55. Chambers T, Mizdrak A, Herbert S, Jones A. Interventions to reduce alcohol’s harms to health: a modelling study. Wellington: Te Whatu Ora–Health New Zealand. 2023.
56. Chandra A, Miller CE, Acosta JD, Weilant S, Trujillo M, Plough A. Drivers Of Health As A Shared Value: Mindset, Expectations, Sense Of Community, And Civic Engagement. Health Aff (Millwood). 2016;35(11):1959–63.
57. Chirwa GC, Mazalale J, Likupe G, Nkhoma D, Chiwaula L, Chintsanya J. An evolution of socioeconomic related inequality in teenage pregnancy and childbearing in Malawi. PloS one. 2019;14(11).
58. Christian B, Blinkhorn A. A review of dental caries in Australian Aboriginal children: the health inequalities perspective. 2012.
59. Cislaghi B, Weber AM, Gupta GR, Darmstadt GL. Gender equality and global health: intersecting political challenges. Journal of global health. 2020;10(1):010701.
60. Cleveland Manchanda EC, Marsh RH, Osuagwu C, Decopain Michel J, Dugas JN, Wilson M, et al. Heart Failure Admission Service Triage (H-FAST) Study: Racialized Differences in Perceived Patient Self-Advocacy as a Driver of Admission Inequities. Cureus. 2021;13(2):e13381.
61. Coates A, Castro A, Marmot M, Mújica OJ, Eijkemans G, Victora CG. Sociedades justas: una nueva visión de la equidad en la salud en la Región de las Américas después de la COVID-19. Revista Panamericana de Salud Pública. 2021;45.
62. Cole HVS, Anguelovski I, Triguero-Mas M, Mehdipanah R, Arcaya M. Promoting Health Equity Through Preventing or Mitigating the Effects of Gentrification: A Theoretical and Methodological Guide. Annual Review of Public Health. 2023;44(1):193–211.
63. Collin LJ, Gaglioti AH, Beyer KM, Zhou Y, Moore MA, Nash R, et al. Neighborhood-Level Redlining and Lending Bias Are Associated with Breast Cancer Mortality in a Large and Diverse Metropolitan Area. Cancer epidemiology, biomarkers & prevention : a publication of the American Association for Cancer Research, cosponsored by the American Society of Preventive Oncology. 2021;30(1):53–60.
64. Colson KE, Dwyer-Lindgren L, Achoki T, Fullman N, Schneider M, Mulenga P, et al. Benchmarking health system performance across districts in Zambia: a systematic analysis of levels and trends in key maternal and child health interventions from 1990 to 2010. BMC medicine. 2015;13:69.
65. Conway K, Akseer N, Subedi RK, Brar S, Bhattarai B, Dhungana RR, et al. Drivers of stunting reduction in Nepal: A country case study. American Journal of Clinical Nutrition. 2020;112:844S–59S.
66. Correll-King WM, Ubong IA, Monro D, Scott K, Strunk SN, Stephenson J, et al. Triggered: Qualitatively exploring structural and social drivers of firearm violence exposure among LGBTQ+ young adults of color in Detroit. Social Science and Medicine. 2025;384:118524.
67. Craig GM, Daftary A, Engel N, O’Driscoll S, Ioannaki A. Tuberculosis stigma as a social determinant of health: a systematic mapping review of research in low incidence countries. International Journal of Infectious Diseases. 2017;56:90–100.
68. Credit K. Neighbourhood inequity: Exploring the factors underlying racial and ethnic disparities in COVID-19 testing and infection rates using ZIP code data in Chicago and New York. Regional Science Policy & Practice. 2020;12(6):1249–71.
69. Crocetti AC, Beau C, Yorta TW, Fiona M, Yin P, Backholer K, et al. ‘A recipe for cultural disaster!’– a case study of Woolworths Group’s proposal to build an alcohol megastore in Darwin, Northern Territory. Research Square; 2023.
70. Cros M, Cavagnero E, Alfred JP, Sjoblom M, Collin N, Mathurin T. Equitable realization of the right to health in Haiti: how household data inform health seeking behavior and financial risk protection. Int J Equity Health. 2019;18(1):77.
71. Cuadros DF, Gutierrez JD, Moreno CM, Escobar S, Miller D, Musuka G, et al. Impact of healthcare capacity disparities on the COVID-19 vaccination coverage in the United States. medRxiv. 2022((Cuadros D.F., diego.cuadros@uc.edu; Escobar S.) Digital Epidemiology Laboratory, Digital Futures, University of Cincinnati, Ohio, Cincinnati, United States).
72. d'Elia A, Gabbay M, Rodgers S, Kierans C, Jones E, Durrani I, et al. Artificial intelligence and health inequities in primary care: a systematic scoping review and framework. Fam Med Community Health. 2022;10(Suppl 1).
73. Dalsania AK, Fastiggi MJ, Kahlam A, Shah R, Patel K, Shiau S, et al. The Relationship Between Social Determinants of Health and Racial Disparities in COVID-19 Mortality. Journal of racial and ethnic health disparities. 2022;9(1):288–95.
74. Darling-Hammond S, Gutierrez A, Le C, Atangana CB, Thomas Tobin C. Rooted in identity: racial centrality buffers the effects of chronic stress on suicide outcomes among Black Americans. Ethnicity & health. 2025;30(8):845–62.
75. Davis K, Limato R, Monga M, Egid B, Paul S, Okioma S, et al. Antimicrobial resistance, equity and justice in low- and middle-income countries: an intersectional critical interpretive synthesis. Nature Communications. 2025;16(1):9078.
76. De Ornelas M, Warner M, Jones L, Hooper S, Garrett SB. Legal Reform to Address Key Drivers of Racial Inequities in Maternal Health: A Multi-method Analysis of California Laws & Regulations from 2019 to 2023. Maternal and Child Health Journal. 2025:1–11.
77. Derose KP, Payán DD, Fulcar MA, Terrero S, Acevedo R, Farías H, et al. Factors contributing to food insecurity among women living with HIV in the Dominican Republic: A qualitative study. PloS one. 2017;12(7):e0181568.
78. Dharma C, Bondy SJ, Sikstrom L, Muirhead PS, Zaheer J, Maslej MM. Examining Systemic and Interpersonal Bias in Violence Risk Assessments of Patients in Acute Psychiatric Care. Psychiatric services (Washington, DC). 2025;76(4):326–35.
79. Diderichsen F, Dahlgren G, Whitehead M. Beyond 'commercial determinants': shining a light on privatization and political drivers of health inequalities. European Journal of Public Health. 2021;31(4):672–3.
80. DiPetrillo B, Adkins-Jackson PB, Yearby R, Dixon C, Pigott TD, Petteway RJ, et al. Characteristics of interventions that address racism in the United States and opportunities to integrate equity principles: a scoping review. Systematic Reviews. 2024;13(1):266.
81. Disney G, Teng A, Atkinson J, Wilson N, Blakely T. Changing ethnic inequalities in mortality in New Zealand over 30 years: linked cohort studies with 68.9 million person-years of follow-up. Population health metrics. 2017;15(1):15.
82. Ditah CM, Rahman E, Agbor VN, Foryoung JB, Shahzad M, Amgai B, et al. Disparities and drivers of early age at diagnosis of congestive heart failure in the USA. Int J Cardiol. 2019;293:143–7.
83. Donneyong MM, Fischer MA, Langston MA, Joseph JJ, Juarez PD, Zhang P, et al. Examining the drivers of racial/ethnic disparities in non-adherence to antihypertensive medications and mortality due to heart disease and stroke: A county-level analysis. International Journal of Environmental Research and Public Health. 2021;18(23).
84. Douglas JA, Subica AM. COVID-19 treatment resource disparities and social disadvantage in New York City. Preventive medicine. 2020;141:106282.
85. Dove-Medows E, Walsh A, Corwin C. Talking about Risk and Racism with Midwives: The TERM Study. Journal of Midwifery and Women's Health. 2025;70(6):850.
86. Dubowitz T, Orleans T, Nelson C, May LW, Sloan JC, Chandra A. Creating healthier, more equitable communities by improving governance and policy. Health Affairs. 2016;35(11):1970–5.
87. Duchowny KA, Hicken MT, Cawthon PM, Glymour MM, Clarke P. Life course trauma and muscle weakness in older adults by gender and race/ethnicity: Results from the U.S. health and Retirement Study. SSM Popul Health. 2020;11:100587.
88. Dwyer-Lindgren L, Bertozzi-Villa A, Stubbs RW, Morozoff C, Mackenbach JP, van Lenthe FJ, et al. Inequalities in Life Expectancy Among US Counties, 1980 to 2014: Temporal Trends and Key Drivers. JAMA internal medicine. 2017;177(7):1003–11.
89. Egede LE, Ozieh MN, Campbell JA, Williams JS, Walker RJ. Cross-Sector Collaborations Between Health Care Systems and Community Partners That Target Health Equity/Disparities in Diabetes Care. Diabetes Spectr. 2022;35(3):313–9.
90. Eke SM, Cua A. Invisible Engines of Resistance: How Global Inequities Drive Antimicrobial Failure. Antibiotics (Basel, Switzerland). 2025;14(7).
91. El-Bassel N, Shaw SA, Dasgupta A, Strathdee SA. People who inject drugs in intimate relationships: it takes two to combat HIV. Current HIV/AIDS reports. 2014;11(1):45–51.
92. El-Saharty S, Chowdhury S, Ohno N, Sarker I. Improving maternal and reproductive health in South Asia: Drivers and enablers: World Bank Publications; 2016.
93. English D, Carter JA, Boone CA, Forbes N, Bowleg L, Malebranche DJ, et al. Intersecting Structural Oppression and Black Sexual Minority Men's Health. American Journal of Preventive Medicine. 2021;60(6):781–91.
94. Estime SR, Bradley AS, Williams B. Disparities Among Trauma Patients and Interventions to Address Equitable Health Outcomes. Current Trauma Reports. 2022;8(2):35–40.
95. Evagora-Campbell M, Zahidie A, Buse K, Rabbani F, Hawkes S. Promoting labour migrant health equity through action on the structural determinants: A systematic review. Journal of migration and health. 2022;5:100082.
96. Evans N, Rohrer J, D’Alessandro M, Chhean E, Wilkniss S. Healthy People, Healthy States: Promising Practices to Address Health Disparities.
97. Fene F, Johri M, Michel ME, Reyes-Morales H, Pelcastre-Villafuerte BE. Multiple deprivations as drivers of suboptimal basic child vaccination in Latin America and the Caribbean: cross-sectional analysis of household survey data for 18,136 children across 211 regions in 15 countries. Int J Equity Health. 2025;24(1):1–17.
98. Fielding-Miller R, Dunkle KL, Hadley C, Cooper HL, Windle M. Agency as a mediator in the pathway from transactional sex to HIV among pregnant women in Swaziland: a multigroup path analysis. Journal of the International AIDS Society. 2017;20(1):21554.
99. Figueroa JF, Wadhera RK, Mehtsun WT, Riley K, Phelan J, Jha AK. Association of race, ethnicity, and community-level factors with COVID-19 cases and deaths across U.S. counties. Healthcare (Amsterdam, Netherlands). 2021;9(1):100495.
100. Flores G, Abbasi A, Korachais C, Lavado R. Unaffordability of COVID-19 tests: assessing age-related inequalities in 83 countries. Int J Equity Health. 2022;21(3):177.
101. Foreman KJ, Marquez N, Dolgert A, Fukutaki K, Fullman N, McGaughey M, et al. Forecasting life expectancy, years of life lost, and all-cause and cause-specific mortality for 250 causes of death: reference and alternative scenarios for 2016-40 for 195 countries and territories. Lancet (London, England). 2018;392(10159):2052–90.
102. Foryciarz A, Srivathsa N, Sedan O, Goldman Rosas L, Rose S. Causal modeling of chronic kidney disease in a participatory framework for informing the inclusion of social drivers in health algorithms. medRxiv : the preprint server for health sciences. 2025.
103. Frakt AB, Jha AK, Glied S. Pivoting from decomposing correlates to developing solutions: An evidence-based agenda to address drivers of health. Health Services Research. 2020;55 Suppl 2(Suppl 2):781–6.
104. Francis A, O'Sullivan KM, Patel P, Viecelli AK, Hedley JA, Swaminathan R, et al. Equity and diversity in the nephrology workforce in Australia and New Zealand. Internal medicine journal. 2022;52(11):1900–9.
105. Freitas Â, Rodrigues TC, Santana P. Assessing Urban Health Inequities through a Multidimensional and Participatory Framework: Evidence from the EURO-HEALTHY Project. Journal of urban health : bulletin of the New York Academy of Medicine. 2020;97(6):857–75.
106. Fried S, Harrison B, Starcevich K, Whitaker C, O'Konek T. Integrating interventions on maternal mortality and morbidity and HIV: a human rights-based framework and approach. Health and human rights. 2012;14(2):21–33.
107. Friedman J, Hansen H, Bluthenthal RN, Harawa N, Jordan A, Beletsky L. Growing racial/ethnic disparities in overdose mortality before and during the COVID-19 pandemic in California. Preventive Medicine. 2021;153:106845.
108. Friedman J, Saavedra-Avendaño B, Schiavon R, Alexander L, Sanhueza P, Rios-Polanco R, et al. Quantifying disparities in access to public-sector abortion based on legislative differences within the Mexico City Metropolitan Area. Contraception. 2019;99(3):160–4.
109. Friedman MR, Liu Q, Meanley S, Haberlen SA, Brown AL, Turan B, et al. Biopsychosocial Health Outcomes and Experienced Intersectional Stigma in a Mixed HIV Serostatus Longitudinal Cohort of Aging Sexual Minority Men, United States, 2008‒2019. American Journal of Public Health. 2022;112(S4):S452–S62.
110. Frisina Doetter L, Frisina PG, Preuß B. Pandemic Meets Endemic: The Role of Social Inequalities and Failing Public Health Policies as Drivers of Disparities in COVID-19 Mortality among White, Black, and Hispanic Communities in the United States of America. International Journal of Environmental Research and Public Health. 2022;19(22).
111. Fullman N, Correa GC, Ikilezi G, Phillips DE, Reynolds HW. Assessing Potential Exemplars in Reducing Zero-Dose Children: A Novel Approach for Identifying Positive Outliers in Decreasing National Levels and Geographic Inequalities in Unvaccinated Children. Vaccines. 2023;11(3):647.
112. Gautron JMC, Tu Thanh G, Barasa V, Voltolina G. Using intersectionality to study gender and antimicrobial resistance in low- and middle-income countries. Health policy and planning. 2023.
113. Ghosh AK, Geisler BP, Ibrahim S. Racial/ethnic and socioeconomic variations in hospital length of stay: A state-based analysis. Medicine. 2021;100(20):e25976.
114. Gibbs A, Jewkes R, Willan S, Washington L. Associations between poverty, mental health and substance use, gender power, and intimate partner violence amongst young (18-30) women and men in urban informal settlements in South Africa: A cross-sectional study and structural equation model. PloS one. 2018;13(10):e0204956.
115. Gibbs A, Willan S, Misselhorn A, Mangoma J. Combined structural interventions for gender equality and livelihood security: a critical review of the evidence from southern and eastern Africa and the implications for young people. Journal of the International AIDS Society. 2012;15 Suppl 1:1–10.
116. Gillman AS, Pérez-Stable EJ, Das R. Advancing Health Disparities Science Through Social Epigenomics Research. JAMA Network Open. 2024;7(7):e2428992–e.
117. Givens ML, Catlin BB, Johnson SP, Pollock EA, Faust VN, Inzeo PT, et al. What Do We Know About the Drivers of Health and Equity? A Narrative Review of Graphic Representations. Health Equity. 2020;4(1):446–62.
118. Gkiouleka, A., Wong, G., Sowden, S., Kuhn, I., Moseley, A., Manji, S., ... & Ford, J. (2024). Reducing health inequalities through general practice: a realist review and action framework. Health and Social Care Delivery Research, 12(7), 1-104.
119. Glazer KB, Howell EA. A way forward in the maternal mortality crisis: addressing maternal health disparities and mental health. Archives of Women's Mental Health. 2021;24(5):823–30.
120. Golembeski CA, R. Dong K, Irfan A. Carceral and Climate Crises and Health Inequities: A Call for Greater Transparency, Accountability, and Human Rights Protections. World Medical and Health Policy. 2021;13(1):69–96.
121. Gompers A, Lewis TT, Kramer MR. Structural racism and racial disparities in stroke mortality in the United States, 2021. Social Science & Medicine. 2025;366:N.PAG–N.PAG.
122. Gopal R. Social change communication: A panacea for HIV and AIDS?-an outlook of a program manager. East J Med. 2011;16(2):133–6.
123. Graboyes E, Cramer J, Balakrishnan K, Cognetti DM, López-Cevallos D, de Almeida JR, et al. COVID-19 pandemic and health care disparities in head and neck cancer: Scanning the horizon. Head & Neck. 2020;42(7):1555–9.
124. Gray BJ, Grey CNB, Hookway A, Homolova L, Davies AR. Differences in the impact of precarious employment on health across population subgroups: a scoping review. Perspectives in Public Health. 2021;141(1):37–49.
125. Green L, Ashton K, Bellis MA, Clemens T, Douglas M. ‘Health in All Policies’—A Key Driver for Health and Well-Being in a Post-COVID-19 Pandemic World. International Journal of Environmental Research and Public Health. 2021;18(18):9468.
126. Guilamo-Ramos V, Thimm-Kaiser M, Benzekri A, Chacón G, López OR, Scaccabarrozzi L, et al. The Invisible US Hispanic/Latino HIV Crisis: Addressing Gaps in the National Response. American journal of public health. 2020;110(1):27–31.
127. Halbert CH. Social Drivers of Cancer Risk and Outcomes among African American Men. Cancer Journal (United States). 2023;29(6):293–6.
128. Hall HI, Espinoza L, Benbow N, Hu YW, Urban Areas HIVSW. Epidemiology of HIV infection in large urban areas in the United States. PloS one. 2010;5(9):e12756.
129. Hamad R, Dore EC, Wright E, Steedle R, Cowan SK. Economic Policies to Address Poverty as a Fundamental Determinant of Health: Existing Evidence and Future Directions. Annual review of public health. 2025.
130. Hampton T. Societal drivers of antimicrobial resistance. ERS Monograph. 2023;2023(99):167–79.
131. Harper LJ, Kidambi P, Kirincich JM, Thornton JD, Khatri SB, Culver DA. Health Disparities: Interventions for Pulmonary Disease – A Narrative Review. CHEST. 2023;164(1):179–89.
132. Harrison EA, Ndulue U, Ojikutu BO. Restoring the Infant Mortality Rate as a Measure of Societal Health and Well-Being. New England Journal of Medicine. 2025;393(6):609–14.
133. Hartinger SM, Palmeiro-Silva YK, Llerena-Cayo C, Blanco-Villafuerte L, Escobar LE, Diaz A, et al. The 2023 Latin America report of the Lancet Countdown on health and climate change: the imperative for health-centred climate-resilient development. The Lancet Regional Health - Americas. 2024;33:100746.
134. Hatzenbuehler ML, Phelan JC, Link BG. Stigma as a fundamental cause of population health inequalities. American journal of public health. 2013;103(5):813–21.
135. Hawkes S, Evagora-Campbell M, Zahidie A, Rabbani F, Buse K. Protecting and promoting the rights of the ‘reserve army of labour’: a policy analysis of structural determinants of migrant worker health in Pakistan and Qatar. Health policy and planning. 2023.
136. Henderson R, Furlano JA, Claringbold SS, Cornect-Benoit A, Ly A, Walker J, et al. Colonial drivers and cultural protectors of brain health among Indigenous peoples internationally. Frontiers in public health. 2024;12:1346753.
137. Hing AK, Chantarat T, Fashaw-Walters S, Hunt SL, Hardeman RR. Instruments for racial health equity: A scoping review of structural racism measurement, 2019-2021. Epidemiologic Reviews. 2024;46(1):mxae002.
138. Holden TM, Simon MA, Arnold DT, Halloway V, Gerardin J. Structural racism and COVID-19 response: higher risk of exposure drives disparate COVID-19 deaths among Black and Hispanic/Latinx residents of Illinois, USA. BMC Public Health. 2022;22(1):312.
139. Huicho L, Vidal-Cárdenas E, Akseer N, Brar S, Conway K, Islam M, et al. Drivers of stunting reduction in Peru: A country case study. American Journal of Clinical Nutrition. 2020;112:816S–29S.
140. Hurwitz M, Bonomo J, Spitz J, Sharma G. Intersectionality and Social Drivers of Health in Cardiovascular Care. Methodist DeBakey cardiovascular journal. 2024;20(5):98–110.
141. Ingraham NE, Purcell LN, Karam BS, Dudley RA, Usher MG, Warlick CA, et al. Racial and Ethnic Disparities in Hospital Admissions from COVID-19: Determining the Impact of Neighborhood Deprivation and Primary Language. Journal of general internal medicine. 2021;36(11):3462–70.
142. Irizar P, Pan D, Kapadia D, Bécares L, Sze S, Taylor H, et al. Ethnic inequalities in COVID-19 infection, hospitalisation, intensive care admission, and death: a global systematic review and meta-analysis of over 200 million study participants. EClinicalMedicine. 2023;57:101877.
143. Javed Z, Valero-Elizondo J, Cainzos-Achirica M, Sharma G, Mossialos E, Parekh T, et al. Race, Social Determinants of Health, and Risk of All-Cause and Cardiovascular Mortality in the United States. Journal of racial and ethnic health disparities. 2023.
144. Jean-Francois B, Bailey Lash T, Dagher RK, Green Parker MC, Han SB, Lewis Johnson T. The Potential for Health Information Technology Tools to Reduce Racial Disparities in Maternal Morbidity and Mortality. Journal of Women's Health. 2021;30(2):274–9.
145. Jenkinson JIR, Wigle J, Richard L, Tibebu T, Orkin AM, Thulien NS, et al. Structural violence as a driver of COVID-19 vaccine hesitancy and low vaccine uptake among people experiencing homelessness in Toronto, Canada: A qualitative study. Social science & medicine (1982). 2024;365:117588.
146. Jernigan VBB, Maudrie TL, Lopez SV. Stagnating Life Expectancy Among American Indians and Alaska Natives: Understanding the Drivers and Recommendations for Research, Policy, and Practice. Annual Review of Public Health. 2025;46(1):193–211.
147. Ji X, Sohn H, Sil S, Castellino SM. Moving Beyond Patient-Level Drivers of Racial/Ethnic Disparities in Childhood Cancer. Cancer epidemiology, biomarkers & prevention : a publication of the American Association for Cancer Research, cosponsored by the American Society of Preventive Oncology. 2022;31(6):1154–8.
148. Jin C, Chen J, Fang J, Hua K, Fu Z, Chen X, et al. Global Pattern and Disease Heterogeneity Drivers in Aging Populations. GeoHealth. 2025;9(6):e2025GH001335.
149. Johns NE, Cata-Preta BO, Kirkby K, Arroyave L, Bergen N, Danovaro-Holliday MC, et al. Inequalities in Immunization against Maternal and Neonatal Tetanus: A Cross-Sectional Analysis of Protection at Birth Coverage Using Household Health Survey Data from 76 Countries. Vaccines. 2023;11(4):752.
150. Johns NE, Kirkby K, Goodman TS, Heidari S, Munro J, Shendale S, et al. Subnational Gender Inequality and Childhood Immunization: An Ecological Analysis of the Subnational Gender Development Index and DTP Coverage Outcomes across 57 Countries. Vaccines. 2022;10(11):1951.
151. Jones A, Lacy-Nichols J, Baker P, Thow AM, Martin JE, Daube M, et al. Chapter 5 Disrupting the commercial determinants of health. Medical Journal of Australia. 2021;214(8):S27–S31.
152. Kassebaum NJ, Barber RM, Bhutta ZA, Dandona L, Gething PW, Hay SI, et al. Global, regional, and national levels of maternal mortality, 1990–2015: a systematic analysis for the Global Burden of Disease Study 2015. The Lancet. 2016;388(10053):1775–812.
153. Katz IT, Thomson DR, Ravishankar S, Otwombe K, Macarayan ER, Novak C, et al. Intersectional forces of urban inequality and the global HIV pandemic: a retrospective analysis. BMJ global health. 2025;10(4).
154. Kavanagh MM, Norato LF, Friedman EA, Armbrister AN. Planning for health equity in the Americas: an analysis of national health plans. Revista Panamericana de Salud Pública. 2021;45:e29.
155. Keener Mast D, Skelton-Wilson S, Chung C, Fahrenbruch M, Lee S. Implementation Drivers of COVID‐19 Prevention Strategies in K‐12 School Settings: A Qualitative Analysis. The Journal of school health. 2025;95(8):587–96.
156. Kegler MC, Hermstad A, Haardörfer R, Arriola KJ, Gauthreaux N, Tucker S, et al. Evaluation Design for The Two Georgias Initiative: Assessing Progress Toward Health Equity in the Rural South. Health Educ Behav. 2023;50(2):268–80.
157. Kepper MM, Fowler LA, Kusters IS, Davis JW, Baqer M, Sagui-Henson S, et al. Expanding a behavioral view on digital health access: Drivers and strategies to promote equity. Journal of medical Internet research. 2024;26:e51355.
158. Kerr J, Northington T, Sockdjou T, Maticka-Tyndale E. Perceived Neighborhood Quality and HIV-related Stigma among African Diasporic Youth; Results from the African, Caribbean, and Black Youth (ACBY) Study. Journal of health care for the poor and underserved. 2018;29(2):651–63.
159. Khatri RB, Assefa Y, Durham J. Multidomain and multilevel strategies to improve equity in maternal and newborn health services in Nepal: perspectives of health managers and policymakers. Int J Equity Health. 2023;22(1):105.
160. Khatri RB, Assefa Y. Drivers of the Australian Health System towards Health Care for All: A Scoping Review and Qualitative Synthesis. BioMed Research International. 2023:1–19.
161. Klugman M, Xue X, Hosgood HD. Race/ethnicity and lung cancer survival in the United States: a meta-analysis. Cancer causes & control : CCC. 2019;30(11):1231–41.
162. Kovach KA, Lutgen CB, Callen EF, Hester CM. Informing the American Academy of family Physician's Health Equity strategy - an environmental scan using the Delphi technique. Int J Equity Health. 2019;18(1):97.
163. Kruize H, Droomers M, Van Kamp I, Ruijsbroek A. What Causes Environmental Inequalities and Related Health Effects? An Analysis of Evolving Concepts. International Journal of Environmental Research and Public Health. 2014;11(6):5807–27.
164. Lacey Q. Impact of the Social Determinants of Health on Adult Trauma Outcomes. Crit Care Nurs Clin North Am. 2023;35(2):223–33.
165. Lai Y, Li Y, Liu X, Shi Y, Qu F, Zhang X, et al. The impact of social and commercial determinants on the unequal increase of oral disorder disease burdens across global, regional, and national contexts. BMC Oral Health. 2025;25(1):1–16.
166. Lambie-Mumford H, Kennedy K. Commercial determinants of health: A new framework for studying relationships between food corporations and food charities in the UK. Social Science & Medicine. 2025;366:N.PAG–N.PAG.
167. Lavell A, Chávez Eslava A, Barros Salas C, Miranda Sandoval D. Inequality and the social construction of urban disaster risk in multi-hazard contexts: the case of Lima, Peru and the COVID-19 pandemic. Environment and urbanization. 2023;35(1):131–55.
168. LeBrón AMW, Michelen M, Cantero PJ, Montiel GI, Ruiz L, Razaghi K, et al. Pandemic-Related Funding Termination and Structural Drivers of Inequities: A Case Study of a Promotores/as-Focused Community-Based Organization. American journal of public health. 2025:e1–e12.
169. Lee D, Kett PM, Mohammed SA, Frogner BK, Sabin J. Inequitable care delivery toward COVID-19 positive people of color and people with disabilities. PLOS global public health. 2023;3(4):e0001499.
170. Lee J, Schram A, Riley E, Harris P, Baum F, Fisher M, et al. Addressing Health Equity Through Action on the Social Determinants of Health: A Global Review of Policy Outcome Evaluation Methods. International Journal of Health Policy and Management. 2018;7(7):581–92.
171. Leigh JP, Grood Cd, Ahmed SB, Ulrich AC, Fiest KM, Straus SE, et al. Toward Gender Equity in Critical Care Medicine: A Qualitative Study of Perceived Drivers, Implications, and Strategies. Critical care medicine. 2019;47(4):e286–e91.
172. Lewington J, Geddes R, Gabagaya G. Female empowerment to improve sexual and reproductive health outcomes and prevent violence in adolescent girls and young women in Uganda: evidence reviews for policy. Afr Health Sci. 2022;22(4):413–27.
173. Lewis NA, Porticella N, Liu J, Taylor T, Michener J, Barry CL, et al. Beyond fear of backlash: Effects of messages about structural drivers of COVID-19 disparities among large samples of Asian, Black, Hispanic, and White Americans. Social science & medicine (1982). 2025;377:118096.
174. Little M, Hagar H, Zivot C, et al. Drivers and health implications of the dietary transition among Inuit in the Canadian Arctic: a scoping review. Public Health Nutrition. 2021;24(9):2650-2668. doi:10.1017/S1368980020002402
175. Lobelo F, Bienvenida A, Leung S, Mbanya A, Leslie E, Koplan K, et al. Clinical, behavioural and social factors associated with racial disparities in COVID-19 patients from an integrated healthcare system in Georgia: a retrospective cohort study. BMJ open. 2021;11(5):e044052.
176. Loehrer AP, Cevallos PC, Jim√©nez RT, Wong SL. Reporting on Race and Racial Disparities in Breast Cancer: The Neglect of Racism as a Driver of Inequitable Care. Annals of surgery. 2023;277(2):329–34.
177. Lopez K, Norris K, Hardy M, Valentino LA. Defining the Impact of Social Drivers on Health Outcomes for People with Inherited Bleeding Disorders. Journal of Clinical Medicine. 2022;11(15):4443.
178. MacCarthy S, Poteat T, Xia Z, Roque NL, Kim A, Baral S, et al. Current research gaps: A global systematic review of HIV and sexually transmissible infections among transgender populations. Sexual Health. 2017;14(5):456–68.
179. Mackenzie M, Baruffati D, Lindsay C, O'Donnell K, Ellis D, Simpson S, et al. Fundamental causation and candidacy: Harnessing explanatory frames to better understand how structural determinants of health inequalities shape disengagement from primary healthcare. Social Science & Medicine. 2025;374:N.PAG–N.PAG.
180. Mahimbo A, Kang M, Sestakova L, Smith M, Dawson A. Factors influencing refugees' willingness to accept COVID-19 vaccines in Greater Sydney: a qualitative study. Australian and New Zealand journal of public health. 2022.
181. Manandhar M, Hawkes S, Buse K, Nosrati E, Magar V. Gender, health and the 2030 agenda for sustainable development. Bulletin of the World Health Organization. 2018;96(9):644–53.
182. Marmot, M., Allen, J., Goldblatt, P., Herd, E., & Morrison, J. (2020). Build back fairer: the COVID-19 Marmot review. The pandemic, socioeconomic and health inequalities in England.
183. Martinez Leal I, Njoh J, Chen TA, Foreman-Hays F, Reed BC, Haley SA, et al. Exploring COVID-19 Vaccine Attitudes among Racially and Ethnically Minoritized Communities: Community Partners’ and Residents’ Perspectives. International Journal of Environmental Research and Public Health. 2023;20(4):3372.
184. Martinez-Cardoso A, Jang W, Baig AA. Moving Diabetes Upstream: the Social Determinants of Diabetes Management and Control Among Immigrants in the US. Current diabetes reports. 2020;20(10):48.
185. Martins SS, Sampson L, Cerdá M, Galea S. Worldwide Prevalence and Trends in Unintentional Drug Overdose: A Systematic Review of the Literature. American Journal of Public Health. 2015;105(11):e29–e49.
186. Masters NB, Zhou T, Meng L, Lu PJ, Kriss JL, Black C, et al. Geographic Heterogeneity in Behavioral and Social Drivers of COVID-19 Vaccination. American journal of preventive medicine. 2022;63(6):883–93.
187. Mathews L, Brewer LC. A Review of Disparities in Cardiac Rehabilitation EVIDENCE, DRIVERS, AND SOLUTIONS. Journal of Cardiopulmonary Rehabilitation and Prevention. 2021;41(6):375–82.
188. Mayén A-L, de Mestral C, Zamora G, Paccaud F, Marques-Vidal P, Bovet P, et al. Interventions promoting healthy eating as a tool for reducing social inequalities in diet in low- and middle-income countries: a systematic review. Int J Equity Health. 2016;15(1):205.
189. McMaughan DJ, Oloruntoba O, Smith ML. Socioeconomic Status and Access to Healthcare: Interrelated Drivers for Healthy Aging. Frontiers in public health. 2020;8.
190. Menza TW, Zlot A, Gonzalez-Pena Y, Cicognani C, Pearson S, Li J, et al. Multilevel Drivers of Congenital Syphilis, Oregon, 2013 to 2021. Sexually Transmitted Diseases. 2025;52(1):1–8.
191. Mercado CI, Bullard KM, Bolduc MLF, Andrews CA, Freggens ZRF, Liggett G, et al. A Shift in Approach to Addressing Public Health Inequities and the Effect of Societal Structural and Systemic Drivers on Social Determinants of Health. Public Health Reports. 2024.
192. Mialon M. An overview of the commercial determinants of health. Globalization and health. 2020;16(1):74.
193. Mikhail JN, Nemeth LS, Mueller M, Pope C, NeSmith EG. The Social Determinants of Trauma: A Trauma Disparities Scoping Review and Framework. Journal of trauma nursing : the official journal of the Society of Trauma Nurses. 2018;25(5):266–81.
194. Morey BN, Penaia CS, Tulua A, Chang RC, Tanghal R, Tran JH, et al. Democratizing Native Hawaiian and Pacific Islander data: examining community accessibility of data for health and the social drivers of health. American Journal of Public Health. 2024;114(S1):S103–S11.
195. Mourali M, Novakowski D, Pogacar R, Brigden N. Public perception of accuracy-fairness trade-offs in algorithmic decisions in the United States. PLoS ONE. 2025;20(3 March).
196. Mujahid MS, Wall-Wieler E, Hailu EM, Berkowitz RL, Gao X, Morris CM, et al. Neighborhood disinvestment and severe maternal morbidity in the state of California. American journal of obstetrics & gynecology MFM. 2023;5(6):100916.
197. Mujica OJ, Zhang D, Hu Y, Espinosa IC, Araneda N, Dragomir A, et al. Inequalities in Violent Death across Income Levels among Young Males and Females in Countries of the Americas. International journal of environmental research and public health. 2023;20(7).
198. Mukumbang FC. Pervasive systemic drivers underpin COVID-19 vulnerabilities in migrants. Int J Equity Health. 2021;20(1):146.
199. Mustanski B, Birkett M, Kuhns LM, Latkin CA, Muth SQ. The Role of Geographic and Network Factors in Racial Disparities in HIV Among Young Men Who have Sex with Men: An Egocentric Network Study. AIDS and behavior. 2015;19(6):1037–47.
200. Mustanski B, Morgan E, D’Aquila R, Birkett M, Janulis P, Newcomb ME. INDIVIDUAL AND NETWORK FACTORS ASSOCIATED WITH RACIAL DISPARITIES IN HIV AMONG YOUNG MEN WHO HAVE SEX WITH MEN: RESULTS FROM THE RADAR COHORT STUDY. Journal of acquired immune deficiency syndromes (1999). 2019;80(1):24–30.
201. Nelson HO, Lange A, Shah MK, Kibet M. Structural inequities and provider burnout in maternal health in the United States. Social science & medicine (1982). 2025;372:118010.
202. Nielsen L, Marsland AL, Hamlat EJ, Epel ES. New Directions in Geroscience: Integrating Social and Behavioral Drivers of Biological Aging. Psychosomatic Medicine. 2024;86(5):360–5.
203. Noaeen M, Rostami A, Ghanem I, Saarela O, Keshavjee K, Brook J, et al. Mapping Neighborhood-Level Drivers of Type 2 Diabetes: A Predictive-Causal Approach for Precision Public Health. ResearchSquare. 2025.
204. Noel L, Cubbin C, Vohra-Gupta S. Engaging Community–Academic Partnerships: A Case Study of the Influence of Social Drivers of Health on Equitable Transitions of Cancer Care in the United States. Healthcare (2227-9032). 2024;12(2):264.
205. O'Campo P. Are we producing the right kind of actionable evidence for the social determinants of health? Journal of urban health : bulletin of the New York Academy of Medicine. 2012;89(6):881–93.
206. Odufalu F-D, Aboubakr A, Anyane-Yeboa A. Inflammatory bowel disease in underserved populations: lessons for practice. Current opinion in gastroenterology. 2022;38(4):321–7.
207. Ovbiagele B. The society for equity neuroscience (SEQUINS): Seeking to eliminate global brain health inequities and disparities through research. Equity neuroscience. 2025;1(1).
208. Pan CW, Dong XX, Lanca C, Wang Y, Saw SM, He X, et al. Global perspectives on myopia and pathologic myopia: From environmental drivers to precision medicine. Progress in Retinal and Eye Research. 2025;109:101415.
209. Pantoja-Ruiz C, Akinyemi R, Lucumi-Cuesta DI, Youkee D, Emmett E, Soley-Bori M, et al. Socioeconomic Status and Stroke: A Review of the Latest Evidence on Inequalities and Their Drivers. Stroke. 2025;56(3):794–805.
210. Pantoja-Ruiz C, Liu L, Lim E, Soley-Bori M, Kalansooriya W, Emmett E, et al. A Systematic Review of Causal Pathways of Socioeconomic Inequalities in Stroke. International journal of stroke : official journal of the International Stroke Society. 2025:17474930251399064.
211. Parra Moreno CF, Sánchez Cárcamo RA. Inseguridad ontológica y migraciones: análisis crítico del desarraigo. Investigación y Desarrollo. 2025;33(2):395–428.
212. Pedersen M, King AC. How Can Sport-Based Interventions Improve Health among Women and Girls? A Scoping Review. Int J Environ Res Public Health. 2023;20(6).
213. Peeters A, Blake MRC. Socioeconomic Inequalities in Diet Quality: from Identifying the Problem to Implementing Solutions. Curr Nutr Rep. 2016;5(3):150–9.
214. Petiwala A, Lanford D, Landers G, Minyard K. Community voice in cross-sector alignment: concepts and strategies from a scoping review of the health collaboration literature. BMC Public Health. 2021;21(1):712.
215. Philbin MM, Flake M, Hatzenbuehler ML, Hirsch JS. State-level immigration and immigrant-focused policies as drivers of Latino health disparities in the United States. Social Science and Medicine. 2018;199:29–38.
216. Pineros-Leano M, Pérez-Flores NJ, Damian K, Rodrigues K, Ortiz G, Simonovich SD. Mental health disparities in Latinx immigrant communities residing in the United States during COVID-19: Implications for policy and practice. Frontiers in public health. 2022;10:1000233.
217. Pinheiro FTdS, Tavares NBF, Silva JPX, Araújo AFd. Participação universitária em defesa do Sistema Único de Saúde. Saúde debate. 2020;44:504–15.
218. Pornpen SP. Exploration of the hidden financial hardship of chronic kidney disease under universal coverage in Thailand. 2023.
219. Powis M, Milley-Daigle C, Hack S, Alibhai S, Singh S, Krzyzanowska MK. Impact of the Early Phase of the COVID Pandemic on Cancer Treatment Delivery and the Quality of Cancer Care: A Scoping Review and Conceptual Model. International journal for quality in health care : journal of the International Society for Quality in Health Care. 2021.
220. Quinn KG, Walsh JL, DiFranceisco W, Edwards T, Takahashi L, Johnson A, et al. The Inherent Violence of Anti-Black Racism and its Effects on HIV Care for Black Sexually Minoritized Men. Journal of urban health : bulletin of the New York Academy of Medicine. 2024;101(1):23–30.
221. Ralston R, Godziewski C, Brooks E. Reconceptualising the commercial determinants of health: Bringing institutions in. BMJ Global Health. 2023;8(11):e013698.
222. Rashad H, Khadr Z. Measurement of health equity as a driver for impacting policies. Health promotion international. 2014;29(suppl 1):i68–i82.
223. Razdan S, Hedli LC, Sigurdson K, Profit J, Morton CH. Disparity drivers, potential solutions, and the role of a health equity dashboard in the neonatal intensive care unit: a qualitative study. Journal of Perinatology. 2024;44(5):659–64.
224. Reiner RC, Wiens KE, Deshpande A, Baumann MM, Lindstedt PA, Blacker BF, et al. Mapping geographical inequalities in childhood diarrhoeal morbidity and mortality in low-income and middle-income countries, 2000-17: Analysis for the Global Burden of Disease Study 2017. The Lancet. 2020;395(10239):1779–801.
225. Rüegg SR, McMahon BJ, Häsler B, Esposito R, Nielsen LR, Ifejika Speranza C, et al. A Blueprint to Evaluate One Health. Front Public Health. 2017;5:20.
226. Russell C, Schram A, Salmon L, Carrad A, Barbour L, Lacy-Nichols J, et al. Interdisciplinary insights on the future of food systems research: perspectives from the next generation of research leaders. Public health nutrition. 2022;25(11):3235–9.
227. Saigí - Rubió F, Torrent - Sellens J, Robles N, Pérez Palaci JE, Baena MI, Blyde J. Estudio sobre telemedicina internacional en América Latina: motivaciones, usos, resultados, estrategias y políticas. Monografía del BID. 2021(953):284–.
228. Salgado de Snyder VN, McDaniel M, Padilla AM, Parra-Medina D. Impact of COVID-19 on Latinos: a social determinants of health model and scoping review of the literature. Hispanic Journal of Behavioral Sciences. 2021;43(3):174–203.
229. Salvalaggio PR. Geographic disparities in transplantation. Curr Opin Organ Transplant. 2021;26(5):547–53.
230. Sanogo F, Jefferson M, Beard TA, Salhia B, Babatunde OA, Cho J, et al. Social and clinical drivers of stress responses in African American breast cancer survivors. Scientific Reports. 2024;14(1):19729.
231. Savage SR, Kraft SA, Tanner C, Houde M. Advancing action on the political determinants of health in the United States. Journal of public health policy. 2024;45(3):484–94.
232. Schaaf M, Boydell V, Topp SM, Iyer A, Sen G, Askew I. A summative content analysis of how programmes to improve the right to sexual and reproductive health address power. BMJ Global Health. 2022;7(4).
233. Schnake-Mahl AS, Jahn JL, Subramanian SV, Waters MC, Arcaya M. Gentrification, Neighborhood Change, and Population Health: a Systematic Review. Journal of Urban Health. 2020;97(1):1–25.
234. Schram A, Carrad A, Townsend B, Harris P, Baum F, Rychetnik L, et al. Doing (and undoing) privilege: evaluating how public policy drives health inequities. Critical Public Health. 2025;35(1):1–7.
235. Schultz S, McCracken J, Zorbas C, Yoong S, Peeters A, Backholer K. "Placing community in the driver's seat": Key strategies and critical enablers of an equitable place-based COVID-19 vaccination response. Health & place. 2024;91:103388.
236. Schulz AJ, Mehdipanah R, Chatters LM, Reyes AG, Neblett EW, Jr., Israel BA. Moving Health Education and Behavior Upstream: Lessons From COVID-19 for Addressing Structural Drivers of Health Inequities. Health Educ Behav. 2020;47(4):519–24.
237. Scott S, Nguyen PH, Neupane S, Pramanik P, Nanda P, Bhutta ZA, et al. Early marriage and early childbearing in South Asia: trends, inequalities, and drivers from 2005 to 2018. Ann N Y Acad Sci. 2021;1491(1):60–73.
238. Sen G, Reddy B, Iyer A. Beyond measurement: the drivers of disrespect and abuse in obstetric care. Reproductive Health Matters. 2018;26(53):6–18.
239. Serchen J, Doherty R, Atiq O, Hilden D. A Comprehensive Policy Framework to Understand and Address Disparities and Discrimination in Health and Health Care: A Policy Paper From the American College of Physicians. Annals of internal medicine. 2021;174(4):529–32.
240. Sharifi M, Sequist TD, Rifas-Shiman SL, Melly SJ, Duncan DT, Horan CM, et al. The role of neighborhood characteristics and the built environment in understanding racial/ethnic disparities in childhood obesity. Preventive medicine. 2016;91:103–9.
241. Shahidullah JD, Hostutler CA, Coker TR, Allmon Dixson A, Okoroji C, Mautone JA. Child health equity and primary care. American Psychologist. 2023;78:93–106.
242. Shearer JC, Nava O, Prosser W, Nawaz S, Mulongo S, Mambu T, et al. Uncovering the Drivers of Childhood Immunization Inequality with Caregivers, Community Members and Health System Stakeholders: Results from a Human-Centered Design Study in DRC, Mozambique and Nigeria. Vaccines. 2023;11(3):689.
243. Smitherman HC, Jr., Baker RS, Wilson MR. Socially Accountable Academic Health Centers: Pursuing a Quadripartite Mission. Academic medicine : journal of the Association of American Medical Colleges. 2019;94(2):176–81.
244. Socal MP, Amon JJ, Biehl J. Right-to-Medicines Litigation and Universal Health Coverage: Institutional Determinants of the Judicialization of Health in Brazil. Health and human rights. 2020;22(1):221–35.
245. Steinberg H, Trick W, Zawitz C, Weinstein RA, Won P, Aroutcheva A, et al. Incarceration is a key driver of racial disparities in community-associated methicillin-resistant Staphylococcus aureus infections in Cook County, IL. Annals of Epidemiology. 2025;109:96–102.
246. Swinburn BA, Sacks G, Hall KD, McPherson K, Finegood DT, Moodie ML, et al. The global obesity pandemic: shaped by global drivers and local environments. Lancet (London, England). 2011;378(9793):804–14.
247. Taggart T, Ritchwood TD, Nyhan K, Ransome Y. Messaging matters: achieving equity in the HIV response through public health communication. The Lancet HIV. 2021;8(6):e376–e86.
248. Taira BR. Optimizing Our Clinical Practice for Health Equity: Recognizing the Social Drivers of Health That Affect Physician Behavior. Annals of Emergency Medicine. 2019;74(5):S78–S81.
249. Thornton RLJ, Yang TJ. Addressing population health inequities: investing in the social determinants of health for children and families to advance child health equity. Current Opinion in Pediatrics. 2023;35(1):8–13.
250. Thorpe RJ, Bruce MA, Wilder T, Jones HP, Thomas Tobin C, Norris KC. Health Disparities at the Intersection of Racism, Social Determinants of Health, and Downstream Biological Pathways. International Journal of Environmental Research and Public Health. 2025;22(5):703.
251. Tong MW, Ziegeler K, Kreutzinger V, Majumdar S. Explainable AI reveals tissue pathology and psychosocial drivers of opioid prescription for non-specific chronic low back pain. Scientific reports. 2025;15(1):30690.
252. Towe VL, May LW, Huang W, Martin LT, Carman K, Miller CE, et al. Drivers of differential views of health equity in the U.S.: is the U.S. ready to make progress? Results from the 2018 National Survey of Health Attitudes. BMC Public Health. 2021;21(1):175.
253. Toyobo O, Guglielminotti J, Andreae DA, Andreae MH. Health equity research in obstetric anesthesia. Curr Opin Anaesthesiol. 2022;35(3):306–16.
254. Trani JF, Singh RK, Walker AIB, Bekena S, Zhu Y, Williams JP, et al. Structural and social determinants of dementia risk among adults racialized as Black: Results from a community-based system dynamics approach. Alzheimer's and Dementia. 2025;21(7):e70494.
255. Trauer JM, Dodd PJ, Gomes MGM, Gomez GB, Houben RMGJ, McBryde ES, et al. The Importance of Heterogeneity to the Epidemiology of Tuberculosis. Clinical infectious diseases : an official publication of the Infectious Diseases Society of America. 2019;69(1):159–66.
256. Trivedi PS, Guerra B, Kumar V, Akinwande G, West D, Abi-Jaoudeh N, et al. Healthcare Disparities in Interventional Radiology. J Vasc Interv Radiol. 2022;33(12):1459–67.e1.
257. Turan B, Hatcher AM, Weiser SD, Johnson MO, Rice WS, Turan JM. Framing Mechanisms Linking HIV-Related Stigma, Adherence to Treatment, and Health Outcomes. American journal of public health (1971). 2017;107(6):863–9.
258. Tziraki-Segal C, De Luca V, Santana S, Romano R, Tramontano G, Scattola P, et al. Creating a Culture of Health in Planning and Implementing Innovative Strategies Addressing Non-communicable Chronic Diseases. Frontiers in sociology. 2019;4:9.
259. Uzzi M, Ricard JR, Belton I, Linton S, Marineau L, Johnson RM, et al. Fatal Opioid Overdoses by Historical and Contemporary Neighborhood-Level Structural Racism. JAMA health forum. 2025;6(11):e253986.
260. Valier MR, Elam-Evans LD, Mu Y, Santibanez TA, Yankey D, Zhou T, et al. Racial and Ethnic Differences in COVID-19 Vaccination Coverage Among Children and Adolescents Aged 5-17 Years and Parental Intent to Vaccinate Their Children - National Immunization Survey-Child COVID Module, United States, December 2020-September 2022. MMWR Morbidity and mortality weekly report. 2023;72(1):1–8.
261. Van Doren VE, Spencer ML, Resnick D, Agrawal SS, Garcia MLW, Desai K, et al. Health Justice Standards in Graduate Medical Education: Moving from Performative to Concrete Change. Journal of General Internal Medicine. 2023;38(7):1705–8.
262. Vance AJ, Bell T. Becoming an Antiracist Neonatal Community. Adv Neonatal Care. 2021;21(1):9–15.
263. Vaughan AS, Quick H, Schieb L, Kramer MR, Taylor HA, Casper M. Changing rate orders of race-gender heart disease death rates: An exploration of county-level race-gender disparities. SSM Popul Health. 2019;7:100334.
264. Villalonga-Olives E, Kawachi I, Hernández-Aguado I. Social Capital during the First Wave of the COVID-19 Outbreak: The Case of the Island of Menorca. International journal of environmental research and public health. 2021;18(23).
265. Wainstein M, Marioli PA, Brown M, Abdul Hafidz MI, Neyra JA, Cervantes L. The Interchangeable Nature of Our Destinies: Health Inequities Born From Social and Structural Determinants of Health and the Advocacy Journey to Overcome Them. Advances in Kidney Disease and Health. 2025;32(3):301–8.
266. Wamoyi J, Mshana G, Mongi A, Neke N, Kapiga S, Changalucha J. A review of interventions addressing structural drivers of adolescents' sexual and reproductive health vulnerability in sub-Saharan Africa: implications for sexual health programming. Reproductive health. 2014;11(1):88.
267. Wang Y, Ghislandi S, Torbica A. Investigating the geographic disparity in quality of care: the case of hospital readmission after acute myocardial infarction in Italy. Eur J Health Econ. 2020;21(8):1149–68.
268. Ward J, Friche AAdL, Caiaffa WT, Proietti FA, Xavier CC, Roux AVD. Association of socioeconomic factors with body mass index, obesity, physical activity, and dietary factors in Belo Horizonte, Minas Gerais State, Brazil: <i>The BH Health Study</i>. Cadernos de Saúde Pública. 2015;31:182–94.
269. Watkinson RE, Sutton M, Turner AJ. Ethnic inequalities in health-related quality of life among older adults in England: secondary analysis of a national cross-sectional survey. The Lancet Public health. 2021;6(3):e145–e54.
270. Wesson DE, Mathur V, Tangri N, Hamlett S, Bushinsky DA, Boulware LE. Primary Medical Care Integrated with Healthy Eating and Healthy Moving is Essential to Reduce Chronic Kidney Disease Progression. The American journal of medicine. 2022;135(9):1051–8.
271. Whiteman A, Delmelle E, Rapp T, Chen S, Chen G, Dulin M. A Novel Sampling Method to Measure Socioeconomic Drivers of Aedes Albopictus Distribution in Mecklenburg County, North Carolina. Int J Environ Res Public Health. 2018;15(10).
272. Williams C, Vermund SH. Syndemic Framework Evaluation of Severe COVID-19 Outcomes in the United States: Factors Associated With Race and Ethnicity. Frontiers in Public Health. 2021;9:720264.
273. Wilpers AB, Lorch SA. Drivers of care and outcomes for people facing fetal conditions in the United States: a conceptual framework. Journal of Perinatology. 2025;45(9):1311–7.
274. Woodward EN, Singh RS, Ndebele-Ngwenya P, Melgar Castillo A, Dickson KS, Kirchner JE. A more practical guide to incorporating health equity domains in implementation determinant frameworks. Implement Sci Commun. 2021;2(1):61.
275. World Health Organization -PAHO 2019. Commission of the Pan American Health Organization on Equity and Health Inequalities in the Americas. Just Societies: Health Equity and Dignified Lives. Report of the Commission of the Pan American Health Organization on Equity and Health Inequalities in the Americas. Washington, D.C.: PAHO; 2019.
276. World Health Organization- EMRO. Build back fairer: achieving health equity in the Eastern Mediterranean Region: report of the commission on social determinants of health in the Eastern Mediterranean Region: executive summary: World Health Organization. Regional Office for the Eastern Mediterranean; 2022.
277. World Health Organization. Regional Office for E. Driving forward health equity: the role of accountability, policy coherence, social participation and empowerment: World Health Organization. Regional Office for Europe; 2019. 10 p.
278. Xia Y, Ma H, Moloney G, Velásquez García HA, Sirski M, Janjua NZ, et al. Geographic concentration of SARS-CoV-2 cases by social determinants of health in metropolitan areas in Canada: a cross-sectional study. CMAJ : Canadian Medical Association journal = journal de l'Association medicale canadienne. 2022;194(6):E195–E204.
279. Yoshihama M. Visualizing drivers of gender health disparities: Ongoing participatory action research following the 2011 disaster in Japan. Social Science and Medicine. 2021;283.
280. Young J. Critical Pedagogical Approaches to Structural Drivers of Health. AMA Journal of Ethics. 2025;27(9):E700–E7.
281. Young MF, Nguyen P, Tran LM, Avula R, Menon P. A Double Edged Sword? Improvements in Economic Conditions over a Decade in India Led to Declines in Undernutrition as Well as Increases in Overweight among Adolescents and Women. The Journal of Nutrition. 2020;150(2):364–72.
282. Yu XQ, Yap ML, Cheng ES, Ngo PJ, Vaneckova P, Karikios D, et al. Evaluating Prognostic Factors for Sex Differences in Lung Cancer Survival: Findings From a Large Australian Cohort. Journal of Thoracic Oncology. 2022;17(5):688–99.
283. Zere E, Oluwole D, Kirigia JM, Mwikisa CN, Mbeeli T. Inequities in skilled attendance at birth in Namibia: a decomposition analysis. BMC pregnancy and childbirth. 2011;11:34.
284. Zheng J, Tisdale RL, Heidenreich PA, Sandhu AT. Disparities in Hospital Length of Stay Across Race and Ethnicity Among Patients With Heart Failure. Circulation: Heart Failure. 2022;15(11):E009362.
285. Zirinsky E, Paintsil E, Oliveira CR. The clinical epidemiology of coronavirus disease 2019 in children and adolescents mirrors the widening gap in healthcare disparities. Current opinion in pediatrics. 2021;33(3):281–5.
286. Zulu JM, Crankshaw TL, Ouedraogo R, Juma K, Aantjes CJ. "The ones at the bottom of the food chain": structural drivers of unintended pregnancy and unsafe abortion amongst adolescent girls in Zambia. Arch Public Health. 2024;82(1):137.
