# Supplementary material for: Drivers of health equity in the Americas: a scoping review
Source: Int J Equity Health. 2026 Sep 16;25:255. doi: 10.1186/s12939-026-03031-6 (PMC13695892; doi:10.1186/s12939-026-03031-6)
Supplement: Supplementary file 3 [file 12939_2026_3031_MOESM4_ESM.docx]

**Drivers of Health Equity in the Americas**

**Supplementary File 4: Exhaustive List of Verbatim Definitions**

Note: The following table contains the complete list of documents identified in this review that provided an explicit definition integrating "drivers," "health," and "equity/inequity." (n=16).

| Author | Location | Topic / Domain | Verbatim Definition / Conceptual Approach |
| --- | --- | --- | --- |
| Auerbach (2011) | Global | Specific (HIV) | Key drivers of vulnerability **(Social/Structural).**:….interventions focused on individuals to a comprehensive strategy in which social/structural approaches are core elements. Such approaches aim to modify social conditions by addressing **key drivers of HIV vulnerability** that affect the ability of individuals to protect themselves and others from HIV. |
| Badal (2023) | Global | Specific (Biographical) | Biographical drivers **(social + emotional).:**: "AI should... **consider biographical drivers of health**, …....(Biography is broadly a person’s social, structural, and environmental exposures and affective emotional states" |
| Baker (2025) | Global | Specific (Commercial) | **Commercial and political power drivers**: "... the **key driver of the global rise in ultra-processed foods** (UPFs) is the growing economic and political power of the UPF industry, and its restructuring of food systems for profitability above all else - especially by the business practices of its leading corporations - in an increasingly financially-driven, capitalist world economy." |
| De Ornelas (2025) | Américas | Specific (Maternal) | **Drivers of racial inequity in health**: "We evaluated whether recent state laws and regulations in California sought to directly intervene on healthcare-based drivers of racial inequities in maternal health as conceptualized by a CDC-convened expert workgroup." |
| Evans (2023) | Américas | General/Health Equity | **Drivers of differences in health outcomes**: "These drivers begin upstream with structural discrimination which results in differences in social determinants of health (SDOH), health-related social needs (HRSNs), access to care, and, finally, differential quality of care within the health care system". |
| Hurwitz (2024) | Américas | General / Social drivers of health | **Social Drivers of Health**: "social, cultural, environmental, economic, and governmental factors that drive health behaviors, risk factors, and outcomes" |
| Kruize (2014) | Global | General/Structural Drivers | **Structural drivers**: “Structural drivers as included in the CDSH framework comprise the social, economic, and political mechanisms that create socioeconomic stratification, including the labor market, the educational system, political institutions and other cultural and societal values, as well as policies that may redistribute welfare”. In some environmental justice studies structural drivers are recognized as well. However, they have a different meaning, since they mainly refer to the community level and largely overlook the role of structural mechanisms at the societal level. |
| López (2022) | Global | General / Social drivers of health | **Social Drivers of Health** include psychosocial, environmental, socioeconomic, cultural, and governmental factors that impact health behaviors and outcomes." |
| Mercado (2024) | Américas | General//Structural Drivers | "**Structural drivers** are the macro-level systems (law, policy, economy) that shape the distribution of SDOH." |
| Mialon (2020) | Global | Specific (Commercial) | **Global dr ivers of ill-health** (Market-driven economies).:"business, market and political practices that are harmful to health and used to sell these commodities and secure a favourable policy environment" |
| Nielsen (2024) | Américas | Specific (Aging)* | **Social and behavioral drivers of biological aging** (Social exposome):"While geroscience traditionally has focused on the molecular and cellular drivers of biological aging, more fundamental causes of aging may be found in the social exposome... [including] social and behavioral drivers of biological aging". |
| Savage (2024) | Américas | Specific (Political -Drivers-Determinants) | "...**political determinants** are root causes that **drive** health disparities or health equity." |
| Serchen (2021) | Américas | General / Social drivers of health | "**Social drivers of health**, such as education, environment, employment, housing, and access to healthful foods and safe places to exercise, all directly and indirectly contribute to a person's health status." |
| Towe (2020) | Américas | General//Shared Health Value | "....**Drivers of making health a shared value** include the value of health; the role that social determinants of health play in influencing health; and a shared sense of community to influence health include the value of health; the role that social determinants of health play in influencing health; and a shared sense of community to influence health. Note: the definition include equity. |
| WHO-EURO (2019) | Global | General/Health Equity | **Institutional drivers of health equity**: "The four key drivers of health equity are: accountability, policy coherence, social participation and, underlying them, empowerment". |
| WHO-PAHO (2019) | Américas | General/Structural Equity | **Structural drivers of health inequalities in the Americas**: "structural factors, such as climate change, environmental threats, and one's relationship with the land, as well as the continuing impact of colonialism and racism, are also slowing progress towards a dignified life... the Commission [...] analyze the impact of drivers influencing health. |

*Note: 7 are related to a specific topic or condition.
