# Supplementary material for: Drivers of health equity in the Americas: a scoping review
Source: Int J Equity Health. 2026 Sep 16;25:255. doi: 10.1186/s12939-026-03031-6 (PMC13695892; doi:10.1186/s12939-026-03031-6)
Supplement: Supplementary file 4 [file 12939_2026_3031_MOESM5_ESM.docx]

**Drivers of Health Equity in the Americas**

**Supplementary File 5: drivers, methodologies, research gaps, and FTMs**

Table S5.1.	Drivers Mentioned or Interpreted

Table S5.2.	Methodological Strategies to Identify Health Equity Drivers

Table S5.3.	Research Gaps (identified & interpreted)

Table S5.4.	Theoretical and Conceptual Frameworks or Models (FTM) identified in the Americas

**Table S5.1. Drivers Mentioned or Interpreted (n=135)**

| Study | Topic | Mentioned or Interpreted Driver* | Effect Direction |
| --- | --- | --- | --- |
| Adamkiewicz 2011 | Moving environmental justice indoors: structural influences on residential exposure patterns | Structural driver: Institutional racism in land use; Public housing policy; Lack of political capital; Mediate driver: Indoor air quality (mold, pests, lead); Housing ventilation; Neighborhood proximity to traffic | Bidirectional |
| Agarwal 2020 | Racial-ethnic inequity in glycemic control and technology use among young adults with Type 1 Diabetes | Structural driver: Structural racism (clinical bias in technology prescription); Neighborhood disadvantage; Mediate driver: Socioeconomic status; Insurance type; Technology use (CGM/Pump); Psychosocial stress | Bidirectional |
| Alkureishi 2021 | Digital divide as a driver of health and social inequities | Structural driver: Technology access (broadband, devices); Digital literacy; Government/Policy regulation of internet costs; Mediate driver: Health literacy; Intergenerational support; Patient portal usability | Bidirectional |
| Bailey 2022 | Health inequality in five Caribbean Basin countries | Structural driver: Education; Socioeconomic status; Country/Geographic location; Mediate driver: Age; Gender; Place of residence | Negative |
| Bailey 2023 | Social and Structural drivers of health associated with transition outcomes for youth with chronic conditions | Structural driver: Structural racism; Health insurance policies; Systemic discrimination; Mediate driver: Economic instability; Housing; Health literacy; Social support | Bidirectional |
| Balaji 2012 | Role flexing and the influence of community, religion, and family on young Black MSM in the US South | Structural driver: Institutionalized heterosexism; Structural racism; Rigid gender norms; Mediate driver: Role flexing; Religious/familial expectations; Psychosocial stress | Bidirectional |
| Barber 2016 | Neighborhood disadvantage and cumulative biological risk (CBR) in African Americans | Structural driver: Neighborhood disadvantage (area-level SES, education, and employment); Mediate driver: Physical and social neighborhood environment; Psychosocial stress; Health behaviors | Bidirectional |
| Barber 2018 | Residential segregation and cardio-metabolic risk in Brazil. | Structural driver: Economic residential segregation; Structural racism (manifested as peripheralization); Mediate driver: Lack of urban services (sanitation, etc.); Neighborhood socioeconomic status | Negative |
| Barnes 2022 | Knowledge gaps and drivers of racial disparities in Alzheimer's disease among African Americans | Structural driver: Structural racism (education quality, neighborhood effects); Lack of diversity in clinical/biological data; Mediate driver: Vascular health (hypertension, diabetes); Educational attainment; Cognitive reserve | Bidirectional |
| Bedingfield 2022 | Systems of support and drivers of TB-related disparity among foreign-born patients and families | Structural driver: Foreign-born status; TB-related stigma; Lack of access to government social programs; Mediate driver: Language barriers; Intra-family support; Psychosocial stress (fear/isolation) | Bidirectional |
| Bhavsar 2024 | Development of SEED, a data visualization platform to explore social, environmental, and equity drivers of health | Structural driver: Equity Drivers (structural racism, social vulnerability); Environmental Drivers (air quality, green space); Mediate driver: Social Drivers (food access, housing, transportation); Health System access | Bidirectional |
| Brown TH 2022 | Structural racism as a driver of racial inequities in COVID-19 mortality at the state level. | Structural driver: Structural Racism (State-level racial regimes); Political representation gap; Mediate driver: Economic stratification (Poverty & Unemployment gaps); Residential segregation; Educational gap | Negative |
| Caffe 2017 | Strategic approaches to accelerate progress on adolescent pregnancy in LAC | Structural driver: Gender inequality; Poverty; Lack of educational/economic opportunities; Restrictive legal frameworks; Mediate driver: Access to sexual/reproductive health services; Comprehensive sexuality education; Sexual violence | Bidirectional |
| Campbell 2021 | Contribution of individual, community, and health system factors on glycemic control (HbA1c) among inner-city African Americans | Structural driver: Community Factors (Crime exposure, Neighborhood resources); Health System Access (Travel time >60 min); Mediate driver: Medication adherence; Coping/Trauma; Provider communication | Bidirectional |
| Chalasani 2022 | Determinants, drivers, and pathways to ensure equal access to high-quality evidence-based medication | Structural driver: Social policy (housing, justice); Health policy (drug pricing, insurance coverage); Structural racismo; Mediate driver: Provider/implicit bias; Pharmacy access; Patient trust; Language barriers | Negative |
| Chandra 2016 | Drivers of "Health as a Shared Value," including mindset, expectations, sense of community, and civic engagement within the Culture of Health framework | Structural driver: Social cohesion; Collective mindset/expectations; Sense of community; Mediate driver: Civic engagement; Community sense, investments; Social-ecological environment | Positive |
| Coates 2020 | Vision for health equity in the Americas post-COVID, based on the PAHO Independent Commission report | Structural driver: Colonialism; Structural racism; Gender discrimination; Economic models; Mediate driver: Daily living conditions (work, housing); Health system access | Negative |
| Cole 2023 | Gentrification, impacts on health equity and strategies for mitigation | Structural driver: Gentrification; Urban neoliberalism; Real estate market policy; Structural racism; Mediate driver: Physical and cultural displacement; Housing insecurity; Loss of social networks; Environmental exclusion (Green gentrification) | Bidirectional |
| Correll-King 2025 | Structural and social drivers of firearm violence exposure among LGBTQ+ young adults of color in Detroit | Structural driver: Historical/Contemporary structural racism (Redlining); Cissexism; Heterosexism; Economic deprivation; Mediate driver: Absence of social safety; Threatened masculinity (perpetrator status); Survival sex work; Perceived risk management | Bidirectional |
| Credit 2020 | Neighborhood-level drivers of racial and ethnic disparities in COVID-19 testing and infection rates in Chicago and NYC | Structural driver: Residential segregation (neighborhood racial composition); Built environment (density/walkability); Socioeconomic status; Mediate driver: Occupational exposure; Multi-generational households; Social vulnerability; Testing accessibility | Bidirectional |
| Cros 2019 | Inequities in health service utilization and catastrophic health expenditures in post-earthquake Haiti. | Structural driver: Household wealth (socioeconomic status); Geographic location (urban/rural); National health policy (lack of universal coverage); Mediate driver: Out-of-pocket payments; Educational attainment; Perceived severity of illness | Negative |
| Dalsania 2020 | Racial disparities in COVID-19 mortality | Structural driver: Structural racism (county composition); Lack of health insurance; Lower socioeconomic status; Mediate driver: Severe housing problems (crowding); Low birthweight; Diabetes prevalence; Air pollution | Negative |
| Darling-Hammond 2025 | Racial centrality as a buffer against the effects of chronic stress on suicide ideation and attempts among Black Americans | Structural driver: Chronic stress (structural racism); Racial centrality (identity); Mediate driver: Suicide ideation; Suicide attempts; Psychological resilience | Bidirectional |
| De Ornelas 2025 | Legal and regulatory reforms in California (2019-2023) targeting healthcare-based drivers of racial inequities in maternal health | Structural driver: State legislation; Insurance policy (Medi-Cal expansion); Structural racism; Mediate driver: Implicit bias training; Standardization of care; Community-based care models (doulas); Data transparency | Positive |
| Derose 2020 | Food insecurity among women living with HIV in the Dominican Republic | Structural driver: HIV-related stigma; Gender inequality; Economic marginalization; Mediate driver: Food insecurity; Unemployment; Social support systems | Negative |
| Dharma 2025 | Systemic and interpersonal bias in violence risk assessments within acute psychiatric care settings | Structural driver: Structural racism; Mode of admission (police involvement); Gendered stereotypes; Institutional safety protocols; Mediate driver: Perceived vs. actual risk; Clinical diagnosis (e.g., psychosis); Socioeconomic status (homelessness) | Negative |
| DiPetrillo 2024 | Interventions addressing racism in the US and the integration of health equity principles (HEART framework) | Structural driver: Structural racism; Systemic policy; Institutional governance; Mediate driver: Interpersonal racism; Community engagement; Physical environment; Healthcare service delivery | Bidirectional |
| Ditah 2019 | Main drivers of early-age diagnosis of congestive heart failure in the USA | Structural driver: Poverty; Lack of health insurance; Structural racism; Mediate driver: Obesity; Undiagnosed/unmanaged hypertension | Negative |
| Dove-Medows 2025 | Racism in black women during prenatal care | Structural driver: Structural and interpersonal racism; Intersectionality (race/gender); Socioeconomic inequality; Mediate driver: Blame-related communication; Internalized blame; Patient-provider interaction; Health services engagement | Negative |
| Duchowny 2020 | Life course trauma and muscle weakness trajectories among older adults, stratified by gender and race/ethnicity | Structural driver: Structural racism; Gender; Life course "institutional patterns"; Mediate driver: Childhood trauma; Early adulthood trauma; Chronic inflammation (IL-6, CRP); HPA axis dysfunction | Negative |
| Dwyer-Lindgren 2017 | Geographic inequalities in life expectancy across U.S. counties | Structural driver: Socioeconomic factors (income, education); Geographic location; County-level demographics; Mediate driver: Behavioral and metabolic risk factors (smoking, obesity, diabetes); Healthcare access and quality | Negative |
| Egede 2022 | Cross-sector collaborations between healthcare systems and community partners to target health equity in diabetes care | Structural driver: Food and housing policy; Structural racism; Neighborhood environment; Mediate driver: Food insecurity; Intersectoral partnerships; Financial incentives; coaching | Bidirectional |
| English 2021 | Impact of state-level structural racism and anti-LGBTQ policies on the mental and behavioral health of Black and White sexual minority men | Structural driver: State-level structural racism (incarceration, economic gaps); Anti-LGBTQ legislation; Mediate driver: Minority stress; Psychosocial distress (depression/anxiety); Substance use; Social support | Negative |
| Estime 2022 | Health disparities in trauma care and interventions to improve equitable outcomes | Structural driver: Geography (trauma deserts); Structural racism; Health insurance policy; Economic status; Mediate driver: In-hospital clinical practice; Access to rehabilitation; Quality improvement frameworks; Social support | Bidirectional |
| Evans 2023 | State-level policy strategies and promising practices to address health disparities and social drivers of health | Structural driver: State governance; Legislative mandates; Data infrastructure; Economic policy (taxpayer stewardship); Mediate driver: Community health workers; Inter-agency coordination; Value-based payments; Culturally competent care | Positive |
| Fene 2025 | Multiple social deprivations as drivers of suboptimal basic child vaccination (in 15 LAC countries | Structural driver: Regional/geographic inequality; Maternal education; Gender-related barriers; Mediate driver: Multidimensional poverty; Lack of prenatal care; Household size; Urban/rural location | Negative |
| Figueroa 2020 | Community-level factors (housing, occupation, poverty) driving racial/ethnic disparities in COVID-19 cases and deaths across U.S. counties | Structural driver: Structural racism (as proxied by county composition); Residential crowding; Socioeconomic deprivation; Mediate driver: Essential work status; Poverty; Air pollution | Negative |
| Foryciarz 2025 | Participatory causal modeling to include social drivers in chronic kidney disease algorithms | Structural driver: Structural racism; Socioeconomic status (income/insurance); Healthcare system design; Mediate driver: Doctor-patient trust; Care coordination; Nutrition access; Psychosocial stress | Bidirectional |
| Frakt 2020 | Quantifying the relative contribution of social drivers to evaluating evidence-based solutions and interventions | Structural driver: Public policy; Economic and environmental systems; Healthcare delivery models; Mediate driver: Program evaluations; Resource allocation; Evidence-based social interventions | Positive |
| Friedman 2021 | Rapidly growing racial/ethnic disparities in overdose mortality in California before and during the COVID-19 pandemic | Structural driver: Structural racism; COVID-19 pandemic; Economic instability; Mediate driver: Fentanyl and methamphetamine prevalence; Barriers to addiction treatment; Psychosocial stress | Negative |
| Friedman J 2019 | Impact of legislative differences and travel time on access to legal abortion in the Mexico City Metropolitan Area | Structural driver: Legislative geography (legality); Socioeconomic status (SES); Mediate driver: Travel time; Education level; Local service availability | Negative |
| Ghosh 2021 | Impact of race/ethnicity and socioeconomic status on hospital length of stay (LOS) | Structural driver: Socioeconomic status (household income); Structural racismo; Mediate driver: Discharge destination; Social support; Insurance type; Implicit bias; Healthcare coordination | Negative |
| Gillman 2024 | Social epigenomics across the life course | Structural driver: Structural racism; Neighborhood SES; Economic deprivation; Maternal occupational exposure; Mediate driver: Childhood adversity; DNA methylation; Epigenetic aging (biological clock); Parental practices; Dietary quality | Bidirectional |
| Glazer 2021 | Role of maternal health disparities and mental health in the US maternal mortality crisis | Structural driver: Structural racism; Fragmented healthcare systems; Socioeconomic inequities; Mediate driver: Poor care coordination; Inadequate risk assessment; Mental health/substance use disorders; Self-harm (suicide/overdose) | Bidirectional |
| Golembeski 2021 | Convergence of carceral and climate crises as drivers of health inequities | Structural driver: Structural racism; Hyper incarceration; Anthropogenic climate change; Economic deprivation; Mediate driver: Medical vulnerability; Environmental exposure (heat, disasters); Infectious diseases; Displacement | Bidirectional |
| Gompers 2025 | County-level structural racism as a driver of Black-White disparities in stroke mortality | Structural driver: Structural racism index (income, housing, education, insurance, incarceration); Residential segregation; Mediate driver: Gender (intersectionality); Psychosocial stress; Economic deprivation; Healthcare access | Negative |
| Guilamo-Ramos 2020 | HIV crisis and increasing incidence among Hispanic/Latino populations in the US | Structural driver: Lack of focused federal investment; Health insurance disparities; Migration-related barriers; Mediate driver: HIV stigma; Culturally unadapted behavioral interventions; Community leadership gaps. | Negative |
| Halbert 2023 | Social drivers of cancer risk, in African American men | Structural driver: Structural racism; Residential segregation; Systemic discrimination; Mediate driver: Psychosocial stress; Biological stress response (allostatic load); Social support/loneliness; Healthcare access | Negative |
| Hamad 2025 | Economic policies as fundamental drivers to address poverty and improve population health | Structural driver: Poverty; Income inequality; Economic policies (EITC, minimum wage, universal basic income); Mediate driver: Financial security; Stress reduction; Nutritional access; Healthcare utilization | Bidirectional |
| Harper 2023 | Interventions addressing racial and ethnic disparities in pulmonary diseases | Structural driver: Structural racism; Residential segregation; Environmental policy; Economic inequality; Mediate driver: Housing quality; Access to specialists; Health literacy | Bidirectional |
| Harrison 2025 | Reframing infant mortality rate (IMR) from an individual clinical outcome to a structural measure of societal health | Structural driver: Structural racism; Social protection policies; Housing inequity; Economic stability; Mediate driver: Prenatal care access; Maternal stress; Nutrition; Community support systems | Bidirectional |
| Hartinger 2024 | Impact of climate change on health equity and resilience in Latin America | Structural driver: Climate change; Environmental degradation; Unplanned urbanization; Economic instability; Mediate driver: Food insecurity; Air pollution (PM2.5); Health literacy; Infectious disease transmission (vectors) | Bidirectional |
| Hatzenbuehler 2013 | Stigma as a fundamental cause of population health inequalities | Structural driver: Structural stigma (institutional policies/norms); Social and economic resource depletion; Mediate driver: Psychosocial stress; Coping behaviors; Social relationship disruption | Bidirectional |
| Hing 2024 | Instruments to measure structural racism as a fundamental driver of health | Structural driver: Structural racism (Fundamental); Residential segregation; Economic inequality; Political governance; Mediate driver: Incarceration rates; Voting access; Housing quality; Healthcare institutional bias | Negative |
| Holden 2022 | Structural racism and COVID-19 deaths driven by unequal exposure in Illinois, USA | Structural driver: Structural racism; Residential segregation; Occupational mobility policies; Mediate driver: Higher infection risk (exposure); Access to diagnostic testing; Targeted public health programs | Bidirectional |
| Huicho 2020 | Key enablers for the reduction of child stunting in Peru | Structural driver: High-level political commitment; Results-based budgeting; Economic growth; Poverty reduction programs; Mediate driver: Immunization coverage; Water and sanitation; Maternal education; Conditional cash transfers | Bidirectional |
| Hurwitz 2024 | Intersectionality and social drivers of health (SDOH) in cardiovascular care | Structural driver: Structural racism; Socioeconomic status; Environmental factors; Discrimination; Mediate driver: Psychosocial stress; Health behaviors; Healthcare access; Intersectionality | Bidirectional |
| Ingraham 2021 | Impact of neighborhood deprivation and primary language on COVID-19 hospitalizations | Structural driver: Neighborhood deprivation (ADI); Structural racism; Mediate driver: Primary language (non-English); Comorbidities; Essential worker status | Negative |
| Javed 2023 | Effects of race and cumulative SDoH burden on all-cause and CVD mortality | Structural driver: Cumulative social disadvantage (SDoH Quintiles); Racial inequity in SDoH distribution; Mediate driver: SDoH burden (mediating 40-60% of racial disparities); Cardiovascular risk factors | Negative |
| Jean-Francois 2020 | Health IT tools as levers to reduce racial disparities in maternal morbidity and mortality | Structural driver: Geographic and socioeconomic disparities; Structural racism; Implicit bias in clinical care; Mediate driver: Adherence to clinical guidelines; Telemedicine access; Integration of SDoH data in EHR; Coordinated care | Bidirectional |
| Jenkinson 2024 | Structural violence as a driver of COVID-19 vaccine hesitancy among homeless people in Toronto | Structural driver: Structural violence (homelessness); Economic/Social deprivation; Policy-driven "placelessness"; Mediate driver: Enacted stigma; Healthcare service disruption; Lack of institutional trust; Personal agency/bodily autonomy | Bidirectional |
| Jernigan 2025 | Drivers of stagnating life expectancy among American Indians and Alaska Natives | Structural driver: Colonialism; Systematic racism; Land dispossession; Federal termination policies; Mediate driver: Loss of food sovereignty; Chronic underfunding of health services (IHS); Intergenerational trauma | Negative |
| Ji Xu 2022 | Multi-level drivers of racial/ethnic disparities in childhood cancer survival | Structural driver: Structural racism; Public insurance policies (ACA); Geographic deprivation; Mediate driver: Family social circumstances; Language barriers; Healthcare system mistrust; Clinical trial inclusivity | Bidirectional |
| Kavanagh 2021 | Analysis of 32 national health plans in the Americas regarding health equity | Structural driver: National health governance; Political commitment to equity; Marginalized group identification; Mediate driver: Participatory processes; Time-bound targets; Physical accessibility; Affordable access to medicines | Bidirectional |
| Keener Mast 2025 | Qualitative analysis of implementation drivers for COVID-19 prevention in K-12 schools | Structural driver: Political climate; Physical and financial resources; Federal/State guidance; Mediate driver: Staffing capacity; State agency support; Communication strategies; Student socio-economic needs | Bidirectional |
| Kegler 2022 | Comprehensive evaluation of rural coalitions addressing health equity in Georgia, USA | Structural driver: Racial and environmental justice; Affordable housing; Rural poverty; Mediate driver: Leadership capacity; Community readiness; Access to care; Public official engagement | Bidirectional |
| Kepper 2024 | Multilevel drivers of digital health inequities and behavioral strategies for equity | Structural driver: Digital redlining; Broadband infrastructure; Funding/Policy structures (reimbursement); Mediate driver: Digital health literacy; User-centered design; Data privacy/Trust; Clinical workflow integration | Bidirectional |
| Kerr 2018 | Intersection of neighborhood quality and HIV-related stigma among ACB youth. | Structural driver: Neighborhood social disorder; Religious/Cultural identity; Socio-environmental disadvantage; Mediate driver: HIV knowledge; Gender (masculinity); Experiences of discrimination | Bidirectional |
| Klugman 2019 | Lung cancer survival | Structural driver: Hispanic/Asian ethnicity (paradoxical protective effect); Marital status (social support); Mediate driver: Smoking status; Treatment receipt (Surgery/Radiation/Chemo); Comorbidities | Bidirectional |
| Kovach 2019 | Organizational strategies and priorities for advancing health equity in primary care | Structural driver: Institutional leadership; Health policy advocacy; Strategic planning for equity; Mediate driver: Workforce diversity; Research on SDOH; Clinical integration of social data | Bidirectional |
| Lacey 2023 | Impact of SDOH on adult trauma outcomes | Structural Drivers: Residential segregation ("trauma deserts"), unequal geographic distribution of Level I/II trauma centers, and health insurance policies (public vs. private coverage); Mediate Drivers: Emergency logistics (pre-hospital response times), clinical implicit bias in acute care, and restricted access to high-quality post-acute rehabilitation. | Negative |
| Lavell 2023 | Social construction of disaster risk and COVID-19 in Lima, Peru | Structural driver: Informal/Unequal urbanization; High labor informality; Historical lack of urban planning; Mediate driver: Institutional fragmentation; Lack of water and sanitation infrastructure; Household overcrowding | Negative |
| LeBrón 2025 | Post-pandemic "benefits cliff" and the role of community-based organizations | Structural driver: Termination of emergency funding; Structural exclusion of immigrants from safety nets; Mediate driver: Promotores/as models; Economic instability; Community-based infrastructure | Bidirectional |
| Lee 2023 | Inequitable care delivery for people of color and people with disabilities during COVID-19 | Structural driver: Structural racism; Structural ableism; Crisis standards of care/Rationing policies; Mediate driver: Implicit bias; Communication/Language barriers; Medical mistrust | Negative |
| Lewis 2025 | Effects of structural messaging on COVID-19 disparity perceptions and advocacy | Structural driver: Structural racism (segregation); Essential work conditions; Policy-driven social fragmentation; Mediate driver: Causal attributions; Government accountability; Collective action intentions; Message framing | Bidirectional |
| Little 2020 | Drivers of dietary transition and health implications among Canadian Inuit | Structural driver: Climate change; Colonial history/Settlement policies; Globalized food markets; Mediate driver: Cost of market food; Loss of traditional hunting skills; Time constraints (wage labor) | Negative |
| Lobelo 2021 | Clinical, behavioral, and social factors in COVID-19 racial disparities | Structural driver: Neighborhood deprivation; Residential segregation; Structural racism (employment); Mediate driver: Comorbidities (Hypertension/Diabetes); Housing density; Social environment | Negative |
| Loehrer AP 2023 | Racism as a neglected driver in breast cancer surgical | Structural driver: Structural racism (unnamed in most literature); Research reporting guidelines; Institutional power in academia; Mediate driver: Surgical care quality; Definition/contextualization of race; Socioeconomic mediators | Negative |
| Martínez Leal 2023 | Perspectives on COVID-19 vaccine attitudes among minoritized communities | Structural driver: Historical medical racism; Structural access barriers; Institutional distrust; Mediate driver: Misinformation; Cultural/Linguistic relevance; Community/Religious leadership | Bidirectional |
| Martínez-Cardoso 2020 | Social determinants of diabetes management and control among US immigrants | Structural driver: Immigration policies; Structural racism/xenophobia; Mediate driver: Material precarity (housing/food); Uninsurance; Linguistic barriers | Negative |
| Masters 2022 | Geographic heterogeneity in behavioral and social drivers of COVID-19 vaccination | Structural driver: Geographic region; Public health infrastructure/policy; Mediate driver: Vaccine confidence; Social norms; Access barriers | Bidirectional |
| Mathews 2021 | Evidence, drivers, and solutions for disparities in Cardiac Rehabilitation | Structural driver: Geographic distribution of centers; Insurance/Reimbursement policies; Mediate driver: Clinician referral bias; Transportation/Logistics; Social support | Bidirectional |
| Menza 2025 | Multi-level drivers of congenital syphilis (individual and county-level) | Structural driver: County-level unemployment; Childhood poverty; Violent crime rates; Mediate driver: Injection drug use; History of incarceration; Late/No prenatal care | Negative |
| Mercado 2024 | A shift in approach: The Societal Structural and Systemic Drivers (SSD) model | Structural driver: Societal, structural, and systemic drivers (10 dimensions including structural racism and economic systems); Mediate driver: Social Determinants of Health (SDOH); Intersectionality; Policy implementation | Bidirectional |
| Mikhail 2018 | Socioecological framework of the social determinants of trauma disparities. | Structural driver: Institutional power; Social context/Place; Structural segregation; Mediate driver: Discrimination experiences; Hospital/Workforce factors; Patient comorbidities | Bidirectional |
| Morey 2024 | Democratizing data for Native Hawaiians and Pacific Islanders communities to achieve health and social equity | Structural driver: Federal data governance; Structural racism (statistical invisibility); Mediate driver: Data accessibility (salience/cost); Technical capacity; Community trust | Bidirectional |
| Mourali 2025 | Public perception of accuracy-fairness trade-offs in algorithmic decision-making | Structural driver: Algorithmic systems implementation; Systemic algorithmic bias; Mediate driver: Accuracy-fairness trade-off; Public perception of justice; Resource allocation models | Bidirectional |
| Mujahid 2023 | Neighborhood deprivation and severe maternal morbidity | Structural driver: Structural disinvestment; Structural racism; Residential segregation; Mediate driver: Neighborhood Deprivation Index (NDI); Healthcare access; Environmental stressors | Negative |
| Mujica 2023 | Income-related inequalities in violent deaths among youth in the Americas | Structural driver: Income inequality; National wealth (GDP per capita); Mediate driver: Relative socioeconomic disadvantage; Gender; Access to prevention services. | Negative |
| Mustanski 2015 | Geographic and network drivers of HIV disparities among YMSM. | Structural driver: Residential segregation; Network racial homophily; Mediate driver: Neighborhood HIV prevalence; Network mixing patterns; Social infrastructure | Negative |
| Mustanski 2019 | Individual, network, and social drivers of HIV disparities among YMSM | Structural driver: Residential segregation; Network homophily; High community viral load; Mediate driver: Network density/transitivity; Housing instability; Neighborhood safety | Negative |
| Nelson 2025 | Structural inequities and provider burnout in US maternal health | Structural driver: Marginalization of maternal health as a field; Healthcare system shortages; Structural racism; Mediate driver: Triple marginalization; Interprofessional hierarchies; Provider identity (race/ethnicity); Resource constraints | Negative |
| Nielsen 2024 | Integrating social and behavioral drivers into biological aging (Geroscience) | Structural driver: Social exposome; Socioeconomic environment; Physical exposures; Mediate driver: Psychosocial stress; Epigenetic changes; Multisystem biological functioning | Bidirectional |
| Noaeen 2025 | Neighborhood-level drivers of Type 2 Diabetes | Structural driver: Food environment; Built environment (Walkability); Material deprivation; Mediate driver: Lifestyle behaviors; Healthcare access | Negative |
| Noel 2024 | Influence of social drivers on equitable transitions of cancer care | Structural driver: Geographic distribution of care; Structural racism; Economic inequity; Mediate driver: SDOH in care transitions; Community-academic partnerships; Patient navigation | Bidirectional |
| Odufalu 2022 | Healthcare disparities and equity in Inflammatory Bowel Disease | Structural driver: Structural racism; Historical injustice; Neighborhood deprivation; Mediate driver: Access to biologics/specialty care; Implicit bias; Social determinants of health (SDOH) | Bidirectional |
| Parra Moreno 2025 | Ontological insecurity and structural inequality as drivers of migration | Structural driver: Structural inequality (Gini Index); Income concentration (Top 10%); Wealth distribution; Mediate driver: Ontological insecurity; Relative deprivation; Social conflict (incarceration) | Negative |
| Petiwala 2021 | Sector collaboration | Structural: Institutional power sharing; Mediate: Community voice | Bidirectional |
| Philbin 2018 | State-level immigration policies as drivers of health disparities | Structural: State-level policy climate; Mediate: Access to health/education/transportation; Psychosocial stress (fear); Economic stability | Bidirectional |
| Pinhero de Souza 2020 | Social participation | Structural: Institutional social control; Mediate: Academic engagement | Bidirectional |
| Quinn 2024 | Anti-Black racism, mental health, and HIV care outcomes | Structural driver: Structural racism; Systemic healthcare exclusión; Mediate driver: Racial discrimination; Depressive symptoms; LGBTQ community support | Bidirectional |
| Razdan 2024 | Exploration of NICU disparities and the role of equity dashboard | Structural driver: Institutional care barriers; Hospital/Public policy, Mediate driver: Parental engagement (SDoH-linked); Interpersonal bias; Racial/Cultural concordance. | Negative |
| Saigí 2021 | Telemedicine in LAC | Structural driver: Public policy/Legal frameworks; Technological infrastructure; Digital divide; Mediate driver: Institutional management; Professional digital literacy; Productivity/Quality goals | Bidirectional |
| Salgado de Snyder 2021 | Impact of COVID-19 on Latinos from a SDOH perspective | Structural driver: Structural racism; Immigration policy; Residential segregation; Mediate driver: Occupational exposure (Essential work); Housing density; Linguistic barriers | Negative |
| Salvalaggio 2021 | Transplantation | Structural: National allocation policies; Mediate: Geography | Bidirectional |
| Sanogo 2024 | Social and clinical drivers of stress responses in breast cancer survivors | Structural driver: Structural racism; Healthcare access inequities; Systemic cancer disparities; Mediate driver: Clinical comorbidities (Hypertension); Sociodemographic factors; Cancer stage | Negative |
| Savage 2024 | Political determinants of health through advocacy training | Structural driver: Political polarization; Anti-science sentiment; State-level governance; Mediate driver: Advocacy preparedness; Legislative proposals; Cross-sector partnerships | Bidirectional |
| Schnake-Mahl 2020 | Impacts of gentrification and neighborhood change on population health | Structural driver: Gentrification; Urban development/regeneration; Housing policy; Mediate driver: Residential displacement; Neighborhood social capital; Built environment changes | Bidirectional |
| Serchen 2021 | Comprehensive policy framework for health equity and anti-discrimination | Structural driver: Structural racism; Institutional policies (Education/Criminal Justice); Workforce homogeneity; Mediate driver: Social drivers of health (SDOH); Implicit bias; Cultural humility; Maternal health access | Bidirectional |
| Shahidullah 2023 | Integrated primary care as a lever for child health equity | Structural driver: Structural racism; Socioeconomic inequity; Healthcare fragmentation; Mediate driver: Health-related social needs (HRSN); Integrated care models; Cultural humility | Bidirectional |
| Sharifi 2016 | Neighborhood characteristics and built environment in childhood obesity disparities | Structural driver: Urban planning/Segregation; Food environment (Fast food density); Mediate driver: Recreational space access; Neighborhood safety; Household SES | Negative |
| Socal 2020 | Institutional drivers of right-to-health litigation in Brazil. | Structural driver: Decentralized health system; Presence of Public Defender’s Office; Mediate driver: Legal/Administrative infrastructure; Pharmaceutical supply gaps | Bidirectional |
| Steinberg 2025 | Incarceration as a driver of racial disparities in MRSA infections. | Structural driver: Mass incarceration; Residential segregation; Structural racism; Mediate driver: Jail-community spillover; Detention conditions; Post-release instability | Negative |
| Taira 2019 | Social Emergency Medicine and health equity in clinical practice | Structural driver: Unequal resource distribution; Emergency healthcare system structure; Mediate driver: Patient-provider communication; Physician implicit bias; Health literacy | Bidirectional |
| Thornton 2023 | Investing in social determinants and antiracism to advance child health equity | Structural driver: Systemic racism; Social service investment; Housing/Environmental policies; Mediate driver: Health-related social needs (HRSN); Clinical screening; Integrated care models | Bidirectional |
| Thorpe 2025 | Intersection of racism, SDoH, and downstream biological pathways | Structural driver: Structural racism; Systemic segregation; Policy-driven disinvestment; Mediate driver: Allostatic load (weathering); Psychosocial stress; Neighborhood-level SDoH | Negative |
| Towe 2020 | Public mindsets and attitudes toward health equity drivers | Structural driver: Political ideology; Symbolic racism; Cultural narratives; Mediate driver: Perception of SDoH; Sense of community health; Individualistic health beliefs | Bidirectional |
| Toyobo 2022 | Perinatal health | Structural: Structural racism; Mediate: Implicit bias | Bidirectional |
| Trani 2025 | Dementia Risk | Structural: Structural racism; Mediate: Economic hardship | Negative |
| Trivedi 2022 | Healthcare disparities in Interventional Radiology | Structural driver: IR "deserts"; Specialty tribalism; Financial toxicity; Mediate driver: Physician implicit bias; Health literacy; Medical mistrust | Negative |
| Turan | COVID-19 Children | Structural Drivers (Upstream): Structural stigma and intersectionality (structural racism, sexism, and poverty) and legal/policy environments that criminalize or marginalize specific populations; Mediate Drivers: Biological stress pathways (HPA axis, cortisol, and chronic inflammation), mental health (depression/anxiety), and psychological resources (self-efficacy and resilience). | Negative |
| Uzzi 2025 | Opioid Overdose | Structural: Historical redlining; Mediate: Overdose risk | Negative |
| Van Doren 2023 | Health justice standards in graduate medical education | Structural driver: National medical education standards; Workforce homogeneity; Mediate driver: Resident knowledge of Structural drivers; Implicit bias; Advocacy interest | Bidirectional |
| Vance 2021 | Perinatal inequities | Structural: Systemic racism; Mediate: Provider diversity as positive driver | Bidirectional |
| Vaughan 2019 | County-level race-gender disparities in heart disease mortality | Structural driver: Geographic subnational inequalities; Systemic racism/sexism; Mediate driver: County-level SES; Healthcare access; Environmental stressors. | Negative |
| Wainstein 2025 | Kidney Disease Equity | Structural: Systemic health inequities; Health policies; Mediate: Social/Structural determinants as drivers | Bidirectional |
| Ward 2015 | CVD inequalities | Structural: Neighborhood SES; Mediate: Household income | Bidirectional |
| Wesson 2022 | Chronic Disease | Structural: Food/Activity Environments; Health Policy/Medicaid. Mediate: Lifestyle Behaviors (Diet/Exercise); Comorbidity Management | Bidirectional |
| WHO-PAHO 2019 | Health equity and social determinants in the Americas. | Structural driver: Colonialism; Structural racism; Governance and policy; Climate change; Mediate driver: Daily life conditions; Migrant status; Gender and ethnicity | Bidirectional |
| Williams 2021 | COVID-19 social drivers | Structural driver: Structural racism; Systemic health inequities; Residential segregation; Mediate driver: Comorbidities (syndemic interactions); Occupational exposure; Medical mistrust | Negative |
| Wilpers 2025 | Fetal Conditions | Structural driver: Structural racism; Inequities in specialized services; Health system policies; Mediate driver: Barriers to timely diagnosis; Counseling variations; Caregiver capacity | Negative |
| Xia 2022 | SARS-COV2 concentration | Structural driver: Visible minority status; Geographic heterogeneity; Urban inequality; Mediate driver: Essential work; Household density; Low income | Negative |
| Young 2025 | Critical pedagogy and strategies for screening and advocating for structural drivers in pediatric health | Structural driver: Political and historical influences; Medical education systems; Social accountability; Mediate driver: Community partnerships; Social and economic screening; Clinical advocacy | Bidirectional |
| Zheng 2022 | Heart Failure LOS | Structural driver: Hospital-level clustering; Institutionalized racism; Mediate driver: Discharge disposition; Insurance status. | Negative |
| Zirinsky 2021 | COVID-19 Children | Structural driver: Structural racism; Healthcare access inequities; Systemic segregation; Mediate driver: Household crowding; Parental occupational exposure; Digital divide. | Negative |

The "Mentioned or Interpreted Driver" category represents a synthesis of the causal pathways identified in the literature from the Americas. To provide analytical clarity, we distinguished between Structural drivers (the macro-level "engines" or institutional forces that generate stratification, such as systemic racism or policy frameworks) and Mediate Drivers (the intermediate "pathways" or facilitators, such as healthcare access or biological stress). When a study identified multiple factors, this classification was used to prioritize the primary upstream cause over downstream mechanisms, acknowledging an interpretive process aligned with established equity frameworks.

**Note: use of prompt of IA (Google Gemini):**

*I am providing you with a list of “drivers” and conceptual terms extracted and coded by the research team from a scoping review of 135 documents in the Americas.*

*Your task is to act as a technical assistant to refine and validate our preliminary classification into a two-level hierarchy (Structural and Mediate).*

*1. Structural Drivers: Identify which of my extracted terms function as macro-level “engines”, systemic forces like legal frameworks, economic policies, or historical legacies that dictate the distribution of power.*

*2. Mediate Drivers: Identify which terms function as “pathways”, intermediate manifestations like institutional behaviors, provider biases, or biological weathering that put those structural forces into motion.*

*Please do not generate new drivers; do not change the terms, only refine the naming and categorization of the items provided to ensure they reflect “kinetic force”' rather than static factors, thereby providing a functional bridge between SDoH theory and policy intervention.*

**Table S5.2. Methodological Strategies to Identify Health Equity Drivers (n=135)**

| Register | Study Category / General Design | Design | Methodology | Covariate Adjustment |
| --- | --- | --- | --- | --- |
| Adamkiewicz 2011 | Primary, quantitative | Cross-sectional | Secondary analysis | Multivariable analysis |
| Agarwal 2020 | Primary, quantitative | Cross-sectional | Survey and registry data | Multivariable analysis |
| Alkureishi 2021 | Primary, qualitative | Descriptive | Semi-structured interviews | NR |
| Bailey 2022 | Primary, quantitative | Cross-sectional | Survey | Multivariable analysis |
| Bailey 2023 | Primary, Descriptive | Perspective* | Narrative | NR |
| Balaji 2012 | Primary, qualitative | Exploratory | In-depth interviews | NR |
| Barber 2016 | Primary, quantitative | Cross-sectional | Secondary analysis | Multivariable analysis |
| Barber 2018 | Primary, quantitative | Cross-sectional | Public access registry | Multivariable analysis |
| Barnes 2022 | Opinion / Perspective | Perspective* | Evidence Synthesis | NR |
| Bedingfield 2022 | Primary, qualitative | Phenomenological | Semi-structured interviews | NR |
| Bhavsar 2024 | Methodological | Data Visualization | Visualization tools | NR |
| Brown TH 2022 | Primary, quantitative | Cross-sectional | Ecological analysis | Multivariable analysis |
| Caffe 2017 | Statement, Expert panel | Consensus | Expert opinion | NR |
| Campbell 2021 | Primary, quantitative | Cross-sectional | Survey and registry | Multivariable analysis |
| Chalasani 2022 | Narrative review | Literature review | Literature synthesis | NR |
| Chandra 2016 | Opinion / Perspective | Perspective* | Expert opinion review | NR |
| Coates 2020 | Statement, Expert panel | Policy analysis | Policy document review | NR |
| Cole 2023 | Narrative review | Literature review | Multi-method synthesis | NR |
| Correll-King 2025 | Primary, qualitative | Focus Groups | Qualitative interviews | NR |
| Credit 2020 | Primary, quantitative | Cross-sectional | Ecological analysis | Multivariable analysis |
| Cros 2019 | Primary, quantitative | Cross-sectional | Secondary analysis | Multivariable analysis |
| Dalsania 2020 | Primary, quantitative | Cross-sectional | Registry data analysis | Multivariable analysis |
| Darling-Hammond 2025 | Primary, quantitative | Cross-sectional | Regression analysis | Multivariable analysis |
| De Ornelas 2025 | Policy review | Thematic Analysis | Multi-method analysis | NR |
| Derose 2020 | Primary, qualitative | In-depth interviews | Semi-structured interviews | NR |
| Dharma 2025 | Primary, quantitative | Cross-sectional | Risk assessment | Multivariable analysis |
| DiPetrillo 2024 | Secondary, Scoping Review | Systematic Review | Systematic synthesis | NR |
| Ditah 2019 | Primary, quantitative | Cross-sectional | Secondary survey analysis | Multivariable analysis |
| Dove-Medows 2025 | Primary, qualitative | Ethnography | Narrative analysis | NR |
| Duchowny 2020 | Primary, quantitative | Cross-sectional | Secondary study data | Multivariable analysis |
| Dwyer-Lindgren 2017 | Primary, quantitative | Cross-sectional | Spatio-temporal analysis | Multivariable analysis |
| Egede 2022 | Narrative review | Literature review | Narrative synthesis | NR |
| English 2021 | Primary, quantitative | Cross-sectional | Survey analysis | Multivariable analysis |
| Estime 2022 | Narrative review | Implementation Review | Intervention analysis | NR |
| Evans 2023 | Statement, national report | Policy Report | Epidemiological data review | NR |
| Fene 2025 | Primary, quantitative | Cross-sectional | Multilevel analysis | Multivariable analysis |
| Figueroa 2020 | Primary, quantitative | Cross-sectional | Secondary study data | Multivariable analysis |
| Foryciarz 2025 | Methodological | Causal Modeling | Algorithmic Modeling | NR |
| Frakt 2020 | Opinion / Perspective | Perspective* | Literature review | NR |
| Friedman 2021 | Primary, quantitative | Cross-sectional | Time trend analysis | Arima model |
| Friedman J 2019 | Primary, quantitative | Cross-sectional | Time trend analysis | Arima model |
| Ghosh 2021 | Primary, quantitative | Retrospective cohort | Cohort analysis | Multivariable analysis |
| Gillman 2024 | Methodological | Epigenetic Analysis | Epigenomics review | NR |
| Glazer 2021 | Opinion / Perspective | Perspective* | Expert meeting review | NR |
| Golembeski 2021 | Opinion / Perspective | Perspective* | Literature synthesis | NR |
| Gompers 2025 | Primary, quantitative | Cross-sectional | Spatial regression | Multivariable analysis |
| Guilamo-Ramos 2020 | Opinion / Perspective | Perspective* | Evidence review | NR |
| Halbert 2023 | Secondary, narrative review | Literature review | Narrative synthesis | NR |
| Hamad 2025 | Secondary, narrative review | Policy Analysis | Review of policies | NR |
| Harper 2023 | Narrative review | Review | Implementation studies | NR |
| Harrison 2025 | Secondary, Methodological | Synthesis | Methodological Synthesis | NR |
| Hartinger 2024 | Secondary, Lancet report | Lancet Report | Evidence report | NR |
| Hatzenbuehler 2013 | Narrative review | Literature review | Theory review | NR |
| Hing 2024 | Secondary, scoping review | Systematic Review | Scoping review | NR |
| Holden 2022 | Primary, quantitative | Retrospective cohort | Cohort analysis | Matching |
| Huicho 2020 | Case studies | Multi-method | Policy/Survey analysis | NR |
| Hurwitz 2024 | Descriptive, narrative review | Literature review | Narrative review | NR |
| Ingraham 2021 | Primary, quantitative | Retrospective cohort | Cohort analysis | Multivariable analysis |
| Javed 2023 | Primary, quantitative | Cross-sectional | Secondary analysis | Multivariable analysis |
| Jean-Francois 2020 | Narrative review | Literature review | Synthesis | NR |
| Jenkinson 2024 | Primary, qualitative | Grounded Theory | Interviews | NR |
| Jernigan 2025 | Secondary, narrative review | Literature review | Review synthesis | NR |
| Ji Xu 2022 | Opinion / Perspective | Perspective* | Evidence synthesis | NR |
| Kavanagh 2021 | Primary, qualitative-quantitative | Mixed Methods | Structured questionnaire | NR |
| Keener Mast 2025 | Primary, qualitative | Implementation | Qualitative analysis | NR |
| Kegler 2022 | Case studies | Multi-method | Policy/Program analysis | NR |
| Kepper 2024 | Narrative review | Conceptual | Research review | NR |
| Kerr 2018 | Primary, quantitative | Cross-sectional | Survey analysis | Multivariable analysis |
| Klugman 2019 | Secondary, Evidence-based review | Meta-analysis | Evidence synthesis | Multivariable analysis |
| Kovach 2019 | Primary, qualitative | Delphi Method | Expert consensus | NR |
| Lacey 2023 | Narrative review | Literature review | Retrospective analysis | Multivariable analysis |
| Lavell 2023 | Primary, qualitative | Case Study | Qualitative analysis | NR |
| LeBrón 2025 | Primary, qualitative | Case Study | Qualitative case study | NR |
| Lee 2023 | Primary, qualitative | Descriptive | Semi-structured interviews | NR |
| Lewis 2025 | Primary, quantitative experimental | Experimental | ANOVA analysis | Factorial analysis |
| Little 2020 | Secondary, scoping review | Systematic Review | Scoping review | NR |
| Lobelo 2021 | Primary, quantitative | Retrospective cohort | Cohort analysis | Multivariable analysis |
| Loehrer AP 2023 | Secondary, Evidence-based review | Literature review | Evidence synthesis | NR |
| Martínez Leal 2023 | Primary, qualitative | Focus Groups | Focus group analysis | NR |
| Martínez-Cardoso 2020 | Narrative review | Literature review | Multi-method review | NR |
| Masters 2022 | Primary, quantitative | Cross-sectional | Survey analysis | Multivariable analysis |
| Mathews 2021 | Narrative review | Literature review | Synthesis | NR |
| Menza 2025 | Primary, quantitative | Cross-sectional | Regression analysis | Multivariable analysis |
| Mercado 2024 | Secondary, policy report | Policy Report | Report analysis | NR |
| Mikhail 2018 | Secondary, Evidence-based review | Literature review | Evidence synthesis | NR |
| Morey 2024 | Primary, Analytic Essay | Analytic Essay, Mixed methods | Descriptive | NR |
| Mourali 2025 | Primary, quantitative | Experimental | Quantitative analysis | Multivariable analysis |
| Mujahid 2023 | Primary, quantitative | Cross-sectional | Registry analysis | Multivariable analysis |
| Mujica 2023 | Primary, quantitative | Cross-sectional | Ecological analysis | Age-adjusted |
| Mustanski 2015 | Primary, quantitative | Cross-sectional | Survey analysis | Stratified analysis |
| Mustanski 2019 | Primary, quantitative | Cross-sectional | Cohort data analysis | Stratified analysis |
| Nelson 2025 | Primary, qualitative | Descriptive | Qualitative analysis | NR |
| Nielsen 2024 | Review, Conceptual | Perspective* | Geroscience review | NR |
| Noaeen 2025 | Primary, quantitative | Cross-sectional | Causal modeling | Multivariable analysis |
| Noel 2024 | Primary, case study | Case Study | Case study analysis | NR |
| Odufalu 2022 | Narrative review | Literature review | Synthesis | NR |
| Parra Moreno 2025 | Primary, quantitative | Cross-sectional | Critical analysis | Multivariable analysis |
| Petiwala 2021 | Secondary, Evidence-based review | Literature review | Document analysis | NR |
| Philbin 2018 | Narrative review | Literature review | Policy review | NR |
| Pinheiro de Souza 2020 | Primary, qualitative | Pedagogical Analysis | Semi-structured interviews | NR |
| Quinn 2024 | Primary, qualitative | Descriptive | Qualitative analysis | NR |
| Razdan 2024 | Primary, qualitative | Grounded Theory | Qualitative analysis | NR |
| Saigí 2021 | Narrative review | Literature review | TAM analysis | NR |
| Salgado 2021 | Secondary, Evidence-based review | Literature review | Evidence synthesis | NR |
| Salvalaggio 2021 | Narrative review | Literature review | Policy synthesis | NR |
| Sanogo 2024 | Primary, quantitative | Cross-sectional | Logistic regression | Multivariable analysis |
| Savage 2024 | Primary, Descriptive | Policy Review | Roadmap analysis | NR |
| Schnake-Mahl 2020 | Secondary, Evidence-based review | Literature review | Quantitative synthesis | NR |
| Serchen 2021 | Statement, national report | Policy Review | Comprehensive review | NR |
| Shahidullah 2023 | Opinion / Perspective | Perspective* | Evidence review | NR |
| Sharifi 2016 | Primary, quantitative | Cross-sectional | Clinical records analysis | Multivariable analysis |
| Socal 2020 | Case studies | Multi-method | Policy/Legal analysis | NR |
| Steinberg 2025 | Primary, quantitative | Cross-sectional | Regression analysis | Multivariable analysis |
| Taira 2019 | Narrative review | Literature review | Synthesis | NR |
| Thornton 2023 | Narrative review | Literature review | Synthesis | NR |
| Thorpe 2025 | Secondary, narrative review | Literature review | Pathway review | NR |
| Towe 2020 | Primary, quantitative | Cross-sectional | Survey analysis | Multivariable analysis |
| Toyobo 2022 | Narrative review | Systematic review | Systematic synthesis | NR |
| Trani 2025 | Primary, qualitative | Systems Dynamics | CBSD analysis | NR |
| Trivedi 2022 | Narrative review | Literature review | Literature synthesis | NR |
| Turan 2017 | Narrative review | Conceptual analysis and theoretical synthesis | Narrative review | NR |
| Uzzi 2025 | Primary, quantitative | Cross-sectional | Geospatial analysis | Multivariable analysis |
| Van Doren 2023 | Primary, qualitative | Evaluation | Discussion rounds | NR |
| Vance 2021 | Opinion / Perspective | Perspective* | Narrative summary | NR |
| Vaughan 2019 | Primary, quantitative | Cross-sectional | Aggregate data analysis | Stratified analysis |
| Wainstein 2024 | Descriptive, policy analysis | Narrative | Advocacy analysis | NR |
| Ward 2015 | Primary, quantitative | Cross-sectional | Survey analysis | Multivariable analysis |
| Wesson 2022 | Narrative review | Literature review | Synthesis | NR |
| WHO-PAHO 2019. | Statement, regional report | PAHO Report | Epidemiological review | NR |
| Williams 2021 | Opinion / Perspective | Perspective* | Syndemic evaluation | NR |
| Wilpers 2025 | Secondary, narrative review | Perspective* | Conceptual review | NR |
| Xia 2022 | Primary, quantitative | Cross-sectional | Gini covariance analysis | SDOH coefficients |
| Young 2025 | Secondary, narrative review | Perspective* | Pedagogical essay | NR |
| Zheng 2022 | Primary, quantitative | Cross-sectional | Hierarchical modeling | Multivariable analysis |
| Zirinsky 2021 | Narrative review | Literature review | Epidemiological review | NR |

*	They were included for the evidence they contained

**Table S5.3. Research Gaps (identified & interpreted) (n=105)**

| Author | Topic | Research Gaps |
| --- | --- | --- |
| Adamkiewicz 2011 | Indoor environment | Describes socioeconomic inequalities in indoor exposures. Gaps include further examination of individual pollutants and characterizing root causes through community participation to link housing with health. |
| Agarwal 2020 | Racial-ethnic inequity in glycemic control and technology use among young adults with Type 1 Diabetes | Highlights that studies on minority populations with T1D are scarce. Research fails to examine co-occurring social determinants and lacks direct comparisons between Hispanic and African American groups. |
| Alkureishi 2021 | Digital divide | Suggests qualitative research to understand patient perspectives on digital equity and community-based programs for technological integration in healthcare. |
| Bailey 2022 | Health inequality | Points to a gap in longitudinal data regarding intergenerational trauma caused by structural racism and its biological "weathering" effects. |
| Bailey 2023 | Transition to care for youth with chronic conditions | Identifies a need for qualitative research capturing the lived experience of patients facing structural violence during the transition to adult care. |
| Balaji 2012 | HIV and MSM | Highlights the lack of culturally tailored interventions addressing how social norms of masculinity drive HIV risk in immigrant communities. |
| Barber 2018 | Structural racism | Identifies a gap in understanding how municipal-level policies and residential segregation mitigate or exacerbate the health effects of structural racism. |
| Bhavsar 2024 | Data Visualization | Highlights the silence regarding algorithmic bias as a structural determinant and the need for transparent data visualization in healthcare access. |
| Brown TH 2022 | Racial inequities in COVID-19 mortality at the state level. | Notes a gap in lifecourse research regarding how early-life exposure to discrimination and structural inequality predicts morbidity during the pandemic. |
| Campbell 2021 | Glycemic control among inner-city African Americans | Notes the lack of data on how immigration status and structural barriers in primary care specifically affect glycemic control in minority populations. |
| Chandra 2016 | Shared values on health | Identifies a gap in research comparing ethnic groups to guide intervention approaches based on health as a shared value and civic engagement. |
| Correll-King 2025 | Firearm violence | Identifies a need for community-led research on the impact of Structural drivers and police presence on mental health in high-violence neighborhoods. |
| Cros 2019 | Health in Haiti | Highlights a gap in research on how social support networks and structural poverty interact to affect child development and health in Haiti. |
| Dalsania 2020 | COVID-19 racism | Points to a lack of data on the role of structural racism and housing instability in driving rural-urban disparities during the pandemic. |
| Darling-Hammond 2025 | Racial Identity | Identifies a gap in research on how educational policies and structural racial identity act as "upstream" determinants of cardiovascular health. |
| De Ornelas 2025 | Racial inequities in maternal health | Notes the lack of policy evaluation tools that account for the intersection of climate change and social vulnerability in maternal health. |
| Derose 2020 | Food insecurity | Highlights the lack of trust in healthcare institutions as a modifiable Structural driver requiring systemic interventions to improve food security. |
| Dharma 2025 | Psychiatric Bias | Points to a gap in risk-assessment models that include environmental toxins and structural bias as drivers of inequity in psychiatric care. |
| DiPetrillo 2024 | Anti-racism | Research is needed that actively searches for grey and non‑English literature, applies mixed‑methods and qualitative approaches, develops comparative frameworks for heterogeneous interventions, and explores structural interventions addressing racism that are not reflected in the academic literature |
| Dove-Medows 2025 | Obstetric Racism | Highlights the silence on the lived experience of structural racism and "medical gaslighting" among Black pregnant women in clinical settings. |
| Duchowny 2020 | Life course / strength | Notes a gap in research regarding the cumulative impact of social determinants on physical frailty among aging minorities over the lifecourse. |
| Dwyer-Lindgren 2017 | Life expectancy | Emphasizes the need to understand why geographic disparities in life expectancy are widening at the county level despite national improvements. |
| Egede 2022 | Diabetes care | Identifies a need for implementation science to move beyond description toward intersectoral and structural solutions for diabetes disparities. |
| English 2021 | LGBTQ policy | Points to a gap in research on how state-level policies and systemic police violence impact the mental health of Black LGBTQ youth. |
| Estime 2022 | Trauma patients | Highlights the need for intervention studies addressing the intersection of food insecurity, structural poverty, and recovery in trauma patients. |
| Evans 2023 | Inequity leadership | Identifies a lack of coordination between health system leadership and community-led initiatives in addressing structural inequities. |
| Fene 2025 | Suboptimal child vaccination | Notes a gap in multilevel research on how subnational geographic inequalities and structural barriers impact vaccination rates. |
| Figueroa 2020 | Racial/ethnic disparities in COVID-19 cases and deaths across U.S. counties | More research is needed to understand the key drivers of higher COVID-19 case and death rates in Afro‑descendant communities. |
| Foryciarz 2025 | Social drivers in chronic kidney disease algorithms | Identifies a total silence on "Algorithmic Justice" in LAC and the need for transparency in how automated systems impact healthcare access. |
| Frakt 2020 | Medical Debt | Points to the need for health systems to take financial responsibility for addressing the social and structural needs that lead to medical debt. |
| Friedman 2021 | Overdose mortality | Absence of evidence regarding the health impact of the carceral state and structural violence on overdose mortality in vulnerable populations. |
| Ghosh 2021 | Race, SES and LOS | Highlights the need for research on the role of community health workers in bridging the digital divide for elderly minorities. |
| Gillman 2024 | Epigenetics | Identifies a gap in epigenetics research regarding the molecular markers of social adversity and structural inequality across generations. |
| Golembeski 2021 | Carceral crises | Identifies a gap in research regarding health outcomes and structural barriers faced by individuals transitioning from the justice system to the community. |
| Gompers 2025 | Disparities in Stroke Mortality | Future research should utilize smaller, more granular geographic units and longitudinal data to overcome the limitations of cross-sectional ecological analysis and potential masking of sub-county variations. Furthermore, there is a critical need to further explore the intersectional mechanisms through which structural racism and sexism jointly impact cardiovascular health across diverse minoritized populations |
| Halbert 2023 | AA Men Cancer | Future research must integrate longitudinal data on stress exposure with biological markers to determine the precise timing and impact of socio-environmental stressors on cancer progression. Furthermore, there is a critical need to evaluate how multilevel interventions targeting both social needs and biological resilience can mitigate these persistent health inequities |
| Hamad 2025 | Economic Policy | Suggests that policies increasing income improve health, but research on the long-term health impacts of specific policies like minimum wage remains insufficient. |
| Harper 2023 | Ethnic disparities in pulmonary diseases | Future research must shift from descriptive epidemiology toward testing structural and multi-level interventions in pulmonary diseases that explicitly target the root causes of disparity. Furthermore, there is a critical need to investigate how telehealth and precision medicine can be leveraged to improve outcomes in marginalized communities without reinforcing existing digital and social divides |
| Harrison 2025 | Infant Mortality | Notes a lack of societal-level proxies for infant mortality that go beyond individual clinical risks to address structural conditions. |
| Hartinger 2024 | Climate Change LAC | Highlights the urgent need for a "One Health" approach in the Americas linking environmental health, climate change, and social justice. |
| Hatzenbuehler 2013 | Stigma | Identifies a gap in longitudinal research on the impact of structural stigma (laws and policies) on LGBTQ+ mental health. |
| Hing 2024 | Measuring Racism | Notes a lack of standardized tools focusing on the Structural drivers of disability-based health inequities. |
| Holden 2022 | COVID-19 racism | Highlights the need for better data on how insurance status and structural racism interact to determine access to specialty care. |
| Huicho 2020 | Stunting reduction | Identifies a gap in "how-to" implementation science for pediatric care and emphasizes the need for subnational data on Indigenous health. |
| Hurwitz 2024 | Cardio Intersection | Notes a lack of research on the role of the built environment and Structural drivers in promoting cardiovascular resilience in urban areas. |
| Ingraham 2021 | Language / Neighborhood | There is a pressing need for research that uncovers the mechanisms (including structural racism, testing/access inequities, and language barriers) driving racial and ethnic disparities in COVID‑19 severity; uses individual‑level socioeconomic data and mixed‑methods/community‑engaged approaches; and incorporates non‑English and grey literature to capture interventions and contexts not reflected in academic studies |
| Javed 2023 | CVD mortality | The authors call for studying associations within cumulative disadvantage rather than isolated socioeconomic measures, to uncover how social determinants interact -including structural racism- to drive higher mortality. They recommend multilevel modeling and mixed‑methods research to disentangle the interplay of sex, race, discrimination, and mortality, and to fill gaps like lacking reliable data on subjective experiences of racism not captured by large quantitative surveys. |
| Jean-Francois 2020 | Health IT tools and disparities in maternal morbidity and mortality | Research is needed on multilevel interventions that leverage health IT tools to address disparities throughout the continuum of maternity care to ensure that all women can benefit from an evidence-informed U.S. maternity care system. |
| Jenkinson 2024 | Homelessness | Need for strategies to rebuild institutional trust in marginalized groups. |
| Jernigan 2025 | Indigenous Health | Notes the lack of Indigenous-led frameworks in the study of food sovereignty and structural health equity in tribal nations. |
| Ji Xu 2022 | Racial/ethnic disparities in childhood cancer survival | Identifying modifiable drivers of racial/ethnic disparities in childhood cancer is vital to inform policy and to develop interventions for sustained reduction of inequities. Future research should focus on combinations of interventions targeting modifiable drivers at multiple levels, including families, the healthcare system, the community, and society. |
| Kavanagh 2021 | National health plans in the Americas regarding health equity | There is a need to identify which policies most effectively reduce health inequalities at scale, and to establish baseline national data and legal/policy reviews to track progress-particularly for marginalized groups (Afro‑descendant, Indigenous, migrants). |
| Keener 2025 | COVID-19 prevention in K-12 schools | Highlights the lack of qualitative evidence on the "burden of navigation" for families in complex school and health systems. Need for school-specific implementation frameworks for public health. |
| Kepper 2024 | Digital Health | Need for rigorous evaluation of multilevel digital strategies and causal tests for underserved groups |
| Kerr 2018 | Neighborhood stigma | It calls for more research, primarily longitudinal studies, to examine whether social marginalization in one domain (neighborhood quality) increases HIV‑related stigma; the possible pathways that facilitate this relationship; and the socio‑environmental factors that influence it. |
| Klugman 2019 | Lung cancer | Comorbidities, social support measures (including marital status), and especially smoking (ideally using pack‑years data to avoid residual confounding) should be included as covariates in future analyses, since this review found an association with mortality. |
| Kovach 2019 | Equity strategy | To identify opportunities to guide the American Academy of Family Physicians’ health‑equity strategy, focusing on four domains: leadership/policy/research/diversity and decision context. Thirteen research topics were prioritized; key priorities include: which multilevel interventions effectively improve health equity; the impacts of policies and governance; translation and dissemination of equity interventions; and how to address the health effects of racism and discrimination. |
| Lacey 2023 | SDOH on adult trauma outcomes | A significant research gap exists in evaluating hospital-based and regional interventions to mitigate trauma disparities. Future research must shift from descriptive epidemiology to testing structural changes in trauma systems. Additionally, longitudinal data on long-term functional recovery among Afro-descendant populations is critically needed to inform rehabilitation policies. |
| Lavell 2023 | Disaster risk and COVID-19 in Lima, Peru | Gaps in understanding how informal urban settlement drivers interact with pandemic risk. |
| LeBrón 2025 | Post-pandemic "benefits cliff" and the role of community-based organizations | Need for sustainable funding models that address long-term structural inequities post-crisis (Covid-19) |
| Lewis 2025 | Structural messaging on COVID-19 disparity perceptions and advocacy | Identifies a gap in experimental research on how Structural drivers of disparities interact with messaging effects in public health communication. |
| Little 2020 | Dietary transition and health implications among Canadian Inuit | Key gaps include the scarcity of longitudinal studies to establish causal links between dietary shifts and health, limited Inuit‑led and participatory research aligned with community priorities, and inadequate evaluation of community‑led interventions. Research is also lacking on structural/economic drivers (food environments), comprehensive risk-benefit analyses (nutrition, contaminants, cultural and mental health), standardized measurement methods, climate‑change impacts on food access, and age/gender differences and subjective cultural experiences. |
| Martínez-Cardoso 2020 | Diabetes management and control among US immigrants | More research is needed to understand the true extent of diabetes among migrants. Many studies document the harmful health effects of anti‑immigration policies and deportation practices, but none have examined diabetes care and control outcomes in migrant populations. The authors recommend investigating precarious work, shift work, the informal economy, and how these employment conditions interrelate. |
| Mathews 2021 | Disparities in Cardiac Rehabilitation | Notes the invisibility of gender-nonconforming individuals in research on structural barriers to cardiovascular rehabilitation. |
| Menza 2025 | Multi-level drivers of congenital syphilis | Key research gaps: incomplete individual‑level data and high missingness limit analysis of drivers like housing and substance use; need for longitudinal and finer‑scale (census‑tract) studies to assess causal pathways; lack of qualitative/mixed‑methods work to explain barriers to prenatal care and treatment; limited generalizability beyond Oregon requiring replication in other jurisdictions; and insufficient evaluation of interventions and care models to prevent congenital syphilis, and a need for targeted implementation and evaluation research in carceral settings. |
| Mercado 2024 | The Societal Structural and Systemic Drivers (SSD) model | Key gaps include the lack of empirical, longitudinal evidence quantifying how specific structural and systemic drivers (SSD) causally generate SDOH and health inequities across the life course and generations. There is a need for standardized indicators and multilevel methods to measure and compare SSD dimensions and their interactions. Research must integrate nontraditional data sources and advanced modeling to capture complex, cross‑domain effects and policy impacts. Participatory, community‑led studies are required to align priorities and assess culturally appropriate structural interventions. Finally, rigorous evaluations of policy and systems reforms are needed to identify effective strategies for reducing SSD‑driven health disparities. |
| Mikhail 2018 | Socioecological framework of the social determinants of trauma disparities. | The article identifies five priority research areas: improving patient–provider communication; promoting community engagement and outreach through technology; enhancing care in centers serving higher proportions of minority patients; evaluating the long‑term effects of acute intervention and rehabilitation support; and improving patient‑centred care by identifying recovery expectations. |
| Morey 2024 | Democratizing data for Native Hawaiians and Pacific Islanders communities to achieve health and social equity | Federal NHPI data are often unavailable or aggregated, leaving subgroups invisible and hindering targeted action. There is limited evidence on how to make data salient and usable for NHPI communities, including best practices for community report‑back. High access costs and restricted microdata block community use and evaluation of local inequities. Few studies evaluate the impact of democratized data or capacity‑building interventions on NHPI‑led programs and policy advocacy. Research is needed on ethical frameworks and safeguards to increase data access while preventing misuse and harm. |
| Mourali 2025 | AI/Algorithmic decision-making | The study’s ecological validity is limited by its use of convenience sampling and hypothetical preferences. Future research should prioritize field experiments with affected populations and account for geographic and contextual variables. Furthermore, the lack of evidence on how prior experience with bias impacts trust necessitates interdisciplinary approaches to guide the governance of fairness-accuracy trade-offs. |
| Mujahid 2023 | Neighborhood deprivation and severe maternal morbidity | Future research should examine associations at finer geographic scales to uncover local risk differences masked at the county or ZIP‑code level. Clinical databases should use patient address data to enable neighborhood‑level geocoding. Given persistent racial and ethnic inequities in severe maternal morbidity, more research is needed to expand measurement of harmful and protective neighborhood‑level factors that may be particularly salient for marginalized racial and ethnic populations and to assess their effects on severe maternal morbidity. |
| Mujica 2023 | Income-related inequalities in violent deaths among youth in the Americas | Further investigation is needed to confirm inequality patterns at the individual level and to explore underlying mechanisms, age- and sex-specific vulnerabilities, and gender-based drivers of these inequalities. Likewise, countries must look beyond national averages to examine inequalities at the local level and act on social determinants by repeatedly analyzing disaggregated data through equity-based measurements |
| Mustanski 2015 | Geographic and network drivers of HIV disparities among YMSM. | Identifies a gap in research on the intersection of sexual orientation, racial discrimination, and structural barriers to HIV prevention. |
| Nelson 2025 | Structural inequities and provider burnout in US maternal health | Gaps in understanding how provider burnout acts as a secondary driver of maternal mortality. |
| Nielsen 2024 | Biological aging (Geroscience) | Need for cross-disciplinary metrics linking social drivers to specific biological clocks. |
| Noaeen 2025 | Neighborhood-level drivers of Type 2 Diabetes | Highlights the gap in predictive-causal models linking neighborhood-level Structural drivers with individual Type 2 Diabetes onset. |
| Noel 2024 | Equitable transitions of cancer care | There is a critical knowledge gap regarding how to successfully translate academic research and clinical oncology protocols into sustainable, real-world community practice. Specifically, they identify a lack of research on inclusive strategies that integrate community voices into the implementation process, as most studies focus on what works "on paper" rather than in practice. Furthermore, the study highlights a disconnect between stakeholders, noting that future research must address varying perspectives on upstream versus downstream social drivers and the specific need for "caregiver navigation" as opposed to standard patient navigation. |
| Parra Moreno 2025 | Ontological insecurity and structural inequality as drivers of migration | Notes a lack of critical analysis of how privatized health systems in LAC exacerbate socioeconomic disparities and ontological insecurity. |
| Petiwala 2021 | Sector collaboration | Identifies a gap in institutional power sharing and the need to prioritize community voice in cross-sector health collaborations. |
| Philbin 2018 | State-level immigration policies as drivers of health disparities | Research on the impact of state policies on Latino health is vital as it explores how modifiable structural variables influence vulnerable populations. The most pressing question is the extent to which supportive state or municipal policy contexts can buffer the intense xenophobia characterizing the current U.S. federal landscape. This line of inquiry could identify how structural racism and state-level policies impact Latino health, ultimately promote inclusive, health-promoting policies and fostering cross-sector collaboration among advocates in immigration, education, social justice, and public health |
| Pinhero de Souza 2020 | Social participation | Identifies a gap in understanding how academic engagement and institutional social control influence health participation in Brazil. |
| Quinn 2024 | Anti-Black racism, mental health, and HIV care outcomes | Highlights the gap in research on anti-Black racism and structural violence as primary drivers within the HIV care continuum. |
| Razdan 2024 | Exploration of NICU disparities and the role of equity dashboard | Current health equity dashboards in the NICU are limited by their reliance on clinical metrics, which fail to capture interpersonal bias, institutional barriers, and social determinants. Future research and dashboard iterations require novel, qualitative data sources to identify these underlying etiologies and move beyond basic quality improvement metrics to address the root causes of neonatal disparities. |
| Salgado de Snyder 2021 | COVID-19 on Latinos from a SDOH perspective | Current research is limited by a heavy reliance on existing national databases that omit variables critical to the Latino experience. There is a pressing gap in studies grounded in deep cultural and social understanding, specifically regarding drivers like generational status and national origin. Addressing these omissions is essential to identify high-risk subgroups and implement targeted policies that reduce disparities in health access and mortality. |
| Sanogo 2024 | Stress responses in breast cancer survivors | There is a lack of large-scale prospective studies among African American women with breast cancer to evaluate diurnal cortisol patterns (e.g., cortisol awakening response and diurnal slope) and their long-term effects on survival. Additionally, research is needed to investigate the mechanisms and downstream consequences of elevated cortisol, using multiple measurements over time and more diverse samples to strengthen statistical power and generalizability. |
| Savage 2024 | Political determinants of health through advocacy training | It is the lack of pedagogical models or strategies to prepare professionals and citizens for effective advocacy on the political determinants of health |
| Schnake-Mahl 2020 | Gentrification and neighborhood change on population health | Despite extensive research on neighborhood effects, how neighborhood economic development via gentrification impacts health and well-being in the USA remains poorly understood, with no prior systematic assessment of its potential health consequences. There is a critical empirical gap regarding the causal mechanisms at play and whether health impacts differ across various forms of neighborhood socioeconomic ascent. Furthermore, public health has remained on the periphery of these debates, highlighting the need for research that addresses subpopulation effects and the full range of implications for population health |
| Serchen 2021 | Comprehensive policy framework for health equity and anti-discrimination | The American College of Physicians identifies a significant research gap in the lack of an interconnected framework that addresses health disparities across multiple societal sectors. Specifically, the authors report a critical need for evidence-based research to identify best practices for mitigating structural racism within clinical settings and for more granular data that accurately captures the lived realities of marginalized populations. Furthermore, a gap remains in recognizing criminal justice policies as fundamental public health issues that require systemic, health-focused reforms |
| Shahidullah 2023 | Integrated primary care as a lever for child health equity | Need for more research on large-scale implementation of integrated models, the impact of telemedicine on child equity, and the effectiveness of interventions targeting structural racism in clinical settings. |
| Sharifi 2016 | Neighborhood characteristics and built environment in childhood obesity disparities | Lack of large-scale studies that utilize objective BMI measures, longitudinal data, and a comprehensive range of Geographic Information System (GIS) metrics. There is a limited understanding of how neighborhood-level factors -beyond individual behaviors- mediate racial and ethnic disparities in childhood obesity, highlighting the need for research that investigates how to tailor clinical and public health interventions to the specific contexts of the built environment. |
| Steinberg 2025 | Incarceration as a driver of racial disparities in MRSA infections. | The authors report several research gaps, specifically the need for more granular data to characterize the mechanistic link between public transit utilization and MRSA transmission. Additionally, there is a lack of sub-county level data on key risk factors such as injection drug use and sex work, which limits the ability to assess their confounding role in the relationship between incarceration and CA-MRSA. Finally, the authors highlight the difficulty in disentangling the specific impacts of jail cycling versus state prison releases on community infection rates |
| Thornton 2023 | Investing in social determinants and antiracism to advance child health equity | Critical evidence gap regarding the empirical effectiveness of healthcare-based interventions in producing sustained reductions in child health disparities. While social needs screening has increased, research often fails to address structural racism and the legal or policy-level drivers of inequity. There is a pressing need for studies that evaluate multi-sector collaborations and 'medical-legal partnerships' to move beyond individual-level social assistance toward structural change |
| Thorpe 2025 | Intersection of racism, SDoH, and downstream biological pathways | The authors report a critical gap in the lack of interdisciplinary integration between social sciences and traditional biological or medical domains. There is a limited understanding of the specific neurocognitive and molecular pathways -such as inflammatory cytokines and stress-responsive signaling -that link structural racism to biological weathering. Future research must address how race-based discriminatory policies culminate in downstream physiological damage to develop multi-level interventions targeting these root causes. |
| Toyobo 2022 | Perinatal health | Current research in obstetric anesthesia has extensively documented racial and ethnic disparities in perinatal outcomes, yet a critical gap remains in understanding the specific causal mechanisms and structural drivers behind these inequities. There is an urgent need to transition from descriptive studies to hypothesis-driven research that tests targeted interventions and identifies actionable levers within the health system. Furthermore, the lack of standardized, validated metrics to evaluate equity in anesthesia care processes hinders the development of evidence-based countermeasures to eliminate these persistent health divides. |
| Trani 2025 | Dementia Risk | Although this study identifies critical structural pathways for dementia risk in St. Louis, a significant research gap remains regarding the generalizability of these community-derived models to other Black populations across the United States. Future research must address the limited representation of Black men and individuals with lower educational attainment to ensure the perspectives of all community segments are captured. Additionally, there is a need to further investigate how individual socioeconomic characteristics, beyond geographic location, specifically shape the perceived salience of biological versus environmental factors in the etiology of dementia |
| Turan 2017 | Stigma as a driver of HIV-related health | Future research must utilize comprehensive, theory-driven frameworks to investigate the intersectional effects of structural racism and stigma on treatment adherence and physiological health. Furthermore, there is a critical need for studies that move beyond descriptive associations to evaluate interventions that simultaneously address psychological mediators and the biological 'embodiment' of stigma |
| Uzzi 2025 | Opioid Overdose | While this study establishes a longitudinal link between structural racism and fatal overdoses in Chicago, a research gap remains in uncovering the precise mechanistic pathways connecting these neighborhood environments to drug mortality. Future studies must incorporate post-pandemic data to evaluate long-term trends and investigate the specific types of drugs present in highly segregated areas. Additionally, research is needed to differentiate the structural impacts on fatal overdoses among individuals with Opioid Use Disorder versus those exposed to fentanyl-adulterated supplies without a prior diagnosis |
| Vance 2021 | Perinatal inequities | A significant research gap persists in shifting the focus from biological or racialized explanations toward the social determinants of health as primary drivers of preterm birth. Future studies and quality improvement efforts must investigate the effectiveness of anti-racist strategies and implicit bias training in directly reducing health inequities. Furthermore, there is a profound need for research that evaluates systemic solutions to increase diversity and foster inclusive environments within neonatal care units |
| Vaughan 2019 | County-level race-gender disparities in heart disease mortality | Although this study identifies significant geographic variations in race-gender mortality patterns, a critical research gap remains in testing the specific social and environmental hypotheses that drive these rate inversions at the county level. Future research is needed to utilize long-term cohort studies or robust surveillance systems to overcome the limitations of national vital statistics, which lack consistent individual-level socio-demographic data over time. Furthermore, investigating the 'weathering' process through physiological markers like allostatic load is essential to understand why structural drivers override biological protection in younger Black women. |
| Wainstein 2025 | Kidney Disease Equity | There is a critical need for advocacy-informed research that quantifies the scope of unrecognized health inequities, particularly in wealthy settings where disparities in access to specialized care often remain invisible. Furthermore, future studies must bridge the gap between humanistic experiences and formal evidence to catalyze sustainable reforms in global health policies |
| Ward 2015 | CVD inequalities | While this study highlights shifting social gradients in an urban Brazilian context, a critical research gap remains in establishing the longitudinal causal interrelations between education, household income, and neighborhood-level factors. Future research must utilize longitudinal designs to investigate the sequencing of these socioeconomic variables and include more nuanced measures such as occupation and specific neighborhood infrastructure. Additionally, further analysis is needed to understand the mechanistic drivers behind gender-specific body image perceptions and their role in cardiovascular risk across diverse Latin American settings |
| Wilpers 2025 | Fetal Conditions | Highlights the need for models integrating contextual, structural, and process-level factors influencing care and outcomes in fetal conditions. |
| Xia 2022 | SARS-COV2 concentration | This study identifies a strong geographic concentration of SARS-CoV-2 cases linked to social determinants across Canadian cities. A research gap remains in understanding the temporal evolution of these inequalities throughout different pandemic waves. Future research should integrate individual-level data to disentangle the overlapping effects of housing, occupation, and income on transmission dynamics. Additionally, there is a need to evaluate the effectiveness of community-specific mitigation strategies that are directly responsive to the unique structural drivers identified in each metropolitan area |
| Young 2025 | Critical pedagogy and strategies for screening and advocating for structural drivers in pediatric health | The importance of structural competency is increasingly recognized in medical education. A research gap remains in the lack of a standardized approach to curriculum development and the assessment of long-term educational outcomes. There is a need to investigate how integrating critical pedagogy across all four years of medical school specifically enhances a clinician's ability to transition from individual screening to effective community organizing and policy advocacy. Furthermore, research is needed to evaluate the impact of theory-driven shifts,such as institutionalizing anti-racism, on reducing the clinical 'mindset gap' regarding public health responsibilities |
| Zheng 2022 | Heart Failure LOS | Research gap exists in identifying the specific mechanisms of differential treatment within hospital settings. There is a need for studies that disentangle the impact of structural drivers, such as institutional bias and resource allocation, from individual social determinants like post-discharge support. Future research must investigate how these overlapping factors contribute to healthcare inequities to develop targeted interventions that ensure standardized, high-quality care for all racial and ethnic groups. |
| Zirinsky 2021 | COVID-19 Children | A critical research gap remains in understanding the long-term sequelae of the virus in pediatric populations. There is a need for data-driven strategies to improve vaccine acceptability and clinical trial representation for racial and ethnic minorities to ensure equitable access to future therapeutics. Furthermore, the lack of robust viral surveillance in underserved communities hinders the ability to monitor viral replacement and maintain long-term vaccine effectiveness |

**Table S5.4. Theoretical and Conceptual Frameworks or Models (FTM) identified in the Americas***

| Study | Topic | Used FTM N=124/135 | Develop new FTM N=26/135 |
| --- | --- | --- | --- |
| Adamkiewicz 2011 | Indoor environmental exposure |  | Indoor Environmental Exposure Drivers Framework |
| Agarwal 2020 | Racial disparities in T1D | WHO/CSDH Framework |  |
| Alkureishi 2021 | Digital Divide |  | Digital Divide Patient-Centered Model |
| Bailey 2022 | Health Inequalities |  | Structural Racism & Health Equity Framework |
| Bailey 2023 | Transition to adult care | Fundamental Cause Theory | Cumulative Disadvantage Framework |
| Barnes 2022 | Alzheimer's and racism | Social Determinants of Health (SDoH) |  |
| Bedingfield 2022 | Tuberculosis | Ecosocial Theory |  |
| Bhavsar 2024 | Data Visualization | SEED Framework |  |
| Brown TH 2022 | Racial inequity in COVID-19 | Racial Health Equity Framework |  |
| Campbell 2021 | Glycemic control in AA | Multilevel Barriers to Care (Campbell & Egede) |  |
| Chalasani 2022 | Medication access |  | Pharmacoequity Path Framework |
| Chandra 2016 | Shared values on health | Robert Wood Johnson Foundation’s Culture of Health Action Framework |  |
| Coates 2020 | Equity in national health plans | Framework of the Commission on Equity and Health Inequalities in the Americas |  |
| Cole 2023 | Gentrification |  | Gentrification–Health Pathways Framework |
| Correll-King 2025 | Firearm violence LGBTQ+ | Intersectional Racism Framework |  |
| Credit 2020 | Geographic location and COVID-19 | SDOH |  |
| Cros 2019 | Health provision in Haiti | SDG Framework |  |
| Dalsania 2020 | COVID-19 and structural racism | Structural Racism Framework |  |
| Darling-Hammond 2025 | Racial Identity & Suicide | Social stress theory |  |
| De Ornelas 2025 | Maternal Health / Inequity | MMRIA-RD Framework |  |
| Derose 2020 | Food insecurity | Determinants influencing food insecurity and HIV linkages (Weiser multi-level framework) | Expanded Weiser multi-level framework |
| Dharma 2025 | Psychiatric Bias | Social Determinants of Health (SDoH) |  |
| DiPetrillo 2024 | Anti-racism interventions | HEART Framework; Socio-Ecological Model |  |
| Ditah 2019 | Age at HF diagnosis | Social Determinants of Health (SDoH) |  |
| Duchowny 2020 | Life course and muscle strength | Life Course Perspective; Cumulative Disadvantage |  |
| Dwyer-Lindgren 2017 | Life expectancy inequities | Social Determinants of Health (SDoH) |  |
| Egede 2022 | Diabetes and intersectoriality | Chronic Care Model (CCM); NIMHD Framework; RWJF Framework |  |
| English 2021 | Structural racism and LGBTQ | Intersectionality; Minority Stress |  |
| Estime 2022 | Disparities in trauma patients | Social Determinants of Health (SDoH); Quality Improvement (QI) |  |
| Evans 2023 | Inequity drivers and leadership | SDoH; Health in All Policies, etc. |  |
| Fene 2025 | Child vaccination in LAC | Social Determinants of Health (SDoH) |  |
| Foryciarz 2025 | Chronic Kidney Disease (CKD) | SDOH; Community-based system dynamics | Causal Modeling in a Participatory Framework |
| Frakt 2020 | Medical Debt & Health Access | Social Determinants of Health (SDoH) |  |
| Friedman 2021 | Overdose & COVID-19 Disparities | SDoH; Structural Racism (Jones) |  |
| Friedman J 2019 | Abortion Access & Legality | ILE normative and administrative framework; SDoH |  |
| Ghosh 2021 | Hospital Length of Stay (LOS) | Social Determinants of Health (SDoH) |  |
| Gillman 2024 | Health Equity & Barriers to Care | Social Determinants of Health (SDoH) |  |
| Glazer 2021 | Maternal Mortality & Mental Health | Health Equity; Quality Improvement |  |
| Golembeski 2021 | Carceral & Climate Crises | SDoH; Intersectionality |  |
| Gompers 2025 | Stroke Mortality & Structural Racism | Structural Racism; Intersectionality |  |
| Guilamo-Ramos 2020 | HIV Crisis & National Response | SDoH; HIV Care Continuum |  |
| Halbert 2023 | Cancer Risk & Social Drivers | SDoH; Stress Responses |  |
| Hamad 2025 | Poverty & Economic Policies | Fundamental Cause Theory; SDoH |  |
| Harper 2023 | Pulmonary Disease Interventions | SDoH; NIMHD Framework |  |
| Harrison 2025 | Infant Mortality & Societal Health | Social Determinants of Health (SDoH) y en la Epidemiología Social. |  |
| Hartinger 2024 | Climate Change LAC | SDoH; Planetary Health; Socio-Technical Systems |  |
| Hatzenbuehler 2013 | Stigma | Structural Stigma Theory |  |
| Hing 2024 | Structural Racism Measurement | Ecosocial Theory; SDoH |  |
| Holden 2022 | COVID-19 & Structural Racism | Structural Racism; SDoH |  |
| Huicho 2020 | Stunting Reduction in Peru | UNICEF Malnutrition Framework; SDoH |  |
| Hurwitz 2024 | Cardiovascular Care | Intersectionality; SDOH |  |
| Ingraham 2021 | COVID-19 Hospitalizations | SDoH; Area Deprivation Index (ADI) |  |
| Javed 2023 | CVD & All-Cause Mortality | SDoH; Fundamental Cause Theory |  |
| Jean-Francois 2020 | Maternal Mortality & Health IT | SDoH; NIMHD Levels of Influence |  |
| Jenkinson 2024 | Vaccine Hesitancy & Homelessness | Ecosocial Theory; Structural Violence |  |
| Jernigan 2025 | Life Expectancy & AI/AN Health | Nuka System of Care model for Ans; SCIIHE framework | Planetary and Indigenous Determinants of Health Framework |
| Ji Xu 2022 | Childhood Cancer Disparities | DisparitiesSDoH; NIMHD Influence Levels |  |
| Kavanagh 2021 | National Health Plans in the Americas | Social Determinants of Health (SDoH) |  |
| Keener 2025 | COVID-19 in K-12 Schools | Implementation Science |  |
| Kegler 2022 | Rural Health Equity Evaluation | Logic Model; Community Readiness Model |  |
| Kepper 2024 | Digital Health Equity | ConNECT; Levesque Access Framework; Digital Health Equity Framework | Integrated Digital Health Equity Adapted Model |
| Kerr 2018 | HIV Stigma & Neighborhoods | SDoH; HIV Stigma Theory |  |
| Klugman 2019 | Lung cancer Survival Disparities | Epidemiological Risk; SDoH |  |
| Kovach 2019 | Family Physician Equity Strategy | Social Determinants of Health (SDoH) |  |
| Lacey 2023 | Adult Trauma Outcomes | SDoH (Healthy People 2030) |  |
| Lavell 2023 | Urban Disaster Risk (Lima) | Social Construction of Risk; SDoH |  |
| LeBrón 2025 | Post-Pandemic Funding & CBOs | SDoH; Promotores/as models |  |
| Lee 2023 | COVID-19 Care Inequities | SDoH; Implicit Bias Theory |  |
| Lewis 2025 | Communication & COVID-19 Disparities | SDoH; Structural Drivers |  |
| Little 2020 | Dietary transition among Inuit in the Canadian Arctic. | Food Security and Dietary Transition Framework. |  |
| Lobelo 2021 | COVID-19 Racial Disparities in Georgia | SDoH; Clinical Epidemiology |  |
| Loehrer AP 2023 | Breast Cancer Surgical Disparities | SDoH |  |
| Martínez Leal 2023 | COVID-19 Vaccine Attitudes | Socioecological Model (SEM); CBPR |  |
| Martínez-Cardoso 2020 | Diabetes Management in Immigrants | Social Determinants of Health (SDoH) |  |
| Masters, 2022 | COVID-19 Vaccine Drivers | BeSD Framework (WHO) |  |
| Mathews 2021 | Cardiac Rehabilitation Disparities | Social Determinants of Health (SDoH) |  |
| Menza 2025 | Cardiac rehab | Social Determinants of Health (SDoH) |  |
| Mercado 2024 | Digital Health & SDoH | CDC’s SDOH framework | Structural and Systemic Drivers (SSD) Model |
| Mikhail 2018 | Trauma Care Disparities | SDoH; Socioecological Model | The Social Determinants of Trauma: A Trauma Disparities Framework |
| Morey 2024 | Data Equity & NHPI Health | Social Drivers of Health (SDoH) | Framework for evaluating community accessibility of data |
| Mourali 2025 | Algorithmic Fairness Perception | Distributive Justice Theories |  |
| Mujahid 2023 | Maternal Morbidity & Neighborhoods | SDoH; Neighborhood Context |  |
| Mujica 2023 | Violent Death Inequalities | Health Equity Monitoring (PAHO/WHO) |  |
| Mustanski 2015 | HIV Racial Disparities & Networks | Social Network Theory |  |
| Mustanski 2019 | HIV Disparities & Network Factors | Multilevel Risk Model; Social Network Theory |  |
| Nelson 2025 | Provider Burnout & Inequity | Racialized Organizations Theory |  |
| Nielsen 2024 | Biological Aging & Social Drivers | Geroscience; Social Exposome | Expanded Translational Geroscience Framework |
| Noaeen 2025 | Type 2 Diabetes & Neighborhoods |  | Integrated machine-learning and causal framework |
| Noel 2024 | Cancer Care Transitions | Inflammatory Bowel Disease (IBD) Disparities |  |
| Odufalu 2022 | Inflammatory Bowel Disease (IBD) Disparities | Social Determinants of Health (SDoH) |  |
| Parra Moreno 2025 | Migration & Ontological Insecurity | Framework of structural inequality; Relative Deprivation Theory; Structural Inequality |  |
| Petiwala 2021 | Community Voice & Collaboration | Cross-Sector Alignment Theory (RWJF) |  |
| Philbin 2018 | Immigration Policy & Latino Health | Social Determinants of Health; Socio-ecological Model | State-Level Immigration Policy Framework (pathways through which state-level immigration and immigrant-focused policies can impact Latino health) |
| Pinhero de Souza 2020 | University Participation and Health System | Brazilian Sanitary Reform; Freirean Pedagogy |  |
| Quinn 2024 | HIV Care & Anti-Black Racism | Minority Stress Theory; Structural Racism |  |
| Quinn 2024 | Anti-Black racism, mental health, and HIV care outcomes | HIV care model of Quinn et al. (2021) |  |
| Razdan 2024 | NICU Disparities & Quality Improvement | SDOH | NICU Disparity Etiology and Solutions Framework. Framework of family-centered care |
| Saigí 2021 | International Telemedicine | Diffusion of Innovations; Technology Acceptance Modeling, etc. | Explanatory framework of the key factors for successful international telemedicine |
| Salgado de Snyder 2021 | COVID-19 in Latinos | Social Determinants of Health (SDoH) | Adapted SDoH Latino COVID-19 Conceptual Model |
| Salvalaggio 2021 | Organ Transplantation & Geography | Final Rule (HHS); Allocation Equity Principles |  |
| Sanogo 2024 | Breast Cancer / Stress | Social Determinants of Health (SDoH); Allostatic Load |  |
| Savage 2024 | Political Advocacy & Health Policy | Political Determinants of Health (PDoH) |  |
| Schnake-Mahl 2020 | Gentrification | Neighborhood Effects; SDoH |  |
| Serchen 2021 | Discrimination in health | Social Determinants of Health (SDOH); Structural Racism | ACP Comprehensive Health Equity Policy Framework |
| Shahidullah 2023 | Integrated primary care as a lever for child health equity | Ecobiodevelopmental (EBD) Framework for Child Health Equity. |  |
| Sharifi 2016 | Childhood Obesity Disparities | Socio-ecological Model; SDoH |  |
| Socal 2020 | Health Judicialization in Brazil | Andersen and Newman Framework |  |
| Steinberg 2025 | CA-MRSA & Mass Incarceration | Structural Racism; Racial Residential Segregation |  |
| Taira 2019 | Social emergency medicine | SDOH; Teal & Street Model |  |
| Thornton 2023 | Child health equity | Social Determinants of Health (SDOH); Racism in Research |  |
| Thorpe 2025 | Racism & Biological Pathways | Weathering Hypothesis; Allostatic Load; SDoH | Theoretical Model of Interdependent Inequities in SDoH |
| Towe 2020 | Public Attitudes on Health Equity | Social Determinants of Health (SDoH) |  |
| Toyobo 2022 | Obstetric Anesthesia Equity | SDOH; IOM Quality Framework |  |
| Trani 2025 | Dementia Risk & Structural Racism | Community-Based System Dynamics (CBSD) y Social Determinants of Health (SDoH). | Core Shared Model of the Dementia Risk System |
| Trivedi 2022 | Interventional Radiology Equity | Social Determinants of Health (SDoH); Structural Racism |  |
| Turan 2017 | HIV and stigma | Stigma Theory; Intersectionality, Minority stress model | Adapted Framework of HIV-Related Stigma Mechanisms |
| Uzzi 2025 | Opioid Overdose & Structural Racism | Intersectionality; Geospatial Analysis | Index of Neighborhood-Level Trajectories of Structural Racism |
| Van Doren 2023 | Medical Education & Equity | Health Justice; Structural Drivers | Health Justice Standards (HJS) Framework |
| Vance 2021 | Antiracism in Neonatal Care | Health Equity; Social Determinants of Health (SDoH) |  |
| Vaughan 2019 | Heart Disease Mortality Trends | Intersectionality; Weathering Hypothesis |  |
| Wainstein 2025 | Kidney Health Equity & Advocacy | Social and Structural Determinants of Health (SDoH) |  |
| Ward 2015 | Socioeconomic Factors & Obesity | Social Determinants of Health (SDoH) |  |
| Wesson 2022 | CKD Progression & Primary Care | Social Determinants of Health (SDoH) |  |
| WHO-PAHO 2019 | Health Equity in the Americas | SDOH (WHO); Health in All Policies |  |
| Williams 2021 | COVID-19 & Race Disparities | Syndemic Framework; SDoH |  |
| Wilpers 2025 | Fetal Conditions & Care Outcomes | Andersen Model; Donabedian Model | Fetal Condition Care and Outcomes (FCCO) Framework |
| Xia 2022 | SARS-CoV-2 & SDoH in Canada | Social Determinants of Health (SDoH); Gini Coefficient |  |
| Young 2025 | Health Professions Education | Critical Pedagogy; Structural Drivers of Health |  |
| Zheng 2022 | Heart Failure & Length of Stay | SDoH; GWTG-HF Registry; Hierarchical Models |  |
| Zirinsky 2021 | COVID-19 in Children & Disparities | Social Determinants of Health (SDoH) |  |

*	Of the 135 studies from the Americas, 130 reported using and/or creating a new FTM.
