# Supplementary material for: Drivers of health equity in the Americas: a scoping review
Source: Int J Equity Health. 2026 Sep 16;25:255. doi: 10.1186/s12939-026-03031-6 (PMC13695892; doi:10.1186/s12939-026-03031-6)
Supplement: Supplementary file 5 [file 12939_2026_3031_MOESM6_ESM.docx]

**Drivers of Health Equity in the Americas**

**Supplementary File 6. Adapted PROGRESS PLUS**

| Topic | 1 | 2 | 3 | 4 | 5 | 6 | 7 | 8 | 9 | 10 | 11 | 12 | 13 | 14 | 15 | 16 | 17 | 18 |
| --- | --- | --- | --- | --- | --- | --- | --- | --- | --- | --- | --- | --- | --- | --- | --- | --- | --- | --- |
| Racism / Racial Disparities | 23 | 18 | 14 | 16 | 13 | 9 | 6 | 6 | 9 | 7 | 4 | 3 | 2 | 2 | 1 | 0 | 0 | 7 |
| Chronic / Specific Diseases | 11 | 9 | 7 | 14 | 13 | 5 | 7 | 7 | 8 | 5 | 3 | 1 | 1 | 0 | 1 | 2 | 0 | 3 |
| Digital Health / AI | 4 | 3 | 2 | 6 | 4 | 3 | 1 | 1 | 4 | 2 | 2 | 1 | 1 | 0 | 0 | 0 | 2 | 0 |
| Gen. Inequalities & SDOH | 10 | 8 | 6 | 11 | 9 | 7 | 2 | 2 | 5 | 4 | 2 | 1 | 1 | 1 | 1 | 2 | 2 | 0 |
| COVID-19 | 12 | 10 | 7 | 10 | 10 | 7 | 6 | 6 | 2 | 4 | 5 | 0 | 1 | 0 | 1 | 2 | 1 | 0 |
| Maternal & Perinatal Health | 5 | 4 | 3 | 6 | 5 | 5 | 6 | 6 | 2 | 3 | 3 | 1 | 1 | 1 | 3 | 0 | 0 | 0 |
| Child Health | 4 | 3 | 2 | 5 | 5 | 3 | 1 | 1 | 4 | 3 | 2 | 0 | 0 | 0 | 0 | 1 | 2 | 0 |
| Trauma & Violence | 5 | 4 | 3 | 3 | 4 | 1 | 2 | 2 | 4 | 1 | 1 | 2 | 2 | 2 | 1 | 0 | 2 | 0 |
| Health Access & Provision | 1 | 1 | 1 | 2 | 2 | 1 | 0 | 0 | 0 | 0 | 0 | 1 | 0 | 0 | 0 | 0 | 0 | 0 |
| HIV / AIDS | 5 | 4 | 3 | 3 | 1 | 3 | 5 | 5 | 3 | 3 | 2 | 0 | 4 | 4 | 1 | 0 | 0 | 0 |
| Policy, Justice & Society | 5 | 4 | 3 | 5 | 3 | 5 | 3 | 3 | 3 | 1 | 2 | 0 | 4 | 2 | 0 | 0 | 2 | 4 |
| Others* | 23 | 18 | 14 | 26 | 24 | 12 | 12 | 12 | 10 | 12 | 10 | 2 | 4 | 2 | 2 | 5 | 4 | 4 |
| TOTAL (Column sum) | 108 | 86 | 65 | 107 | 93 | 61 | 51 | 51 | 54 | 45 | 36 | 12 | 21 | 14 | 11 | 12 | 15 | 18 |

**Legend & Variable Mapping**

- **Standard PROGRESS Categories:** 1=Ethnicity; 2=Culture; 3=Language; 4=Socioeconomic Status; 5=Place of Residence; 6=Education; 7-8=Gender & Sex (Unified analytical axis); 9=Age; 10=Social Capital; 11=Occupation.
- **PLUS Categories (Vulnerabilities & Transitions):** 12=Life Transitions (e.g., migration, hospital discharge); 13=Disability; 14=Discrimination (Sexual Orientation); 15=Type of Relationships (Power/asymmetry).
- **Emerging Structural Drivers:** 16=Community Participation & Engagement; 17=Political Coherence & Accountability; 18=Commercial Determinants & Digital Governance.
- ***Others:** Includes climate change, environmental justice, and specific data visualization methodologies.

**Technical Note: Rationale for Framework Adaptation**

This scoping review utilizes an adapted version of the PROGRESS-Plus framework to transition from a descriptive mapping of vulnerable populations to an operational analysis of health equity drivers. The modifications are justified as follows:

- **Granularity of Ethno-Racial Axes:** Standard "Race/Ethnicity" is disaggregated into Ethnicity (1), Culture (2), and Language (3). In the context of the Americas, this allows for a precise distinction between structural racism, cultural barriers, and systemic linguistic exclusion.
- **Relational Dimensions of Power:** Age (9) was moved to the core stratifiers, and Sexual Orientation (14) was explicitly categorized under "Discrimination". This shift ensures these factors are analyzed as axes of social stratification and structural stigma rather than mere demographic traits.
- **Integration of Gender and Sex:** Following expert recommendations and Cochrane standards, Categories 7 & 8 are synthesized as a single analytical axis to reflect the literature's tendency to treat them as a transversal force and avoid data fragmentation.
- **Emerging Structural Drivers:** Three new categories (16, 17, and 18) were added to capture contemporary "engines" of inequity, such as Political Accountability, Commercial Determinants, and Digital Governance. These represent the "how" of inequity, identifying the specific policy levers and institutional mechanisms required for systemic change.

This adaptation ensures that the framework functions not only as a tool for identifying populations at risk but as a diagnostic map of the modifiable forces that drive health inequities today**.**

**Variable** [**Definitions**](https://methods.cochrane.org/equity/projects/evidence-equity/progress-plus)

**Group 1: PROGRESS Core (Social Stratifiers)**

- Ethnicity, Culture & Language: Identification with specific ethnic groups, cultural practices, and the impact of linguistic barriers on service access.
- Socioeconomic Status: Indicators of wealth, income, or material assets.
- Place of Residence: Geographic location, including rurality and neighborhood characteristics.
- Education: Formal schooling level or educational attainment.
- Gender & Sex: Socially constructed roles and biological attributes (male, female, intersex) analyzed as an interacting force.
- Age: Impact of life stages (infancy, adolescence, aging) as a social stratifier.
- Social Capital: Networks, social support, and community cohesion.
- Occupation: Employment status, labor conditions, and workplace environments.

**Group 2: PLUS Factors (Vulnerabilities & Transitions)**

- Life Transitions: Factors involving migration, displacement, or institutional transitions.
- Disability: Physical, sensory, or cognitive impairments and institutional barriers.
- Sexual Orientation: Identification as LGBTQ+ or diverse sexual identities under the lens of structural discrimination.
- Type of Relationships: Impact of household structures or social bonds of power.

**Group 3: Emerging Structural Drivers (New Dimensions)**

- Community Participation: Documentation of collective agency and community-led equity initiatives.
- Political Accountability: Systemic governance, policy coherence, and institutional responsibility.
- Commercial & Digital Drivers: Impact of private sector strategies, algorithmic governance, and data sovereignty.
